# Interruption of p53-MDM2 Interaction by Nutlin-3a in Human Lymphoma Cell Models Initiates a Cell-Dependent Global Effect on Transcriptome and Proteome Level

**DOI:** 10.3390/cancers15153903

**Published:** 2023-07-31

**Authors:** Konstantina Psatha, Laxmikanth Kollipara, Elias Drakos, Elena Deligianni, Konstantinos Brintakis, Eustratios Patsouris, Albert Sickmann, George Z. Rassidakis, Michalis Aivaliotis

**Affiliations:** 1 Institute of Molecular Biology and Biotechnology, Foundation of Research and Technology, 70013 Heraklion, Greece; kpsatha@auth.gr (K.P.); delena@imbb.forth.gr (E.D.); 2 Department of Pathology, Medical School, University of Crete, 70013 Heraklion, Greece; hdrakos@med.uoc.gr; 3 First Department of Pathology, National and Kapodistrian University of Athens, 15772 Athens, Greece; epatsouris@epipsi.gr; 4 Functional Proteomics and Systems Biology (FunPATh), Center for Interdisciplinary Research and Innovation (CIRI-AUTH), 54124 Thessaloniki, Greece; 5 Leibniz-Institut für Analytische Wissenschaften–ISAS–e.V., 44139 Dortmund, Germany; laxmikanth.kollipara@isas.de (L.K.); sickmann@isas.de (A.S.); 6 Institute of Electronic Structure and Laser, Foundation for Research and Technology—Hellas, 71110 Heraklion, Greece; kbrin@iesl.forth.gr; 7 Department of Chemistry, College of Physical Sciences, University of Aberdeen, Aberdeen AB24 3FX, UK; 8 Medizinische Fakultät, Medizinische Proteom-Center (MPC), Ruhr-Universität Bochum, 44801 Bochum, Germany; 9 Department of Oncology-Pathology, Karolinska Institute, 17164 Stockholm, Sweden; georgios.rassidakis@ki.se; 10 Department of Hematopathology, The University of Texas M.D. Anderson Cancer Center, Houston, TX 77030, USA; 11 Department of Clinical Pathology and Cancer Diagnostics, Karolinska University Hospital, Solna, 17176 Stockholm, Sweden; 12 Basic and Translational Research Unit, Special Unit for Biomedical Research and Education, School of Medicine, Aristotle University of Thessaloniki, 54124 Thessaloniki, Greece; 13 Laboratory of Biological Chemistry, School of Medicine, Faculty of Health Sciences, Aristotle University of Thessaloniki, 54124 Thessaloniki, Greece

**Keywords:** proteomics, transcriptomics, p53, MDM2, systems biology, protein interactions, lymphoma, nutlin

## Abstract

**Simple Summary:**

The activation of wild-type p53 protein in human lymphoma is a promising therapeutic strategy. A precise interruption of a protein–protein interaction, such as the p53-MDM2 interaction by nutlin-3a may lead lymphoma cells to apoptotic death through the stabilization and activation of p53. Despite this well-characterized scientific fact, there are lymphoma types that prevent or delay apoptosis by still unknown or purely understood mechanisms. Even lymphoma types that were initially affected by this interruption, finally managed to avoid death. An in vitro integrative comparative multi-omics analysis of different lymphoma types, before and after, wild-type p53 activation is a powerful way to shed light on such molecular mechanisms. Our findings provide deep holistic qualitative and quantitative molecular screening that highlights the putative role of specific proteins and pathways in lymphoma pathogenesis and shed light on the global effect of nutlin-3a. This information is the basis for a targeted and detailed study on specific proteins and pathways involved in lymphoma progression and resistance.

**Abstract:**

In most lymphomas, p53 signaling pathway is inactivated by various mechanisms independent to p53 gene mutations or deletions. In many cases, p53 function is largely regulated by alterations in the protein abundance levels by the action of E3 ubiquitin-protein ligase MDM2, targeting p53 to proteasome-mediated degradation. In the present study, an integrating transcriptomics and proteomics analysis was employed to investigate the effect of p53 activation by a small-molecule MDM2-antagonist, nutlin-3a, on three lymphoma cell models following p53 activation. Our analysis revealed a system-wide nutlin-3a-associated effect in all examined lymphoma types, identifying in total of 4037 differentially affected proteins involved in a plethora of pathways, with significant heterogeneity among lymphomas. Our findings include known p53-targets and novel p53 activation effects, involving transcription, translation, or degradation of protein components of pathways, such as a decrease in key members of PI3K/mTOR pathway, heat-shock response, and glycolysis, and an increase in key members of oxidative phoshosphorylation, autophagy and mitochondrial translation. Combined inhibition of HSP90 or PI3K/mTOR pathway with nutlin-3a-mediated p53-activation enhanced the apoptotic effects suggesting a promising strategy against human lymphomas. Integrated omic profiling after p53 activation offered novel insights on the regulatory role specific proteins and pathways may have in lymphomagenesis.

## 1. Introduction

The p53 protein stands in the core of an evolutionary conserved tumor suppressor pathway vital for protection from cancer development [1]. Upon stimulation by a variety of stress conditions, p53 orchestrates a wide spectrum of cellular responses to prevent any critical cell damage inherited to the next generation, mainly through activation of DNA repair mechanisms, reversible or irreversible cell-cycle arrest (senescence), modification of the cellular metabolism, and programmed cell death (apoptosis) [1]. The choice between the above responses to stress, depends on cell-type, cell-cycle stage, extent of DNA damage, p53 levels and isoforms stoichiometry, and external survival factors [1]. In most lymphomas, p53 signaling pathway seems to be inactivated or deregulated due to dysfunction or inactivation of wt-p53, resulted mostly by deregulation of its main negative regulator, MDM2, that may facilitate cancer development and progression in several tumor types, including lymphoma [1]. MDM2 is transcriptionally regulated by p53, creating an auto-regulatory feedback loop that regulates p53 activity and stability [1,2]. MDM2 binds to p53, preventing its interaction with the transcriptional machinery and inducing ubiquitin-dependent p53 degradation [3]. Nutlin-3a (N3a) was the first small molecule reported to specifically inhibit the p53-MDM2 interaction, inducing apoptotic cell death in various types of tumor cells harboring wt-p53, while generating minimal and, mostly, reversible effects in normal cells [2]. Interestingly, the effects of N3a vary significantly, depending on the cancer and the cell-type [4]. This highly heterogeneous apoptotic response could possibly be due to a downstream block of the apoptotic machinery in unresponsive cancer cells. Furthermore, recent studies have reported N3a exerting p53-independent effects [5,6]. Therefore, although N3a is currently one of the most-studied p53-related small molecules in cancer therapy, exhibiting promising efficiency in treating hematologic malignancies, it is becoming increasingly clear that its anti-cancer efficacy requires moving beyond the multiple molecular mechanisms involved in lymphoma development and progression along with the patient’s tumor genetic components [2]. Exploiting N3a effect in lymphomas tends to induce apoptosis rather than cell cycle arrest or senescence, favoring a cytotoxic rather than a cytostatic outcome, providing an excellent research tool for the comprehensive map of activated p53-induced apoptosis and the mechanisms implicated in lymphomagenesis. Moreover, studies on differential transcriptomic and proteomic profiling of lymphoma entities hold great promise in the study of deregulated proteins involved in lymphomagenesis [7,8]. We have previously shown that N3a-mediated p53 activation induces significant antitumor activity in cHL and NHL, affecting cell cycle, apoptosis, and proliferation through different mechanisms [9,10,11,12]. In the present study, the consequences of N3a-induced p53 activation were investigated on both transcriptome and proteome level of three different in vitro models of lymphoma subtypes, using state-of-the-art hypothesis-free systems biology approaches. Our work suggests that stimulation of the p53 signaling pathway response by N3a has a global effect on protein level and a more targeted effect on mRNA level in all wt-p53 lymphomas. p53 activation differentially affects proteins of key signaling networks and biological processes, such as p53 pathway, cell cycle, apoptosis, DNA damage response (DDR), PI3K/mTOR pathway, heat shock response (HSR), senescence, angiogenesis, autophagy, metabolism, OxPhos, oncogenic pathways, chromatin organization, and mitochondrial translation. A plethora of novel and already-known key proteins and pathways for lymphoma pathobiology are monitored in detail regarding their relative abundance. To our knowledge, no previous study to date has screened out the global impact N3a-induced p53 activation has in human lymphoma combining omics analyses.

## 2. Materials and Methods

### 2.1. Experimental Design and Statistical Rationale

At least three independent biological samples (per cell line; see Section 2.3) were analyzed using N3a (in all experiments), 17-AAG, Rapamycin and LY294402 reagents (in cell viability, proliferation assays), to ensure high confidence in protein detection and relative quantitation. All experiments included negative control cells, where DMSO alone was used at levels equivalent to the different reagent-treated cells. Each biological replicate included three technical replicates. Average value with a standard deviation was calculated for cell viability, proliferation, cell cycle and apoptosis assays. Qualitative evaluation of the additive, synergistic or antagonistic effectiveness of the drugs was determined calculating a combination index (CI) using CalcuSyn software version 1.0 (Biosoft, Ferguson, MO, USA), according to the Chou–Talalay method, as previously described (13). CI = 1 indicates an additive effect between two drugs, CI < 1 indicates synergy (low value suggests a stronger synergy) and CI > 1 indicates antagonism. For omics analyses, the detailed settings are described in each corresponding section.

### 2.2. Chemical and Biochemical Reagents

Unless indicated otherwise all chemicals were purchased from Sigma-Aldrich (Steinheim, Germany), Merck (Darmstadt, Germany), Applichem Biochemica (Darmstadt, Germany), Carl Roth (Karlsruhe, Germany) Roche Applied Biosystems (Mannheim, Germany), Amersham Biosciences (Piscataway, NJ, USA), BD Biosciences (San Diego, CA, USA), Gibco (Thermo Fisher Scientific, Germany), Invitrogen (Darmstadt, Germany) or Fluka (Buchs, Switzerland). N3a and HSP90-inhibitor 17-AAG were obtained from Cayman Chemical (Ann Arbor, MI, USA). mTOR inhibitor, Rapamycin and PI3K/mTOR inhibitor, LY292004 were purchased from Calbiochem (San Diego, CA, USA). Protease inhibitor cocktail and the BCA kit were obtained from Pierce Thermo Fisher Scientific (Schwerte, Germany) and the phosphatase inhibitor cocktails I/II A.G. Scientific, Inc, (San Diego, CA, USA). Benzonase Nuclease was purchased from Novagen, Merk (Darmstadt, Germany). The lysis buffer used was homemade RIPA Buffer: 150 mM NaCl, 1.0% *w*/*v* IGEPAL^®^ CA-630, 0.5% *w*/*v* sodium deoxycholate, 0.1% *w*/*v* SDS, and 50 mMTris, pH 8.0. Bradford reagent was from Bio-Rad (Hercules, CA, USA). Western blot analyses were conducted using Bio-Rad reagents unless indicated otherwise. The ICPL-labeling kit came from Serva Electrophoresis (Heidelberg, Germany) and trypsin enzyme from Promega (Mannheim, Germany).

### 2.3. Cell Lines, Drug Treatment and Chemical Reagents

Three wt-p53 human lymphoma cell lines, MDA-V (cHL), JMP-1 (NHL; MCL), and SUP-M2 (NHL; ALK + ALCL) were used in omics analyses and cell viability, proliferation, cell-cycle, apoptosis assays, while mutant p53 SUDHL-1 (NHL; ALK + ALCL) was used as a control in the mTOR-inhibition assay (Table S1). From the commercial human lymphoma cell lines, JMP-1 (established from pleural fluid) was purchased from ATCC (catalog number CRL 3378), while SUP-M2 (established from cerebrospinal fluid) and SUDHL-1 (established from pleural effusion) were purchased from DSMZ (catalog number, ACC 509, ACC 356, respectively). MDA-V cell line was established from a previously untreated patient’s lymph node diagnosed with Ann Arbor stage-I classical Hodgkin lymphoma at the University of Texas M.D. Anderson Cancer Center [12]. Cells were maintained in RPMI 1640 medium under standard conditions of exponential growth (10% *v*/*v* fetal bovine serum, 100 mg/L streptomycin, and 100 U/mL penicillin, in a 37 °C humidified incubator of 5% CO_2_). N3a, 17-AAG, Rapamycin and LY294402 reagents were added to cell cultures diluted in DMSO, when cell viability was estimated >90%, in different concentrations and time points, as indicated [11]. Original/authenticated cells stocks of the above cell lines were stored in liquid nitrogen, and when they were cultured to be used in the current study, they were obtained from these original stocks and thawed within two months of the performed experiments.

### 2.4. Cell Viability, Proliferation, Apoptosis Assays

Cells treated with N3a, 17-AAG, Rapamycin and LY294402 reagents at indicated concentrations, were evaluated for viability by trypan blue exclusion cell counts and proliferation by MTT assay, as previously described [11]. Apoptosis was determined by flow cytometry (Becton Dickinson–Franklin Lakes, NJ, USA) based on Annexin V-FITC staining methods and the ModFit LT software (Verity Software House, Topsham, ME, USA) according to the manufacturer’s protocol, as previously described [11].

### 2.5. Cell Cycle Analysis

Cell cycle analysis was performed as previously described [11], using a FACS Calibur flow cytometer (Becton Dickinson Immunocytometry Systems, San Jose, CA, USA), according to the manufacturer’s instructions (BD Biosciences, San Jose, CA, USA). Raw data were analyzed using the ModFit LT software (Verity Software House, Topsham, ME, USA).

### 2.6. Cell Lysis and Western Blotting

Cell lysates, 50 μg of total protein per cell line (treated/non-treated), prepared in RIPA buffer with protease/phosphatase inhibitors, were resolved in SDS-PAGE gels and electroblotted onto nitrocellulose or PVDF membranes, as previously described [11]. Protein concentration of samples was determined by BCA assay according to the manufacturer’s protocol [13]. Antibodies used included: LC3A/B (12741), MDM2 (86934), p21 (2947), HSP70 (4876), HSP90 (4874), Atg4B (5299S), eiFGI (8701), eiFGE (2067), p4EBP1 (2855), mTOR (2983), pmTOR (2971), S6 (2317), pS6 (4858), AMPKa (2532), p70S6K (9202) and secondaries IgG HRP linked antibodies (7074S/7076S) (Cell Signaling Technology, Bioline, Athens, Greece); TIGAR (166290), Ikaros (398265), p53 (126), APG5 (133158), NFκΒ-p65 (8008) (Santa Cruz Biotechnology, Santa Cruz, CA, USA), pS6 (67778-1-AP), 4EBP1 (60246-1-AP), mTOR (66888-1-AP), pmTOR (67778-1-AP) (Proteintech, Europe) and β-actin (MAB1501) Merck-Millipore, LabSupplies, Athens, Greece. Signal visualization of detected proteins was achieved using ECL blotting (Immobilon^®^ ECL Ultra Western HRP Substrate, WBULS0500, Merck-Millipore, LabSupplies, Athens, Greece) according to manufacturer’s protocol, using the ChemiDoc MP Imaging System Chemidoc (Bio-Rad Laboratories, Interab, Athens, Greece). Experiments were repeated at least two times (biological replicates). Images were analyzed and quantified with Image Lab Software Version 6 (Bio-Rad Laboratories). All the uncropped images are available in “2023_Cancers_Supplemental_Uncropped_raw WBs” and all the intensities and ratios of the resulted bands are available in Table S13.

### 2.7. Mitochondrial Labelling Assay—Measurement of ROS Generation via High Content Imaging

To evaluate higher membrane potential and the direct generation of ROS, cells, after N3a treatment for 24 h, were incubated with the Mitotracker^®^ Red CM-H2XRos (0.5 ng/µL), which specifically detects the ROS generation in mitochondria [13]. Following incubation (30 min in the dark, 37 °C), the accumulation of ROS was detected with High content imaging system (HCI). Images were captured with a 40× lens (Olympus–Shinjuku City, Tokyo, Japan) with the Operetta High Content screening microscope (PerkinElmer, Waltham, MA, USA) and analyzed using Harmony software 4.1 with PhenoLOGIC (PerkinElmer). Segmentation of the cells was based on the Hoechst dye. The intensity of mitotracker was calculated on the defined cell area. Cells that formed aggregates, thus with no clear cell boundaries, were excluded from the measurement. The classification of cells as live/dead was based on nuclei morphology in combination with the nuclear intensity of Hoechst stain and mitotracker. The experiments for mitotracker intensity were performed in three biological replicates with three technical replicates each. Statistical analysis t-Test was performed, approximately 10,000 cells for each sample, in GraphPad Prism 6 (Boston, MA, USA) between control and Nutlin 3a treated cells. For the statistical significance of cell death between Nutlin3a treated and control cells the biological and technical replicates were pooled and a Mann-Whitney test was performed using the online calculator at https://www.statskingdom.com/.

### 2.8. Transmission Electron Microscopy Imaging of the Biological Samples

Cells were fixed in 2.5% *v*/*v* glutaraldehyde for 24 h at 4 °C, washed in 0.1 M sodium cacodylate buffer, pH = 7.4, post-fixed in 2% *w*/*v* OsO4 in 0.1 M sodium cacodylate buffer for 60 min at 4 °C, and dehydrated in increasing concentrations of alcohol. The samples were impregnated with prophylene oxide and embedded in epoxy resin embedding media. Then ultra-thin sections were cut with LKB ultratome V-2088 and were placed on a transmission electron microscopy (TEM) grid, post-stained with uranyl acetate and lead citrate. Images were obtained using on a LaB6 JEOL 2100 electron microscope (JEOL Ltd., Akishima, Tokyo, Japan) operating at an accelerating voltage of 80 kV.

### 2.9. Affymetrix Microarray Analysis

Total cellular RNAs were isolated from the three lymphoma cell lines cultured in triplicate, before and after treatment with N3a, using the Trizol reagent (Invitrogen, Carlsbad, CA, USA). RNA’s concentration was determined photometrically (Nanodrop 1000, Thermo Scientific, Schwerte, Germany) and checked for its quality with the Agilent BioAnalyzer 2100 (Agilent, Waldbronn, Germany). RNA with yield >2 μg and A260/280 1.8 was used for further proceedings. cDNA/cRNA preparation and biotin labeling, subsequent hybridization on the GeneChip Human Genome U133 Plus 2.0 Arrays (Affymetrix Inc., Santa Clara, CA, USA), washing, staining, and scanning were executed according to the manufacturer’s recommendations. Affymetrix microarray data analysis included all array data preprocessing, normalization, statistical and comparative analyses, using DNA-Chip Analyzer (dChip2004), developed by Li and Wong at Harvard University and GeneTRAIL database. The gene expression values were obtained based on Perfect Match (PM) model only. Hierarchical cluster analysis for the array data set was performed to check arrays quality. The analysis was carried out using two sample *t*-tests. By modeling the resulting *p*-values, setting False Discovery Rate (FDR) = 50%, and using the mRNAs with a ≥2-fold change in expression, differentially expressed genes between control and treated cell lines were identified.

### 2.10. Isotope Coded Protein Labeling, Protein Fractionation by SDS-PAGE and in Gel Tryptic Digestion

Isotopic labeling of proteins, SDS-PAGE and in-gel tryptic digestion was conducted as previously reported [14,15,16] (see Supplementary Materials). In short, equal amounts from un-treated/treated cells obtained from the three lymphoma cell lines of interest were labeled with ^12^C-6-nicotinoyl-N-hydroxy-succinimide (^12^C-Nic-NHS) and ^13^C-Nic-NHS, respectively. The protein mixtures in concentrated samples were combined pair wise and fractionated by SDS-PAGE. Electrophoresis was carried out at 110–125 mA/gel, 200 V limit setting, for 35 min and the gel was stained with Coomassie [17]. Each gel-lane was excised into slices, in accordance with the number of protein bands revealed and were in-gel tryptic digested, as previously described [18]. Peptides were extracted from gel, desalted, speed-vacuum centrifuged, and diluted in 5% *v*/*v* FA for further mass spectrometric analysis.

### 2.11. Filter Aided Sample Preparation and in-Solution Tryptic Digestion

Gel-free sample preparation and proteolysis were performed using Filter Aided Sample Preparation (FASP) protocol [19] with minor changes (see Supplemental Information). Briefly, cell lysates were reduced by addition of 10 mM DTT at 56 °C for 30 min followed by alkylation of free thiol groups with 30 mM IAA at room temperature (RT) in the dark for 30 min. Subsequently, the lysates were diluted with freshly prepared 8 M urea-100 mM Tris-HCl, pH 8.5 [18] and placed on a Microsep centrifugal device (30 KDa, Millipore). The devices were centrifuged at 13,500× *g* RT for 20 min. All the following centrifugation steps were performed under similar conditions to eliminate residual SDS, and finally for the buffer exchange. To the concentrated proteins, sequencing grade trypsin was added and incubated at 37 °C for 14 h. The generated tryptic peptides were recovered by centrifugation followed by two consecutive washing steps with 50 mM TEAB, pH 8.5 and ultra-pure water. Finally, the peptides were acidified to pH < 3 using 10% *v*/*v* TFA and the digests were quality controlled, as described previously [19].

### 2.12. Mass Spectrometric Analysis by nLC ESI-MS/MS

Protein identification and relative quantitation by nLC-ESI-MS/MS was performed on three different systems, as described in detail in Supplemental Information. The sample preparation and the LC separation in the first two systems were performed as previously described [20] with minor modifications. Briefly, the tryptic peptide mixtures were separated on a reversed-phase column, packed in-house, using a pressurized packing bomb (Loader kit SP035, Proxeon). Tryptic peptides were separated and eluted in a linear water–acetonitrile gradient and injected into the mass spectrometer as previously described [20]. In the third system, peptides were concentrated on a C18 trapping column for 5 min followed by separation on a C18 main column with a 120 min LC gradient ranging from 3–42% *v*/*v* of 84% *v*/*v* can/water with 0.1% *v*/*v* FA at a flow rate of 250 nL/min. The detailed settings of the applied mass spectrometric methods are described in detail in the Supplemental Information.

### 2.13. Mass Spectrometric Data Processing, Protein Identification, and Relative Quantitation

The nLC-MS/MS raw data were processed as described in detail in the Supplemental Information. Briefly, they were loaded in Proteome Discoverer 1.3 (Thermo Scientific) and run using Mascot 2.3 (Matrix Science, London, UK) search algorithm against the Human theoretical proteome containing 161,566 entries (Uniprot release 2018_01) [21] and a list of common contaminants. For protein identification, the following search parameters were used: precursor error tolerance = 10 ppm, fragment ion tolerance = 0.5 Da, trypsin full specificity, maximum number of missed cleavages = 2 (in label free) or 3 (in ICPL), and the following as dynamic modifications: Acetyl (N-terminal), Oxidation (M), ICPL (N-Term) and ICPL (K) (in ICPL experiment). Carbamidomethylation was set as a fixed modification on every cysteine (C) in all samples. Arg-C was selected as the enzyme for the Mascot database search in ICPL experiments. Protein identification and quantification was achieved using peptides with a Mascot score of >20. The ratios of heavy to light ICPL labeled peptides were used for the relative protein quantitation. A false discovery rate (FDR) of 0.1% for proteins and peptides was required. Protein relative expression ratios were based on the peak area ratios of the peptides from the same protein identified in different samples. Fold changes in protein expression greater than 1.3 or less than −1.3 were determined to indicate significant proteins. Label free quantification was performed using the Progenesis LC-MS software from Nonlinear Dynamics (Newcastle upon Tyne, UK) version 4.1. The triplicate measurements of cell lines that were treated with N3a were compared to the corresponding treated/untreated triplicates separately. MS data processing including alignment of raw data, selection of the reference LC-MS run, and peak picking was performed automatically by Progenesis. The MS/MS spectra were exported as peak lists, searched against the Human theoretical proteome using Mascot 2.4 (Matrix Science), OMSSA 2.1.9, and X!Tandem Jackhammer (15 June 2013) with the help of searchGUI 1.14.4 [22] with the parameters described above. For combining the peptide and protein identifications obtained from the three search algorithms, we used our PeptideShaker software 0.28.0 [23]. The combined search results were filtered at an FDR of 1% and were exported using the advanced PeptideShaker. Only proteins that were quantified with at least two unique peptides were exported. Finally, for each protein, the average of the normalized abundances from the triplicate analyses was calculated to determine the ratios between the controls/untreated and the respective N3a treated samples.

### 2.14. Comparison of Transcriptome and Proteome Analysis

Microarray identifiers were converted into UNIPROT accession numbers using the DAVID Gene ID Conversion Tool (DICT), to generate one united reference data set, resulting from the transcriptome and proteome analyses of the three lymphoma subtypes before and after N3a treatment. Using these united omics data, the different samples were integrated, and compared according to the following:

1. Τhe altered transcripts and proteins between treated and untreated lymphoma samples in each cell type and in all lymphoma groups were compared, to identify the overlapping and unique genes/proteins between the two levels of analyses. The comparison was performed on pooled lymphoma data and on paired control-treated cells per cell line that derived separately from transcriptomics and proteomics analysis (proteomic experiments consisting of three independent biological replicates), to lessen the variability between the different samples.
2. The analysis of the deregulated GO terms and functional pathways in transcriptome and proteome data was performed according to Section 2.15. The main objective of this analysis was to identify the common and unique functional contexts extracted from the integrated omics analysis on the level of protein interaction networks.

### 2.15. Functional and Pathway Analysis of Deregulated Proteins

Functional and pathway enrichment analysis of both transcriptomics and proteomics data was as described in detail in the Supplemental Information using Perseus as part of the MaxQuant Software Package [24]. Categorical annotation was supplied by Gene Ontology (GO), DAVID (Database for Annotation, Visualization and Integrated Discovery), and KEGG pathway database [25,26]. Significance was tested by a *t*-test followed by a false discovery rate correction in the Benjamini–Hochberg procedure. Expression values for significant enriched annotations were summarized in an annotation score from −1 to 1 to represent levels of up- or down-regulation. GO terms were selected for further analysis and interpretation using gene count of two and Benjamini–Hochberg adjusted *p*-value ≤ 0.05, fold change ≥2. The most biologically significant genes were the ones with the best “enrichment” and “count of genes” values. Bibliographic search was performed in Pubmed (https://www.ncbi.nlm.nih.gov/pubmed (accessed on 1 February 2023)). A gene-interaction-network depicting direct and indirect interactions between the deregulated genes in the three lymphoma groups was generated from STRING using KEGG pathway database and UniProt database search [21].

### 2.16. Protein–Protein Interaction Network Analysis and Construction

For protein–protein interaction (PPI) data, the present study utilized a human PPI database from STRING [27]. The PPI network was constructed using Cytoscape [28] for visualizing, modeling, and analyzing the integration of bimolecular interaction networks with high-throughput expression data and other molecular states.

## 3. Results

### 3.1. N3a-Induced Transcriptome and Proteome Profile of the Three Lymphoma Types

**Figure 1:**
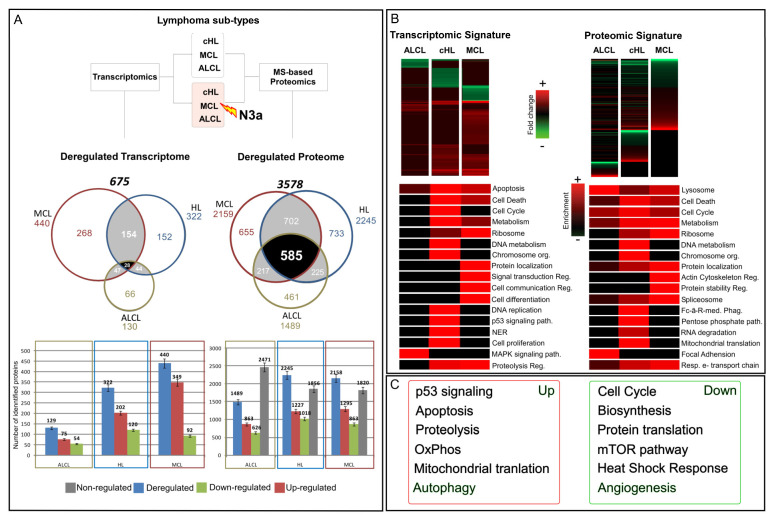
Overview of the results from the comparative transcriptomics and proteomics analysis on model lymphoma cell lines +/−N3a. (**A**): Venn diagrams of the overlapping deregulated mRNAs and deregulated proteins demonstrate the cell-specific effect of N3a on the lymphoma proteome and transcriptome. Bar diagrams show the exact number of proteins deregulated (in blue), up-regulated (in red) and down-regulated (in green) in cHL and NHLs. (**B**): The acquired data were hierarchically clustered, displaying in heat-maps the differential expression of mRNAs/proteins +/−N3a (FDR-adjusted, *p*-value < 0.05). Functional annotation analysis was combined with mRNA/protein expression level data to identify the top-GO-term enrichment categories (BP, CC and MF) relevant to N3a’s effect in both omics analyses. Columns were clustered according to top-ranked-categories of interest, and rows were clustered according to the lymphoma-subtype. Presence and enrichment levels were color-coded in a red-black gradient representing the −log10 (adjusted *p*-value) (red: presence in treated vs. control; black: missing value), with intensity proportional to presence/enrichment. (**C**): Selected pathways with a significant enrichment in proteins affected by N3a treatment indicating an up- or down-regulation of these pathways.

Previous work from our group has shown that the non-genotoxic induction of p53 signaling pathway by N3a has been associated with cell-cycle arrest and apoptotic cell death in wt-p53 containing lymphoma cells HL, MCL, and ALK + ALCL [9,11,12]. However, the global effect of N3a-induced p53 activation on mRNA and protein level has not been established in human lymphoma. To comprehensively map the signaling and regulatory events initiated by N3a-induced p53 activation, we employed a comparative transcriptomics and proteomics analysis in the three above mentioned lymphoma cell lines (Table S1), before and after N3a treatment (Figure 1). Prior to the omics analysis, we confirmed the previously described cellular and molecular N3a effects (Figure S10). All cells exhibited significant N3a dose-dependent decay in cell viability compared to the untreated cells (Figure S10C); p53 and MDM2 expression was significantly increased after N3a, as confirmed by Western blotting (Figure S10A); treated compared to untreated cells displayed an increasing cell number at G1 phase (Figure S10B); and apoptotic cells were significantly increased (Figure S10D). Interestingly, MDM2 and p53 showed different patterns in Western blots in different cell-types and conditions (+/−N3a) indicating the involvement of different isoforms in the different cell types.

**Figure 2:**
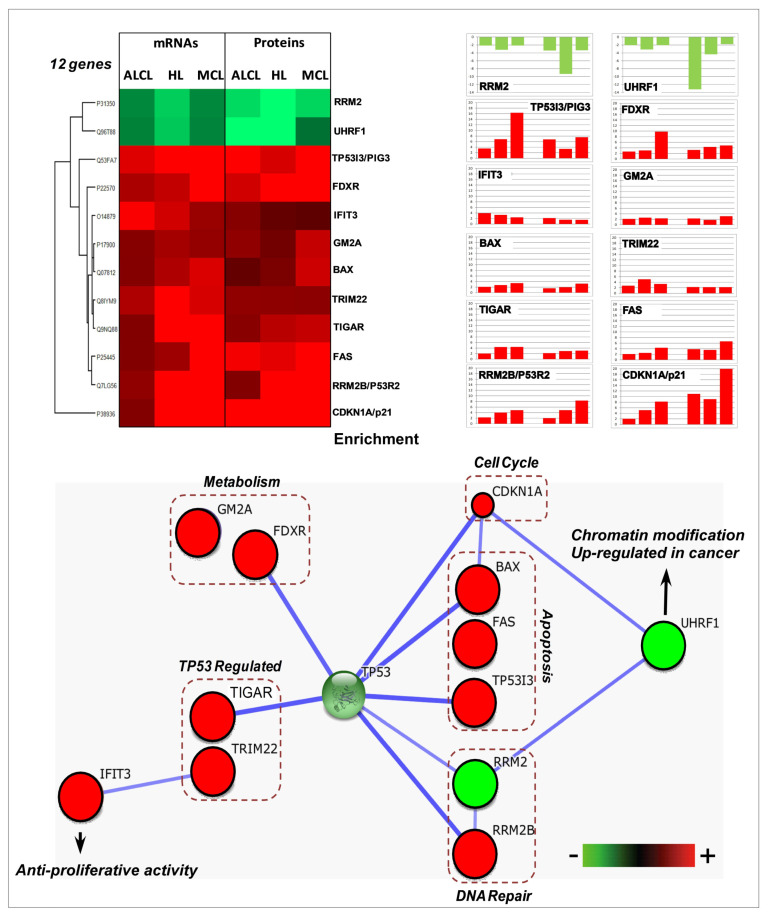
Common deregulated proteins and mRNAs in three lymphoma types: Heatmap and bar plot image depicting a common pattern of changes in mRNA and protein expression for the 12 genes that were commonly deregulated in both omics analyses, corresponding to the three lymphoma cell lines of our study (FDR-adjusted, *p*-value < 0.05). Protein interaction network of the common differentially expressed proteins using STRING is also shown.

Considering these results, the subsequent global comparative transcriptomics and proteomics analysis, performed in the three model cell lines (Figure 1A, Table S1) revealed in total 675 mRNAs and 3578 proteins affected in abundance by N3a, either positively or negatively (Figure 1 and Figure S1, Table S2). Interestingly, albeit the three lymphoma types shared more than 80% similarity on their expressed proteome identified in our study, the affected proteins in each lymphoma type were significantly different in both mRNA and protein level, with a very low overlap of 4% and 16%, respectively (Figure 1A and Figure S1A, Tables S5 and S6). Moreover, the two omics approaches had a total overlap of 216 common proteins, corresponding to almost 32% of the total deregulated transcripts and only 6% of the total deregulated proteins (Figure S1B). The overlap varied in the different cell-types, while only 12 proteins were found affected in both mRNA and protein level in all cell lines, with the same pattern of regulation (Figure 2).

### 3.2. Gene Ontology Terms and Pathway Enrichment Analysis of the N3a-Affected Transcripts and Proteins

Clustering and enrichment analysis of the N3a-affected proteins based on Gene Ontology (GO) and pathway terms, revealed that N3a has a global effect in all three lymphoma types, affecting multiple proteins in various cellular compartments in a different manner (Figure 1B,C and Figure S3). The enriched categories showed both similarities and significant differences on mRNA and protein level. HL and MCL cells had dramatically more enriched categories than ALCL in both omics data sets. Common enriched categories in both omics displayed the same enrichment pattern, but with distinct levels among the different lymphoma types (e.g., apoptosis, cell cycle). The uniquely enriched categories on mRNA and protein level included key biological processes, such as regulation of signal transduction and cell communication, cell differentiation and proliferation, p53 signaling pathway, regulation of proteolysis and protein stability, respiratory transport chain, and mitochondrial translation (Figure S3).

In HL cells, characteristic signatures on affected biological processes include metabolism, cell-cycle, chromosome organization, DNA metabolism and repair (mostly on mRNA level), NER (mostly on mRNA level), and mitochondrial translation (mostly on protein level) (Figure 1B and Figure S3A). Regarding the localization of the affected proteins, the main enriched categories were chromosome, non-membrane-bounded organelle, microtubule cytoskeleton, mitochondrion, and nuclear lumen (Figure S3B). The enriched protein molecular functions were histone and protein kinase activity, tubulin and kinase binding, DNA-dependent ATPase activity, signaling receptors and transferase activity, cytoskeletal protein, and structure-specific DNA binding (Figure S3C).

In ALCL cells, MAPK pathway was enhanced on mRNA level, whereas focal adhesion and lysosome pathway were mostly affected on protein level (Figure 1B and Figure S3D). The enriched categories in ALCL were lower compared to the other lymphoma cells, including enriched cytoskeleton and DNA packing proteins categories. Considering protein molecular functions, ALCL showed characteristic enrichment in HSPs, specifically in HSP70 protein binding, in protein phosphatase activity and ATP binding (Figure S3C). Moreover, as expected, the T-cell specific pathway of T cell receptor signaling pathway was significantly enriched in ALCL on protein level (Figure S3D).

MCL cells showed distinctive molecular signature on categories related to protein localization, ribosome, signal transduction, cell communication, cell differentiation, regulation of proteolysis and cytoskeleton (mostly on mRNA level), and protein stability, spliceosome (mostly on protein level), and OxPhos (Figure 1B and Figure S3C). The enriched cellular compartments were membrane-bounded and extracellular organelles, Golgi apparatus, extracellular region, ER lumen, and ribosome (Figure S3B). Enriched protein functions were isomerase activity, phospholipid binding, and ribosomal (Figure S3C).

### 3.3. Common Molecular Signatures after N3a Treatment in All Three Lymphoma Types

The common molecular signatures after N3a treatment among the three lymphoma types on both mRNA and protein level are shown in Figure 2. Twelve proteins responded to N3a following the same pattern of change on mRNA and protein level. Proteins with increased levels included protein related to the activation of p53 signaling pathway (TP53), cell growth arrest (p21—CDKN1A), apoptosis (BAX, FAS, TP53I3/PIG3), metabolism regulation (FDXR, GM2A, TIGAR), protein ubiquitinoylation (TRIM22), DNA repair (p53R2/RRM2B), anti-proliferative activity (IFIT3), and decreased levels of proteins associated with DNA replication (RRM2) and chromatin modification (UHRF1) (Figure 2).

The core deregulated mRNAs in all three lymphomas (28 mRNAs) were involved predominantly in TP53 signaling pathway, apoptosis, metabolic process, regulation of signaling, cell cycle checkpoints and in stress response, showing the same regulation pattern (Figure S2, Table S5). Most of the common mRNAs were increased, while only four and five mRNAs were decreased in HL/MCL and ALCL, respectively. The exception to this rule was the PREX2 transcript that was found decreased in ALCL and increased in HL and MCL.

**Figure 3:**
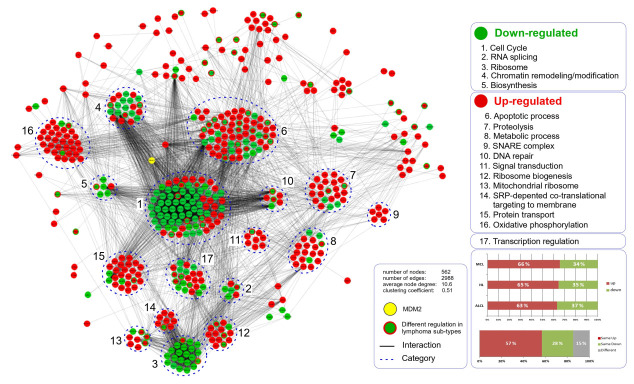
Protein interaction network of deregulated global common proteome profile of N3a-treated cHL and NHL lymphomas: Visualization of the common differentially regulated proteins (FDR-adjusted, *p*-value < 0.05) in cHL/NHL in a protein–protein interaction network using STRING and Cytoscape. Nodes indicate proteins and edges indicate the interactions between them. The up- (red) or down-regulation (green) is color coded for each protein (both colors: differential protein-regulation in the three lymphomas). Unsupervised clustering of lymphoma-subtype proteome alterations clustered the deregulated proteins into related functional classes, including down-regulated cell cycle, RNA splicing, ribosome, chromatin remodeling/modification apoptosis, proteolysis, metabolic processes, DNA repair, ribosome, and oxidative phosphorylation. Bar diagrams in the right bottom area of the figure represent the percentage of proteins commonly deregulated (up-regulated: red; down-regulated: green) or differentially deregulated (grey) in cHL/NHLs.

The common core of deregulated proteins involved 585 in total, identified in all three lymphoma types, covering a wide range of protein functions, pathways, and biological processes, and signifying a global cellular effect of N3a (Figure 3 and Figure S3, Table S6). Most of the deregulated proteins in all lymphoma types (~65%) showed elevated amounts, and ~85% of them showed the same pattern, corresponding to increased or decreased abundance after N3a. The remaining 15% that exhibited different patterns require further investigation, since these proteins will provide significant information about the lymphoma-type specific effect of N3a.

### 3.4. Unique Molecular Signatures of the N3a Effect in the Three Lymphoma Types

The plethora of uniquely identified and deregulated transcripts and proteins in each lymphoma provide valuable information on the lymphoma-specific pathobiology (Figure 1A and Figure S1, Tables S7 and S8). Interestingly, the enriched categories and pathways for each cell type differentiated on mRNA and protein level, highlighting distinct regulation events on transcription, translation, and protein maturation level. In HL, we found 152 unique mRNAs, 420 unique proteins, and 733 unique deregulated proteins, in MCL 268 unique mRNAs, 321 unique proteins, and 655 unique deregulated proteins, and in ALCL 66 unique mRNAs, 399 unique proteins, and 461 unique deregulated proteins (Figure 1A).

### 3.5. Cellular Pathways and Biological Processes Affected by N3a in the Three Lymphoma Types

The N3a-affected cellular pathways and biological processes showed similarities, but also significant differences in the three lymphoma types. Even the commonly affected pathways and processes presented different pattern or degree of differentiation in the three lymphomas (Figure 1, Figure 2, Figure 3, Figure 4, Figure 5, Figure 6 and Figure 7 and Figures S4–S9). Furthermore, in many cases, the effect was different on mRNA and protein level, indicating an alternative mechanism of protein regulation.

#### 3.5.1. Activation of p53 Signaling Pathway by N3a

**Figure 4:**
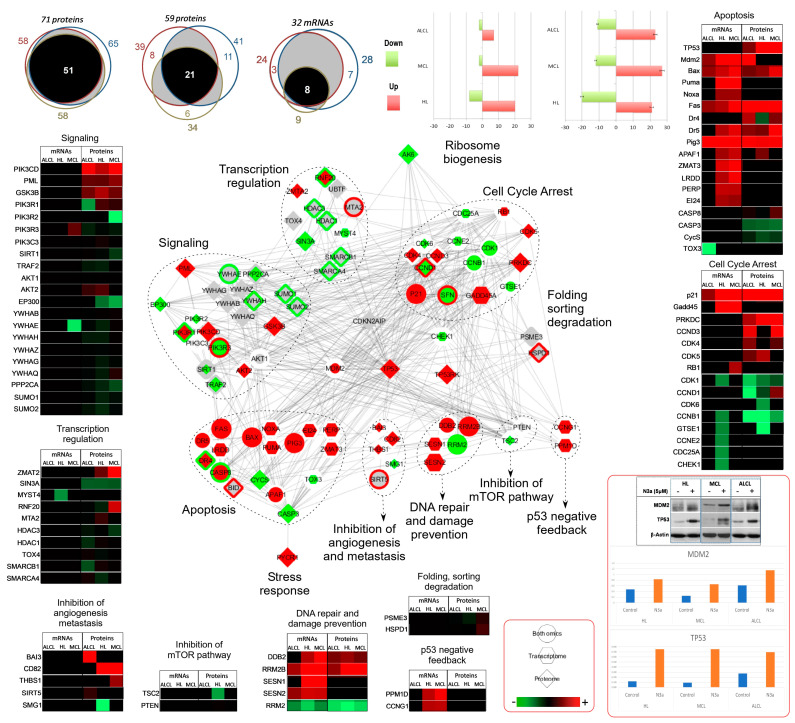
Integrative visualization of the N3a effect in p53 signaling pathway: Venn diagrams in the upper left half of the figure illustrate the number of the: (1) molecules in both omics datasets, (2) deregulated proteins and (3) deregulated mRNAs, emphasizing on the divergent effect N3a has on the lymphoma proteome and transcriptome. Color-code bar-plot images in the upper right half of the figure (red: up-regulation; green: down-regulation) depict an overall common pattern of differential expression at the mRNA/protein level for the deregulated p53-related-genes in both omics analyses, corresponding to the three lymphoma cell lines of our study. Integrative protein–protein interaction network of the N3a-effect on p53-related-genes is visualized in the center of the figure using STRING and Cytoscape. Nodes indicate proteins and edges indicate the interactions between them, while color depicts regulation (red: increase; green: decrease). Around the networks, representative p53-selected proteins clustered according to their involvement to other pathways are shown in green–red–black gradient, representing the −log10 (adjusted *p*-value) (red: higher abundance in treated; green: lower abundance in treated; black: missing value). Color intensity reflects differences in abundance levels. The western blot image of TP3 and MDM2 is also included with the bar graphs showing the ratio of each band after the densitometry analysis.

Integrated analysis of the two omics profiles revealed a total of 88 p53 signaling pathway-related proteins, with 77 of them significantly changed in abundance after N3a treatment (Figure 4, Table S9). These proteins are associated with cell-cycle arrest, response to stress, DDR, transcription regulation, TP53 negative feedback, protein folding, sorting and degradation, inhibition of angiogenesis and metastasis, ribosome biogenesis, and apoptosis (Figure 4; Table S2). N3a affected mRNAs and proteins differently and exhibited a cell-dependent pattern of differentiation. Only 15 of these proteins were common in all samples, in both omics, demonstrating the same differentiation pattern. Most of them increased in abundance after N3a treatment except for three proteins: CCND1 (oncogene, accused to be involved in B-lymphocytic malignancy, particularly mantle-cell lymphoma -MCL), RNF20 (involved in the pathway protein ubiquitination), and PSME3 (facilitates the MDM2-TP53 interaction, which promotes ubiquitination and MDM2-dependent proteasomal degradation of TP53), indicating an activated TP53 signaling pathway in all three lymphoma cells.

#### 3.5.2. N3a-Induced Effect on Cell-Cycle Pathway

**Figure 5:**
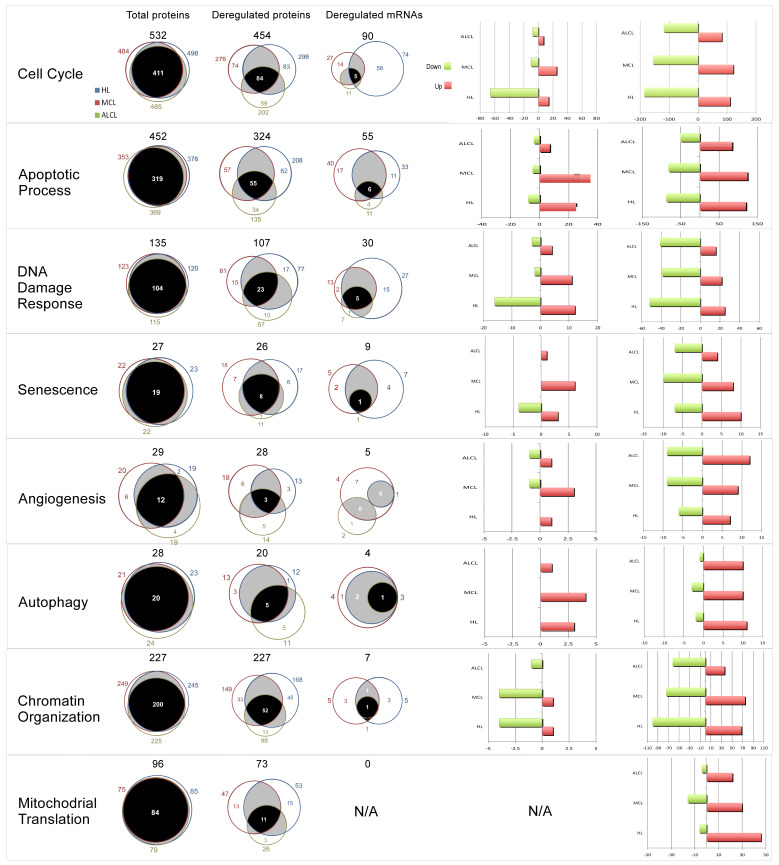
Regulation profile of key cellular pathways and biological processes by N3a in the three lymphoma types: The scheme summarizes N3a-mediated alterations in eight core cellular pathways in cHL/NHL cells. Each panel demonstrates the regulation profile of a cellular pathway post-N3a-treatment. Venn diagrams are generated as in Figure 4. Bar-plot images in the right half of each panel demonstrate the differential regulation status of mRNAs/proteins relevant to every cellular pathway, in each lymphoma cell line of our study (red: up-regulation; green: down-regulation).

Pathway analysis assigned 621 proteins under cell-cycle signaling pathway (101 mRNAs/583 proteins) with 62 proteins found in both omics analysis (Figure 2 and Figure 3, Figure 5, Figures S2 and S4A; Tables S2), indicating lymphoma type-dependent N3a-effect. Moreover, 38 and 583 proteins had only their transcript or protein levels altered, respectively, after N3a treatment. On the transcriptome level, HL showed dramatically higher deregulation, whilst on the proteome level all lymphomas showed similar deregulation of cell-cycle-related proteins. The overlapping transcriptomic and proteomic signature demonstrated the same expression pattern, with most of them showed a decrease. The overlapping mRNAs were in concordance with the corresponding protein levels in all samples.

Omic data further confirmed on molecular level the cellular data (Figure S10) that showed growth-arrest in all lymphomas, through changes on main cell-cycle regulators, such as, the increase in CDKN1A/p21 on both mRNA and protein level, and GADD45 on mRNA level, and the decrease in CCNB1 on both mRNA and protein level (Figure 4) and G1/S transition-marker DHFR on protein level.

Interesting proteins that exhibited increased levels after N3a, were PDS5B, a cohesion-associated protein and negative regulator of cell proliferation (on protein level in HL/ALCL), and the cell-cycle repressor E2F7 (on protein level in MCL). On the other side, interesting proteins that exhibited decreased levels after N3a were G1/S transition-marker DHFR (on mRNA level in HL, on protein level in all), Ikaros (on protein level in all) and pre-replicative/CMG components (MCM 2-7, GINS2-4, CDC6, ORC-3/-6) involved in eukaryotic DNA replication. Remarkably, the APC/C E3 ligase-complex subunits (ANAPC7, APC1, and APC3) were increased following p53 activation, previously found to induce mitotic arrest and apoptosis, suppressing tumorigenesis via decreased CDC20 (on mRNA level in HL) [29].

#### 3.5.3. N3a-Induced Deregulation of Proteins Involved in Apoptotic Process

In total, 452 affected apoptosis-related proteins were detected, 55 on mRNA level, and 423 on protein level (Figure 2 and Figure 3, Figure 5, Figures S2 and S4B; Table S2). Integrated analysis of the data revealed that 26 proteins are common in both omics, while 29 and 397 were uniquely altered in transcriptomic and proteomic datasets, respectively. The affected proteins include p53-dependent and -independent apoptosis-associated proteins: PIG3, BAX, NOXA, APAF1, PUMA, FAS, DR5, LRDD, and FDXR (increased); XIAP and BIRC5 (decreased). Some of the novel decreased proteins have been described to exert anti-apoptotic and survival effects in different malignancies [30,31,32], including cHL: LMNB1 (R) and DSG1 (P); in MCL: DFFB (R) and ITGB2 (P); and ALCL: HSPA7 (R) and CARD11 (P). Noteworthy, up-regulated proteins included the novel p53-responsive PDCD6 (P_all), which induces apoptosis, and KMT2A (P_ALCL), a hematopoiesis regulator promoting apoptosis, frequently involved in leukemogenic and B-cell NHL translocations [33].

#### 3.5.4. Deregulation of Proteins Involved in DNA Damage Response

Our study revealed 151 N3a-affected DDR proteins with increased key proteins indicating activated DDR: P53R2, PIG3, DDB2, XPC, GADD45, SESN1/2, PRKDC, H2AX, MRE11/Nbs1 (Figure 3, Figure 5, Figures S2 and S4C; Table S2). We suggest that activated p53 conferred reduction to expression levels of target genes, such as DNA repair genes ERCC5 (P_MCL), XRCC1 (P_HL/MCL), RAD23 (P_all) required for p53 degradation, E3 ubiquitin-protein ligase UHRF1 (R/P_all) and critical Homologous Recombination (HR) proteins RAD51 (R_all) and RPA (RPA1: P_all; RPA2: P_ALCL/cHL). These findings suggest a decreased HR repair activity, supporting the requirement of repressed HR for the induction of apoptosis in cells harboring irreparable DNA damage [34], which is pervasively present in hematological malignancies.

#### 3.5.5. p53 Activation Affects Angiogenesis, Autophagy, Metabolism, and Chromatin Organization in Lymphoma

Thirty-four senescence proteins were identified, including up-regulated p53-dependent effectors (TIGAR, p21, PIG3) and senescence markers (4 senescence pr GLB1) supporting a N3a-induced senescence in all lymphoma cell lines assessed (Figure 5; Table S2). Furthermore, the down-regulated HMGB2 and PLK1 in ALCL/cHL, indicate that p53 induced senescence possibly through their inhibition. Thirty-one angiogenesis-associated proteins were detected in all lymphomas (Figure 5; Table S2). We report up-regulated anti-angiogenic proteins in lymphoma after p53 activation, with BAI3 (ALCL), SEMA4F (cHL) and CD82 (MCL) showing the most striking differences. Furthermore, pro-angiogenic factors SEMA4D, EGLN1, and FOXK2 were down-regulated after N3a-treatment, with ECM1 demonstrating the lowest expression level in MCL. Concerning autophagy, 32 proteins were substantially altered, including increased autophagy-stimulating proteins (ATG4B, ATG5, P_cHL/MCL; SESN2, R_all) possibly cooperating with apoptosis to induce cell death (Figure 5 and Figure S11, Table S2). Our results suggest that p53 induced autophagy in lymphoma via increased level of PRKAB1 (R_MCL, P_cHL), DRAM (R_MCL/cHL), WIPI2 (P_all), and increased APOL1 (P_ALCL) and OPTN (P_MCL) mediating protein degradation. Immunoblotting further validated proteins ATG4B and ATG5, as well as LC3 (Figure S11).

Seeking to acquire valuable information monitoring autophagy (and apoptosis), we also examined cells for ultrastructural changes at nm resolution, before and after exposure to N3a (5 μM, 24 h), using TEM. The control group cells exhibited healthy normal appearing cytoplasm, nucleus, chromatin, and mitochondria (Figure S13). In contrast, N3a-exposed group cells had accumulated characteristic apoptotic features, such as increased nuclear shrinkage, chromatin condensation in the nuclear periphery, and damaged mitochondria (enlarged). Furthermore, N3a stimulated the formation of autophagosomal structures, such as autophagosomes containing lucent materials and dense organelles, and autolysosomes, with degraded vacuolar (Figure S13). Autophagosomes appeared close to the damaged mitochondria, having round and double membrane structures (Figure S13).

A total of 393 proteins, mostly increased, were clustered into metabolic pathways (Figure 3 and Figure S2; Table S2). Activation of p53 favored an increase in mitochondrial respiration chain protein complexes (NADPH, ATP, COX, UQC, SDH) and a reduced glucose metabolism, by inducing TIGAR, p53R2, GAMT (R_MCL, P_all), and AIF (P_all) and by decreasing LDHA (P_all), ALDOC (P_all), ENO2 (P_all), and O-GlcNAcylation markers (up-regulated OGT, P_all; decreased OGA, P_cHL/MCL), thereby enhancing OxPhos in the three lymphomas, especially in cHL/MCL.

Our analysis revealed 279 chromatin organization proteins, including decreased chromatin remodelers UHRF1, DNMT1, chromatin-remodeling complex ISWI members (BPTF, POLE3, CHRAC1, ACF1), the transcriptional repressor CTCF, cohesin subunits SA1/2, Polycomb-group proteins (PcG, EED), chromo-box readers (CBX1/3, CBX5), and the histone chaperone NASP (Figure 3 and Figure 5; Table S2).

### 3.6. The Effect of p53 Activation on Mitochondrial Translation

A total of 88 mitochondrial translation proteins were mostly increased after N3a-treatment, with 68 being mitochondrial ribosomal proteins (MRPs) (Figure 1, Figure 3 and Figure 5; Table S2). Remarkably, increased proteins included members of the pentatricopeptide repeat (PPR) essential for mitochondrial protein synthesis (MRPP3, PTCD3, LRPPRC, and MRPS27), pro-apoptotic mitochondrial ribosomal proteins (DAP3/MRPS29 and MRPL41) and p53-dependent cell-cycle arrest-related proteins (MRPL5, MRPL11, and LRPPRC). PTCD3, involved in the assembly of respiratory chain complexes and MRPL41 in p53-mitochondrion-dependent apoptosis, support a tumor-suppressive role in association with p53 [35].

### 3.7. Oncogenic Pathways Are Significantly Affected by p53 Activation

We identified changes in NF-kB/MAPK and Jak-Stat/BCR pathways pertinent to lymphoma pathogenesis (Table S2). Particularly, increased proteins included components of NF-kB (CD40, FAM62A/MBC2, MALT1, and TBK1), Jak-Stat (STAT1-6, JAK3 and StIP1/ELP2); MAPK (MEK2-3, ASK1, ERK1-2, and RSK1-2) and BCR (BLNK, LYN, PLCG2, PKCβ, and LAT2) pathways. Characteristic oncogene proteins displaying significantly decreased levels (NF-kB-related IKBε, NEMO, IKKβ, BAFF-R, Jak-Stat-related IL6R, and MAPK/BCR-related CXCR4), provide a possible alternative route to the p53-dependent apoptotic effect that merits further investigation.

### 3.8. The PI3K/mTOR Pathway

The detection of 92 N3a-affected PI3K/mTOR pathway-proteins suggest a reduced pathway activity through p53-dependent increase in the AMPKβ1/β2 subunits (PRKAB1/2, PRKAB1), SESN1/2 and decreased downstream PI3K/mTOR protein-targets RPS6KB2/p70S6K-beta (cHL) and rpS6 (all), eIF4GI (P_all) and eiF4B (R_MCL, P_all) (Figure 7 and Figure S7; Table S2). Among the novel N3a-sensitive PI3K/mTOR-reducing proteins in lymphomas, were increased PHLDA3 (R_cHL/MCL), OSMR (R_cHL), COL6A3, and Rictor (P_ALCL/cHL). Immunoblotting validated an overall decrease in mTOR downstream-targets p70S6K, rpS6, 4EBP1, eiF4GI. Decreased phosphorylation of mTOR and readouts of its signaling activity (p-4EBP1, p-rpS6) further supported the inhibitory effect of p53, as well as the p53-AMPK-mTOR crosstalk mechanism, especially in cHL/MCL. Interesting results that should be further studied in the future are associated with the different proteoforms in some of the proteins, such as 4EBP1, p-4EBP1, RPS6KB2, and eiF4GI.

### 3.9. Deregulation of Heat Shock Response (HSR) by N3a

**Figure 6:**
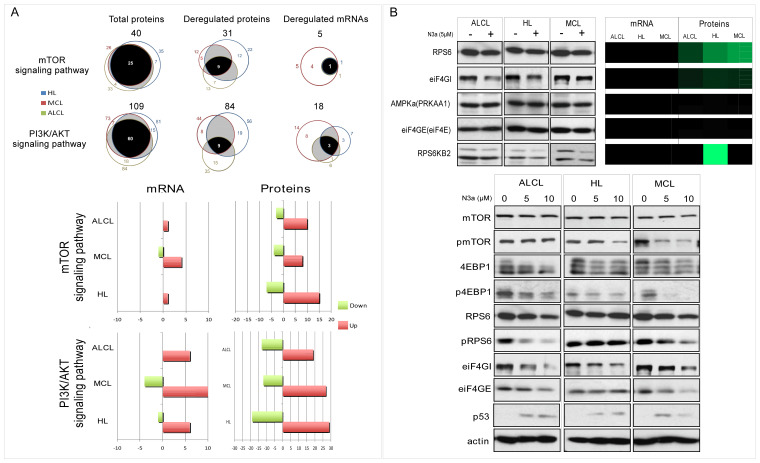
mTOR- and PI3K/AKT-related protein expression levels in N3a-treated cHL, MCL and ALK+ ALCL cells: (**A**): Venn diagrams and color-coded bar-plot images are generated as in Figure 4. Bar-plot images highlight the differential mRNA/protein levels for the mTOR-related- and PI3K/AKT-related- genes that are deregulated in both omics analyses, in each lymphoma cell line of our study (red: up-regulation; green: down-regulation). (**B**): Key mTOR pathway-proteins showed decreased or non-regulated expression levels. Proteomic results were validated using Western blot in cHL, MCL, and ALK+ ALCL cells +/−N3a. Stabilization and activation of wt p53 suppressed mTOR signaling in HL, MCL, and ALK+ ALCL cells. β-Actin protein expression was used as loading control. The calculated intensity and ratio of each band after the densitometry analysis are available in Table S13.

**Figure 7:**
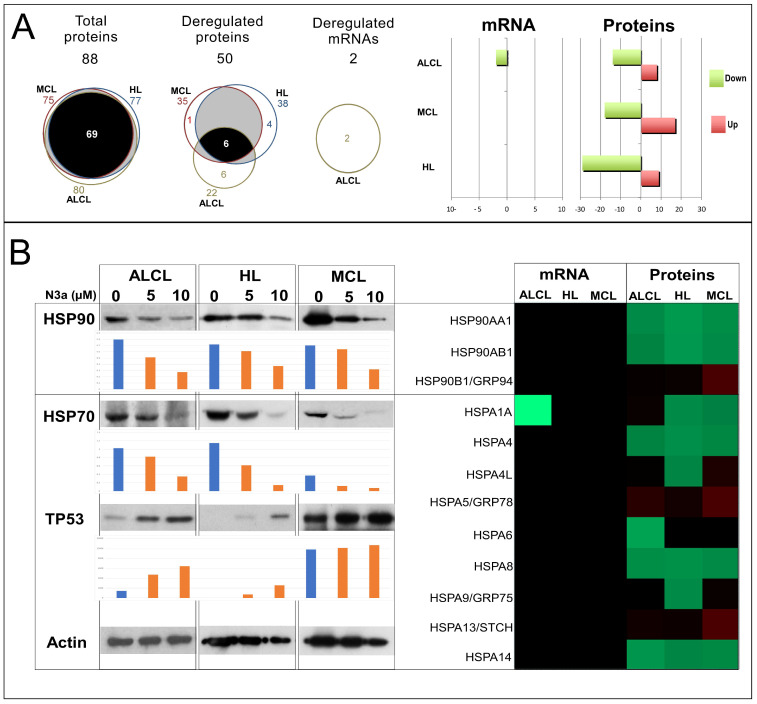
Heat shock proteins expression levels in N3a-treated HL, MCL and ALK + ALCL cells: (**A**): Venn diagrams and color-coded bar-plot images are generated as in Figure 4. Bar-plot displayed primarily a down-regulation of the HSPs-related-genes following N3a on the protein level in a lymphoma-cell-specific manner (red: up-regulation; green: down-regulation). (**B**): HSP70 (nine family members) and HSP90 (three family members) were found to have decreased protein levels by omics (right panel) and were validated using WB analysis. β-Actin protein expression served as a protein load and integrity control. The bar graphs showing the ratio of each band after the densitometry analysis are also included in this figure.

A total of 88 HSR-related proteins, including the HSPs and related (co)chaperones, were detected to be mostly decreased (Figure 6 and Figure S8; Table S2). Among the novel p53-dependent proteins in our analysis were four members of the HSP90/HSPC protein family (HSP90-α/-β, GRP94, and TRAP1) and nine HSP70 protein members, six of them being decreased: HSC70/HSPA8, HSPA1A/HSP1B, HSPA4, mitochondrial HSPA9/mortalin/Grp75, HSPA14, and HSPA6. Further immunoblot analysis confirmed reduced expression of HSP70/HSP90 in all N3a-treated lymphoma cells, especially in MCL/ALCL cells.

### 3.10. Synergistic N3a/Hsp90 Inhibition Effect on Lymphoma Cells

**Figure 8:**
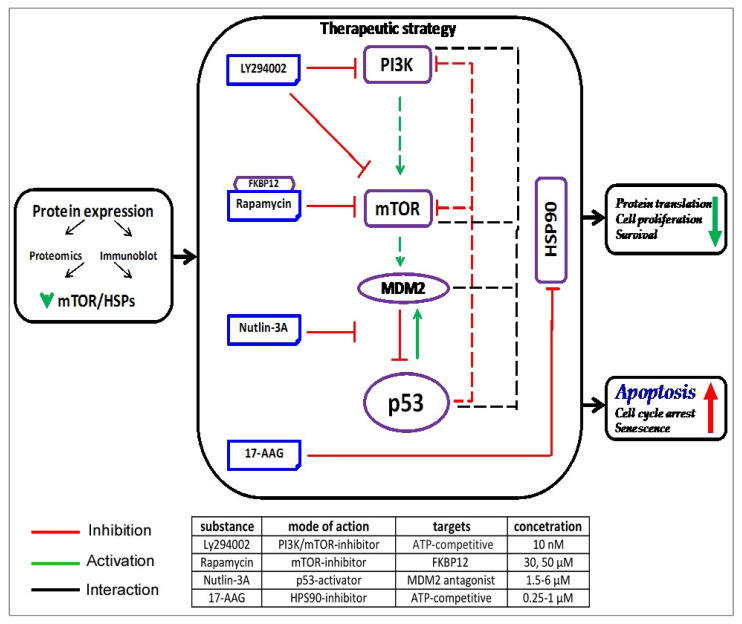
Synergistic effect of N3a with HSPs and mTOR inhibitors in lymphoma cells: Summary of the therapeutic scheme followed in our study, with major findings highlighted in bold. Color-coded arrows represent regulation (red: up-, green: down-). The signaling pathways in which PI3K, mTOR, MDM2, p53, and Hsp90 are participating, are in a continue balance of inter- and intra-activation and deactivation towards cellular homeostasis and fitness. Every imbalance in their regulation supports lymphomagenesis, therefore are targets of promising therapeutic strategies. In the present study we use synergistically four inhibitors of these proteins, namely LY294002, Rapa, N3a, 17-AGG, respectively, aiming to suppress lymphoma cell growth.

Based on previous results demonstrating that HSP90-inhibitor 17-AAG can synergize with N3a to induce p53-dependent apoptosis [36], we investigated this hypothesis in our in vitro system (Figure 8 and Figure S8A–C). In all cases, N3a/17-AAG combined treatment was more effective than each agent alone, and the combination index was consistently <1, indicative of synergy (Table S1). Simultaneous administration of N3a and 17-AAG showed highest synergistic effects on MCL/ALCL cells at the ED50 levels (CI_cHL_: 0.785, CI_MCL_: 0.239 and CI_ALCL_: 0.399; Figure S8D–F).

### 3.11. Enhanced N3a-Effect on Lymphoma Cells in Combination to PI3K/mTOR Inhibition

Previous publications report simultaneous inhibition of PI3K/mTOR signaling using mTOR inhibitor Rapamycin (Rapa) and/or PI3K inhibitor LY294002 with N3a-p53-activation reinforced induction of p53-mediated apoptosis in CTCL cells [37]. In this regard, the studied lymphoma cells were treated with low concentrations of N3a, LY294002, Rapa, or their combination (N3a/LY294002, N3a/Rapa), leading to a significant reduction in cell viability and cell growth (Figure 8 and Figure S9). Combined treatment with N3a/LY294002 for 48 h augmented the inhibitory effect of N3a causing the most striking decrease in ALCL (from 21% to 91%).

### 3.12. N3a Promotes Metabolic Switch to Oxphos in Lymphoma Cells

To determine whether N3a-activated p53 could promote a switch to OxPhos, we used the reduced MitoTracker Red CM-H2XRos to probe the mitochondrial ROS in lymphomas, and samples were analyzed in the Operetta high content screening system (Figure S12). The fluorescence intensity of the probe was significantly increased in all lymphoma cells incubated with 5μM N3a compared to the control, reflecting ROS production, as a by-product of oxidative phosphorylation that contributes to signal transduction.

## 4. Discussion

Currently, there are a few studies of integrated omics analyses in human lymphomas deciphering their pathogenesis [8,38,39,40]. This is the first systems-level study of N3a-induced p53-activation in selected wt-p53 lymphomas of different cell origin. Our data highlight that post-transcriptional mechanisms significantly enhance co-regulation of protein complex subunits beyond transcriptional co-regulation, presenting a fine-tuning of p53-mediated variant and novel insights into post-transcriptional gene regulation in lymphomas. For the first time, we provide indications that N3a evoked G1 arrest by decreasing the G1/S transition-marker DHFR and components of the pre-replication complex/DNA replication machinery. Ikaros, a transcriptional regulator required for the development of all lymphoid lineages, was decreased following p53 activation. Moreover, our data showed p53-mediated induction of cellular senescence and anti-angiogenetic effects, in agreement with previous studies [41,42]. In addition, our data support CDC20′s inhibition as a pathway through which TP53 exerts its anti-lymphoma effects.

In lymphomas, one or more DDR pathways are impaired, possibly leading to an increased dependency on the complementary pathways for survival, promoting pathogenesis and apoptotic resistance [43]. Previous studies have reported N3a-induced activation of DDR occurring in a p53- and MDM2-independent fashion [44,45]. Verma et al. 2010 observed that treatment with 20μm N3a (four times more than our treatment) can cause the formation of double strand DNA strand breaks and initiate a p53-independent DDR in colon cancer cell model characterized by the phosphorylation of H2AX (Ser-139) and p53 (Ser-15), but the DDR was higher in cells exhibiting p53 stabilization. In the same study, they propose that p53 activation by MDM2 inhibitors can result in the decrease of double stranded DNA repair that may suppress HR repair. Our results support these findings, showing significant decrease in HR repair-related proteins RAD51 (R_all), RPA (RPA1: P_all; RPA2: P_ALCL/cHL) and ERCC5 (P_MCL), as well as increase in H2AX that could be related with DNA damage. Both RAD51 and RPA are interacting with p53 inhibiting HR repair, as previously shown [46,47]. Nevertheless, these results show differences among the different lymphoma types examined in this study and should be further studied in the future. Furthermore, Valentine et al. 2011 provided solid evidence showing a secondary role for N3a as a DDR triggering agent, independent of p53 status, and unrelated to its role as an MDM2 antagonist. Even though our results support such a hypothesis in lymphoma cells, further detailed studies should be conducted to provide the detailed mechanisms. Other factors that could attenuate HR repair are the p53 transcription-dependent and independent mechanisms. In the first case, the p53 transcription-dependent cell-cycle arrest mostly in G1 phase decreases HR repair that it is limited to S and G2 phases of the cell cycle [48]. Therefore, HR repair decrease results as a secondary event of the p53-dependent cell cycle arrest. It is not clear yet, which mechanism is the most dominant in the different lymphoma types.

UHRF1, a DDR key-regulator correlated with cancer progression and metastasis, was found in our study significantly decreased in cHL. UHRF1 shows a dual role in DNA replication and epigenetic regulation, where decreased levels of UHRF1 may be responsible for impaired DDR, preventing changes in heterochromatin structure for DNA surveillance and repair. Better understanding of the differential transcriptional and translational p53- and N3a-mediated DDR deregulation, possibly promoting apoptosis, may ultimately lead to combinatorial therapies targeting the remaining critical for survival repair pathways, preventing or delaying relapse.

Moreover, we provide evidence that N3a-treated lymphoma cells exhibit changes in chromatin organization and epigenetic regulation of transcription, in agreement with the reported central role of p53 in chromatin conformation [49,50]. Our data revealed that N3a-treatment may directly lead to increase in the master epigenetic conductor UHRF1 and its privileged partner DNMT1, over-expressed in different cancer types [51] and lymphomas [52], promoting cell growth inhibition. Interestingly, PcG proteins, commonly considered as drivers of lymphomas [53] acting as a recruitment platform for DNA methylation, and NASP, over-expressed in various cancers, were decreased after N3a-treatment. Our data suggest that N3a facilitated the sustained expression of apoptotic genes, reducing anti-apoptotic properties of CTCF and Cohesins, in a context-specific manner. Deciphering the precise mechanisms of chromatin remodeling and its interplay with DDR in wt-p53-deficient lymphomas needs further investigation.

We report that p53 up-regulated antioxidant proteins and the negatively regulated mTOR pathway indicate a N3a-induced metabolic shift in lymphomas, decreasing glycolytic flux and enhancing OxPhos, a metabolic adaptation linked to p53′s role as a metabolic regulator that can be targeted for therapeutic intervention. Our results support enhancement of mitochondrial translation in lymphomas, which may be an additional mechanism for p53-mediated enhancement of OxPhos. Additionally, HCI assessing mitochondrial activity revealed that lymphoma cells undergoing p53 activation via N3a, displayed an increase in MitoTracker Red (CM-H2XRos) that can be accumulated within mitochondria due to the positive charge acquired by ROS oxidation, generated particularly during OxPhos. Overall, our findings agree with previous work, showing a N3a-induced increase in mitochondrial ROS [54] accumulation, the primary stimulus for the mitochondrial death pathway [55]. N3a-activated p53 can inhibit glycolysis by decreasing glycolysis-related enzyme genes shifting metabolism to OxPhos, and/or by increasing TIGAR expression [56], contributing to enhanced mitochondrial ROS accumulation. Moreover, other studies have suggested that reduction in glycolysis may result in the generation of more ROS and sensitize cells to N3a-induced apoptosis [57,58] (Figure S12). Generally, the interplay between p53, OxPhos, and ROS generation in the context of N3a treatment is intricate and can involve multiple mechanisms. It is important to conduct further studies to elucidate the specific molecular pathways and underlying mechanisms responsible for these observations, since the redox cell status regulates cell viability.

Hyper-activated PI3K/mTOR pathway is central in oncogenic processes representing a promising therapeutic target [59], with various studies showing interconnections between p53 and PI3K/mTOR pathway [60]. This is the first comparative omics study in human lymphoma reporting down-regulation of the PI3K/mTOR pathway associated with decreased translation, in accordance to our previous study in MCL [9]. Our results showed up-regulation of Sestrins, β-regulatory AMPK-subunits, and down-regulation of regulatory components of the translational machinery (RPS6KB2/p70S6K-beta, RPS6), supporting an inhibitory effect of p53-re-activation on PI3K/mTOR pathway in lymphomas. Sestrins, previously reported to mediate the p53-effects on AMPK [61], provide evidence for the mechanism underlying the proposed p53-AMPK-mTOR crosstalk in lymphoma. Moreover, activated p53 down-regulated the scaffold protein eIF4GI that interacts with eiF4B required for the binding of mRNA to ribosome, inhibiting protein synthesis downstream of mTOR [62]. Activated p53 mediated its inhibitory effect on mTORC1 also via activated PHLDA3 (negative regulator of AKT) [61,63]. OSMR suppresses proliferation in primary central nervous system lymphoma [64]. Our observation supports a previous report, where p53 was induced by OSM and both converged to apoptosis [65]. Furthermore, besides EIF4B and RPS6KB2/p70S6K-beta, we demonstrate CCNE2, COL6A3, and CCND1 as the most significant down-regulated proteins. Immunoblot data confirmed down-regulation of critical mTOR pathway proteins following p53-activation in cHL and ALCL cells, expanding on our previous studies in MCL. Therefore, the p53-AMPK-mTOR crosstalk can be targeted by combined inhibition of MDM2 and mTOR resulting in enhanced synergistic effects.

Autophagy in lymphoma may act both as a tumor suppressor or cell survival and therapeutic-resistance mechanism [66]. P53, depending on its subcellular location and the stress stimulus, can either inhibit or activate autophagy [67], while multiple cellular stressors, e.g., inhibition of HSP70/mTOR [68,69], can stimulate autophagy. In agreement with previous reports [70,71], our results indicated N3a-mediated induction of autophagy in lymphoma cells, implicating p53-targets DRAM and PRKAB1. Since mTOR is a negative regulator of autophagy and our findings showed N3a-induced down-regulation of the mTOR pathway, autophagy induction in N3a-treated lymphoma cells may be mediated, partly via down-regulated PI3K/mTOR pathway. Moreover, TEM observation investigating possible activation of autophagic flux during apoptosis-induction after N3a treatment, recorded many autophagic vacuoles, such as autophagosomes and autolysosomes in treated lymphoma cells, but scarcely in control cells, along with apoptotic features (chromatin condensation, apoptotic bodies). Accumulation of autophagosomes evidenced by enhanced conversion of an LC3-I fraction to LC3-II expression, along with unchanged or slightly decreased LC3-I expression, was also observed by immunoblot in N3a-treated lymphoma cells [72]. The conversion of a LC3-I fraction to LC3-II is essential for the initiation of the autophagosome formation, while LC3-II abundance correlates with their number. Additionally, activated p53 may support metabolic shift to OxPhos by inhibiting glycolysis, leading to enhanced production of ROS levels, subsequently promoting autophagy [73]. An explanation for this and all related phenomena will certainly require future complementary experimental studies examining the mechanism for the interplay between OxPhos, ROS, and autophagy after N3a in HL, MCL, and ALCL cells.

P53 activation resulted in down-regulation of many components of the HSR, mainly the HSP90/HSP70 family members, frequently over-expressed in cHL/ALCL [6]. HSR halts general protein expression and induces elevated HSPs that correlate with lymphoma pathogenesis and poor prognosis [74,75]. HSPs are often inhibited by p53 or activated upon loss of p53-function in many malignancies [76]. In the present study N3a strongly down-regulated several HSR-components. This is the first omics study reporting a reduced HSR in human lymphoma. HSF1, shown to promote cell growth in human cancer [77] was found down-regulated in cHL after N3a-treatment. HSF1 loss suppresses lymphoma development in p53-deficient mice [78], while HSF1-modulated p53 transcriptional response due to DNA damage depended on the cellular context [79].

Over-expressed HSP90 ensures the function of several proliferation and survival-related oncoproteins, making lymphoma cells depend on its activity. In our study, HSP90 was marginally down-regulated in all N3a-treated cell lines. Furthermore, many co-chaperones, aberrantly expressed in hematologic malignancies enhancing or inhibiting HSP90′s ATPase activity were also down-regulated, proposing a rather N3a-inhibitory effect on HSP90 activity in lymphomas. HSP70, showing reduced levels of most family members, over-expressed in transgenic mouse models promotes lymphomagenesis [74], while its inhibition results in the opposite direction [68]. Moreover, N3a-induced apoptosis may be associated with repressed stress-inducible and ATP-independent HSP27, supporting previous observations of HSP27-inhibitory role in mitochondrial apoptotic signaling [80]. Lastly, p53-activation [81] and HSP90/HSP27-inhibition promoting their extracellular export [82], further explains their decreased levels. Hence, HSR suppression associated with HSP70/HSP90 down-regulation plays an important role in the induction and/or maintenance of N3a-induced apoptosis in lymphoma. These findings provide the biologic rationale for synergistic effects of combined 17-AAG/N3a treatment in lymphoma cells shown here and in our previous studies [83,84,85].

Finally, in our Western blot data interesting proteoforms were detected that should be further studied in future studies. One protein that showed interesting proteoforms is MDM2 that was detected with at least two main isoforms that agree with previous studies in other cell types corresponding to p75 and p90 MDM2 isoforms (Figure S10A) [86]. Cheng et al. 2007 have shown that in human cells the p90 MDM2 isoform is produced preferentially during p53 activation of the MDM2 P2 promoter and it participates in the p53/MDM2 and MDM2/TSG101 feedback control loops. In our results, we detected different patterns in the different lymphoma types examined regarding the relative abundance of the MDM2 isoforms, indicating different mechanisms and effects of MDM2 in these lymphoma types. Further experiments are required to decipher the role of MDM2 in these lymphoma types.

## 5. Conclusions

Our study provides a global profiling of transcriptome and proteome after p53 activation in three selected types of lymphoma that revealed many common and substantially different, cell type-specific effects. Our findings significantly extend our understanding of the potential role master transcription factor wt-p53 holds at a systems biology level, providing the rationale for the design of biologically more relevant therapeutic strategies for lymphoma patients. In addition, our results provide valuable insights into the global impact N3a has in lymphoma subtypes, elucidating its distinct, differential effects on specific proteins and pathways.

## Data Availability

Excel files containing the analyzed data are provided in Supplementary Materials. The mass spectrometry proteomics data have been deposited to the ProteomeXchange Consortium (http://proteomecentral.proteomexchange.org (accessed on 22 March 2018)) via the PRIDE partner repository [96] with the dataset identifier PXD009290.

## Supplementary Materials

The following supporting information can be downloaded at: https://www.mdpi.com/article/10.3390/cancers15153903/s1, Figure S1: Overview of the deregulated omics profiling of N3a-affected lymphoma cells, 2023_Cancers_Supplemental Information_Psatha, 2023_Cancers_Supplemental_Uncropped_raw WBs, Figure S2: Common deregulated mRNAs in three lymphoma types, Figure S3: GO terms and KEGG pathway enrichment analysis of significantly deregulated genes in N3a-treated lymphoma cells in both omics analyses, Figure S4: N3a-induced mRNA and protein regulation in cell-cycle, apoptosis and DNA-repair pathways in the three lymphomas, Figure S5: GO terms and KEGG pathways enrichment in unique deregulated mRNAs and proteins in each lymphoma type, Figure S6: Effect ofN3a-treated cHL, MCL and ALK + ALCL cells in glucose metabolism and OxPhos, Figure S7: N3a-induced mRNA and protein regulation of mTOR, PI3K/AKT and HSPs pathways in the three lymphomas, Figure S8: N3a in combination with HSP90-inhibitor 17-17-AAG enhanced its cytotoxicity effect compared to N3a treatment alone in lymphoma cells, Figure S9: Inhibition of the PI3K/mTOR pathway enhanced the cytotoxicity of N3a in lymphoma cells, Figure S10: Cell-testing; activation of the p53-pathway in cHL, MCL and ALK + ALCL cells before and after treatment with N3a, Figure S11: Validation of selected proteins in ALCL, HL and MCL human lymphoma cells, Figure S12: Transmission Electron Microscopy micrographs, Figure S13: N3a-treated lymphoma cells demonstrate a ROS enhancement supporting OxPhos activity, Table S1: Properties of the lymphoma cell lines used and synergy determination, Table S2: Total omics, common and unique datasets in ALCL, cHL and MCL cell lines, Table S3: Functional and pathway analysis of the three lymphoma cell lines of our study, Table S4: Peptide Report for the samples of JMP1 cell line, Table S5: Peptide Report for the samples of MDAV cell line, Table S6: Peptide Report for the samples of SUPM2 cell line, Table S7: Protein Report for the samples of JMP1 cell line, Table S8: Protein Report for the samples of MDAV cell line, Table S9: Protein Report for the samples of SUPM2 cell line, Table S10: PeptideShaker Export For Progenesis JMP-1, Table S11: PeptideShaker Export For Progenesis MDAV, Table S12: PeptideShaker Export For Progenesis SUPM2, Table S13: Band intensity and ratio calculated from all western blots showed in this study. (References [87,88,89,90,91,92,93,94,95] are cited in the Supplementary Materials).
