# Supplementary material for: Interruption of p53-MDM2 Interaction by Nutlin-3a in Human Lymphoma Cell Models Initiates a Cell-Dependent Global Effect on Transcriptome and Proteome Level
Source: Cancers (Basel). 2023 Jul 31;15(15):3903. doi: 10.3390/cancers15153903 (PMC10417430; doi:10.3390/cancers15153903)
Supplement: Supplementary file 1 [file cancers-15-03903-s001.zip › 2023_Cancers_Supplemental Information_Psatha Revised.pdf]

Supplementary Materials

Supplementary Materials and Methods

Isotope Coded Protein Labeling (ICPL), protein fractionation by SDS-PAGE and in gel tryptic digestion

Isotopic labeling of proteins was conducted as previously described [3]. In short, equal amounts of six protein samples (from un-treated and treated cells) obtained from the three lymphoma cell lines of interest were labeled with ^{12}C-6-nicotinoyl-N-hydroxy-succinimide (^{12}C-Nic-NHS) and^{13}C-Nic-NHS, for un-treated and treated cells, respectively. The protein mixtures in concentrated samples were combined pair wise and were dissolved in NuPAGE buffer for SDS-PAGE fractionation. They were loaded on a NuPAGE® NovexTris-Acetate Gel using an XCellSureLock™ Mini-Cell unit, according to the manufacturer's instructions. Electrophoresis was carried out at 110-125 mA/gel and 200V limit setting, for 35 min. The gel was stained with Coomassie [4]. Each gel-lane was excised into 27–31 slices, in accordance with the number of protein bands revealed. Each slice was cut into smaller pieces of circa 1 mm^3 prior to digestion.The gel slices excised from the SDS-PAGE were in-gel tryptic digested as previously described [5]. Peptides were extracted from gel by the successive addition of ultrapure water, 50% ACN, and 50% ACN with 0.1% TFA, dried in a speed-vacuum centrifuge and dissolved in 5% FA in ultra-pure water solution. All samples were desalted by home-madecolumns packed with RP-C18 extraction disks (Empore) and eluted stepwise with 80% MeOH and 5% FA. All elution fractions were collected, speed-vacuum centrifuged and diluted in 5% FA for further MS analysis.

Filter Aided Sample Preparation (FASP) and in-solution tryptic digestion

Gel-free sample preparation and proteolysis were performed using filter aided sample preparation (FASP) [6] protocol with minor changes. Briefly, cell lysates corresponding to 150 µg of protein were reduced by addition of 10mM DTT at 56°C for 30min followed by alkylation of free thiol groups with 30mM IAA at room temperature (RT) in the dark for 30min. Subsequently, the lysates were diluted up to 300µL with freshly prepared 8M urea-100mM Tris-HCl (pH 8.5) [7] and placed on a Microsep centrifugal device (30KDa, Millipore). The devices were centrifuged at 13,500g at room temperature (RT) for 20min. All the following centrifugation steps were performed under similar conditions. To eliminate residual SDS, three washing steps were carried out using 100µL of 8M urea-100mMTris-HCl (pH 8.5) and finally for the buffer exchange, the devices were washed thrice with 100µL of 50mM TEAB (pH 8.5). To the concentrated proteins, 100µL of digestion buffer comprising, sequencing grade trypsin (1:25 w/w, enzyme to protein), 0.2M GuHCl, 2mM CaCl2 in 50mM TEAB (pH 8.5) were added and incubated at 37°C for 14hrs. The generated tryptic peptides were recovered by centrifugation followed by two consecutive washing steps with 50µL of 50mM TEAB (pH 8.5) and50µL of ultra-pure water. Finally, the peptides were acidified to pH <3 using 10% TFA (v/v) and the digests were quality controlled as described previously [8].

Mass spectrometric analysis by nLC ESI-MS/MS

Protein identification and relative quantitation by nLC-ESI-MS/MS was done on three different systems: an Esquire HCT ion trap (Bruker Daltonics) coupled to a Ultimate 3000 LC-system (Dionex-LC Packings); a LTQ-Orbitrap XL coupled to an Easy nLC (Thermo Scientific); and an Orbitrap Fusion coupled to an Ultimate 3000 nano RSLC system (Thermo Scientific). The sample preparation and the LC separation in the first two systems were performed as previously described [9]with minor modifications.

Briefly, the tryptic peptide mixtures were separated on a reversed-phase column (ReprosilPur C18 AQ, particle size = 3µm, pore size = 120Å (Dr. Maisch), fused silica emitters 100mm long with a 75µm internal diameter (Thermo Scientific), packed in-house, using a pressurized (35 to 40 bars of helium) packing bomb (Loader kit SP035, Proxeon). The nLC flow rate was 300nl min−1.Tryptic peptides were separated and eluted in a linear water-acetonitrile gradient and injected into the mass spectrometer as previously [9, 10]. In the third system, peptides were pre-concentrated on a 75µm x 2cm C18 trapping column for 5min using 0.1% TFA (v/v) with a flow rate of 20µL/min followed by separation on a 75µm x 50cm C18 main column (both Acclaim Pepmapnanoviper, Thermo Scientific) with a 120min LC gradient ranging from 3-42% of 84% ACN in 0.1% FA (v/v) at a flow rate of 250 nL/min. The Orbitrap Fusion MS was operated in a data dependent acquisition mode (OT-Q-CID-IT). MS survey scans were acquired in the Orbitrap (OT) from 300 to 2,000m/z at a resolution of 60,000 and for the MS/MS, precursor isolation at 1.6m/z was performed by the quadrupole (Q). Fragmentation of twenty most intense ions by collision induced dissociation (CID) with normalized collision energy of 35% and rapid scan MS analysis were carried out in the ion trap (IT). The dynamic exclusion duration was set to 15s with 10ppm tolerance around the selected precursor and its isotopes. The AGC target values were set to $4.0x10^5$ and $1.0x10^4$ and maximum injection times were 50ms and 35ms for MS and MS/MS scans, respectively.

Mass spectrometric data processing, protein identification and relative quantitation

The nLC-MS/MS raw data were loaded in Proteome Discoverer 1.3.0.339 (Thermo Scientific) and run using Mascot 2.3.02 (Matrix Science, London, UK) search algorithm against the Human theoretical proteome (Last modified July 9, 2014. Version 153) containing 140,330 entries [11] and a list of common contaminants [12]. For

protein identification, the following search parameters were used: precursor error tolerance = 10ppm, fragment ion tolerance = 0.5Da, trypsin full specificity, maximum number of missed cleavages = 2 (in label free) or 3 (in ICPL), and the following as dynamic modifications: Acetyl (N-terminal), Oxidation (M), ICPL (N-Term)and ICPL (K) (in ICPL experiment). Carbamidomethylation was set as a fixed modification on every cysteine (C) in all samples. Arg-C was selected as the enzyme for the MASCOT database search in ICPL experiments. Protein identification and quantification was achieved using peptides with a Mascot score of >20. The ratios of heavy to light ICPL labeled peptides were used for the relative protein quantitation. For statistical evaluation of the data obtained, the posterior error probability and false discovery rate were used. The false discovery rate was determined by searching a reverse database. A false discovery rate of 0.1% for proteins and peptides was required. Protein relative expression ratios were based on the peak area ratios of the peptides from the same protein identified in different samples. Fold changes in protein expression greater than 1.3 or less than -1.3 were determined to indicate significant proteins. Label free quantification was performed using the Progenesis LC-MS software from Nonlinear Dynamics (Newcastle upon Tyne, U.K.) version 4.1. The triplicate measurements of cell lines that were treated with N3a were compared to the corresponding treated/untreated triplicates separately. MS data processing including alignment of raw data, selection of the reference LC-MS run and peak picking was done automatically by Progenesis. The features within retention time and m/z windows from 0-130 min and 300-2000 m/z, with charge states +2, +3, and +4 were considered for peptide statistics, and analysis of variance (ANOVA). The MS/MS spectra were exported as peak lists, searched against the Human theoretical proteome using Mascot 2.4 (Matrix Science), OMSSA 2.1.9, and X!Tandem Jackhammer (2013.06.15) with the help of

searchGUI 1.14.4 [13] with the parameters described above. For combining the peptide and protein identifications obtained from the three search algorithms, we used our PeptideShaker software 0.28.0 [14]. The combined search results were filtered at a false discovery rate (FDR) of 1 % and were exported using the advanced PeptideShaker features that allow direct re-import of the quality-controlled data into Progenesis. Only proteins that were quantified with at least two unique peptides were exported. Finally, for each protein, the average of the normalized abundances (obtained from Progenesis) from the triplicate analyses was calculated to determine the ratios between the controls/untreated and the respective N3a treated samples.The technical and biological variation of the applied quantitation procedure was evaluated by four independent experiments. For proteins that could be quantified in two or more of the four experiments, the standard deviation was computed as percentage among the different protein regulation factors.

Functional and pathway analysis of deregulated proteins

Functional and pathway enrichment analysis of both transcriptomics and proteomics data was performed using Perseus (1.5.2.6) as part of the MaxQuant Software Package [15]. Categorical annotation was supplied by Gene Ontology (GO) biological process (BP), molecular function (MF) and cellular component (CC), DAVID (Database for Annotation, Visualization and Integrated Discovery), and KEGG pathway database [16, 17]. All annotation was extracted from the UniProt database. Significance was tested by a t-test followed by a false discovery rate correction in the Benjamini-Hochberg procedure (FDR cut off 0.02). Expression values for significant enriched annotations were summarized in an annotation score from -1 to 1 to represent levels of up- or down-regulation.GO terms were selected for further analysis and interpretation using the following criteria for significance: gene count of two and Benjamini-

Hochberg adjusted p-value ≤ 0.05, fold change ≥2. The most biologically significant genes were the ones with the best "enrichment" and "count of genes" values.To obtain a better overview of the direct and indirect interactions between the deregulated genes in the three lymphoma groups, a gene-interaction-network was generated from STRING using KEGG pathway database and UniProt database search [18].

Supplemental Figure legend

Supplemental Figures

FigS1: **Overview of the deregulated omics profiling of N3a-affected lymphoma cells. A:** Venn diagrams corresponding to the identified (left) and the deregulated (right) proteomes in classical Hodgkin lymphoma (cHL), mantle cell lymphoma (MCL) and anaplastic large cell lymphoma (ALCL), showing a strong common signature and a significantly smaller one after N3a-treament, respectively. **B:** Venn diagrams show the overall number of deregulated candidates (mRNA/proteins) in both omics datasets (upper panel) and in each lymphoma-subtype studied (lower panel). The intersections represent the number of commonly identified/quantified candidates irrespective of the direction of their deregulation. Only log2-fold expression changes cut-off of ≥±2 (mRNA; R) and ≥±1.2 (protein; P) levels were considered, representing the number of unique and overlapping candidates in the two omics datasets.

Figure S2: Common deregulated mRNAs in three lymphoma types:

Heat-map and bar-plot image display mRNA expression profile differences for the 28 deregulated genes in the transcriptomic dataset, in each lymphoma subtype of our study. STRING was used for the representation of the interactions networks (apoptosis, cell cycle, DNA repair, signaling, metabolism, etc) involving the common deregulated genes in all lymphoma cells. Heatmaps and bar-plot images have up- (red) or down- (green) regulation color coded for each gene (row) and lymphoma subtype (column),

whereas intensity is proportional to abundance. In the interaction networks, nodes indicate genes and edges indicate the interactions between them, while color depicts regulation (red: increase; green: decrease).

Figure S3: GO terms and KEGG pathway enrichment analysis of significantly deregulated genes in N3a-treated lymphoma cells in both omics analyses: The four sets of heatmaps in each panel show the GO term enrichment (under BP **(A)**, CC **(B)**, MF **(C)** classifications) and pathway analysis **(D)** of selected differentially regulated mRNAs (upper panel) and proteins (lower panel) of our study, based on data from our omics approaches. GO enrichment analysis was done using Gene Ontology Consortium (GOC). Pathways information was based on the Kyoto Encyclopedia of Genes and Genomes (KEGG) pathways database for human. Deregulated mRNAs/proteins were ranked according to GO-terms and KEGG-profiling pathway clustering across ALCL, cHL, MCL cells (left heat-map in each set) and according to their abundance levels in these same GO-terms and pathways clusters in ALCL/HL/MCL (right set of heat-maps in each set), providing a characteristic signature to each lymphoma subtype (FDR-adjusted,–log10: adjusted P-value < 0.05). Color codes same as in figure S3.

Figure S4: N3a-induced mRNA and protein regulation in cell-cycle, apoptosis and DNA-repair pathways in the three lymphomas: Heat-map images depict an overall (mostly common) pattern of differential expression in mRNA and protein level for the cell-cycle (**A**), apoptosis (**B**) and DNA-repair (**C**) related-genes that are deregulated in both omics analyses, corresponding to the three lymphoma cell lines of our study. Color codes same as in figure S3.

Figure S5: GO terms and KEGG pathways enrichment in unique deregulated mRNAs and proteins in each lymphoma type: Top significant GO-term

enrichment(BP (A), MF terms (B), CC (C)) and KEGG pathways categories(D), involving the unique deregulated proteins in ALCL, cHL and MCL using GOC and DAVID (FDR-adjusted,–log10: adjusted P-value < 0.05). Color codes same as in figure S3.

Figure S6: Effect ofN3a-treated cHL, MCL and ALK+ ALCL cells in glucose metabolism and OxPhos: Venn diagrams (upper left panel) of the overlapping (deregulated) proteins involved in glucose metabolism and OxPhos highlighting the cell-specific effect of N3ain the different lymphomas proteome.Bar-plotdiagram (lower left panel) shows the up-regulated (red) and down-regulated (green) proteins involved in the two metabolic pathways after N3a in cHL, MCL and ALCLs. Right panel: STRING visualization of cross-talks between protein-protein interaction networks of signaling pathways (JAK/STAT, PI3K/AKT/mTOR, NF-kB), glucose metabolism and OxPhos after 24hrs of N3a-treatment. Nodes indicate proteins and edges indicate the interactions between them. Table (lower left side of this panel) lists color and line codes.

Figure S7: N3a-induced mRNA and protein regulation of mTOR, PI3K/AKT and HSPs pathways in the three lymphomas: Heat-map depicting the pattern of differential expression in mRNA and protein level for the mTOR, PI3K/AKT and HSPs signaling pathways corresponding to the three lymphoma cell lines of our study.

Figure S8: N3a in combination with HSP90-inhibitor 17-17-AAG enhanced its cytotoxicity effect compared to N3a treatment alone in lymphoma cells: Effect of N3a and 17-AAG in cHL, MCL and ALCL cell lines. Indicated concentrations of N3a (0-6μM) and 17-AAG (0-1μM), were assayed alone or combined in wt-p53-lymphoma cells. DMSO was used as a vehicle control. Each value is the mean of triplicate

experiments ± standard error of the mean.Viability assay and proliferation assays demonstrated a significant dose-dependent enhanced cytotoxicity of N3a when combined with 17-AAG in both 24hrs and 48hrs, with SUP-M2 being the most sensitized cell line, followed by MDA-V. **(C)**. D-F: Dose–effect curves for N3a and 17-AAG at different combination doses in cHL**(D)**, MCL**(E)** and ALCL**(F)** cell lines after 48hrs were obtained using the CalcuSyn software and the multiple drug-effect equation of Chou [2].

Figure S9: Inhibition of the PI3K/mTOR pathway enhanced the cytotoxicity of N3a in lymphoma cells: Indicated concentrations of N3a (0-3μM), LY294002 (0-50μM) and Rapamycin (0-10nM), were assayed alone or combined inwt-p53- (MDA-V: cHL (A), JMP-1: MCL (B); SUP-M2: ALCL, (C)) and mutant p53 SUDHL-1 (C) lymphoma cell lines. DMSO was used as a vehicle control. Each value reflects the mean of triplicate experiments ± standard error of the mean. N3a's cytotoxicity was significantly enhanced using LY294002 compound, especially in SUP-M2.

Figure S10: Cell-testing; activation of the p53-pathway in cHL, MCL and ALK+ ALCL cells before and after treatment with N3a: A: Expression status of p53 was assessed in cHL, MCL and ALK+ ALCL cell lines after exposure to the MDM2 antagonist (5 μM) or an equivalent amount of DMSO (vehicle control). Whole-cell lysates were then prepared after 24hrs treatment and analysed by WB, resulting in an activated p53 gene product in all three-cell lines. MDM2 protein levels are also presented. b-Actin was used as the housekeeping gene, for protein load and integrity control. **B:** N3a induced cell cycle arrest in HL and NHL cell lines harboring wt*p53*. Cell lines were treated for 24hrs with either N3a (5 μM) or DMSO (vehicle control). Cell-cycle analysis using propidium iodide (PI) staining and flow cytometry confirmed cell-cycle arrest through the stabilization and activation of p53. In agreement with

previous studies, cell cycle profile presented an arrest at G1 phase, as the fraction of cells in S-phase was decreased. **C:** N3a inhibited viability in HL and NHL cell lines with wt-*p53*. Cell viability was assessed using trypan blue exclusion assay, after 24h treatment with either N3a (5 μM) or DMSO (vehicle control). Results were expressed as the mean of three independent viable cell counts i) or their relative numbers ii) compared to DMSO-control. Treatment with N3a resulted in a reduction in the percentage of viable cells. **D:** Effect on apoptosis of N3a on cHL, MCL and ALK+ ALCL cell lines. All three-cell lines were incubated for 24 hrs with either 5 µM N3a or DMSO (vehicle control). Cells were harvested and subjected to Annexin V/PI staining, followed by flow cytometry analysis to evaluate apoptosis. Treated cells demonstrated an increased percentage of Annexin V-positive cells with respect to the DMSO control. Experiments were repeated at least twice, with consistent findings from independent trials.

Figure S11: Validation of selected proteins in ALCL, HL and MCL human lymphoma cells: Selective expression of TIGAR (p53-inducible regulator of glycolysis and apoptosis), IKAROS (a central regulator of lymphocyte differentiation, involved in chromatin remodeling and histone deacetylation), ATG5, ATG4B and LC3 I/II (autophagy process) and p65 (NF-κB subunit, involved in regulation of inflammation, proliferation, and apoptosis) in the HL and NHL cell lines of our study was confirmed by Western blotting. TIGAR, IKAROS, ATG4B, LC3 and p65 protein levels were differentially affected between the different lymphoma cell types, in a dose-dependent manner after treatment with N3a. Association of protein identified in our proteomics analysis with respective pathways is shown in right figure column.

Figure S12: Transmission Electron Microscopy micrographs. Electron micrographs showing representative apoptotic and autophagic features in control (A-C) and N3a-treated (5µM, 24 hrs; D-I) ALK+ALCL (A, D, G), HL (B, E, H) and MCL cells (C, F, I) (magnification is specified in pictures). Apoptosis: Nuclear shrinkage (arrow head); Chromatin condensation (small arrow). Autophagy, formation of autophagic vacuoles: autophagosomes (arrow head); autolysosomes, with degraded vacuolar content (small arrow).

Figure S13: N3a-treated lymphoma cells demonstrate a ROS enhancement supporting OxPhos activity. A high content screening assay using the Operetta platform was employed to determine the effect of N3a on mitochondrial ROS formation. ROS generation was induced in the presence of N3a (5µM) compared to vehicle. The figure shows the mean cell intensity of mitotracker of the live cell population, for each cell line ALCL, HL, MCL. Each dot represents the log 2 mean intensity per cell for a total of 18000, 9000, 12000 cells for ALCL, HL, MCL respectively. The mean intensity for each well is depicted with horizontal black lines. Increased mitochondrial activity was observed in the N3a-treated cells, in all three human lymphoma cell types, suggesting an increased mitochondrial activity on those cells (**** $p<0.0001$). Mitochondrial membrane potential was calculated as the ratio of MitoTracker™ Red CMXRos stain to blue Hoechst-positive results (stained nuclei). Three individual biological experiments were carried out and each of them included three technical replicates. Only live cells were included in the analysis. Mitotracker is a live cell fluorescence dye activated in respiring mitochondria, so cells without mitotracker were excluded, classified as dead.

Supplemental Tables legends

Supplemental Tables

Supplementary Table S1: Properties of the lymphoma cell lines used and synergy determination. This table consists of 2 tabs. The first tab (Table S1A) summarizes details on references and characteristics about the lymphoma cell linesused in the omics study. The second tab (Table S1B) contains information on the ED50, ED75 and ED90 values (the effective doses at which 50%, 75% and 95% of cell killing occurred, respectively) (i) and the combination index values betweenN3a and 17-AAG at different combination dosesin cHL, MCL (ii) and ALCL (iii) cell lines after 48 hours, determined according to Calcusyn software. Data represent the mean of three independent experiments performed in triplicate. A combination index <1 indicates synergy.

Supplementary Table S2: Total omics, common and unique datasets in ALCL, cHL and MCL cell lines: Normalized datasets of total omics (TableS2A), common (Table S2B) and unique (Table S2C-Table S2E) deregulated mRNAs & proteins in three treated and non-treated (control) with N3awt-p53-lymphoma cell lines obtained from transcriptomic (Affymetrix; HG-U133B) and proteomic analyses (label; ICPL and label-free). Total dataset comprises from 5325 genes/proteins, where 673 mRNAs and 3578 proteins were found to be deregulated in the three lymphoma cell lines of our study (N3a/control) (TableS2A). 28 deregulated mRNAs and 141 deregulated proteins overlapped in the two datasets of our analysis (TableS2B). Furthermore, 66 (ALCL), 151 (cHL), 267 (MCL) genes and 461 (ALCL), 726 (cHL), 654 (MCL) proteins were found to be uniquely deregulated in our in vitro system (TableS2C-Table 2E). Probe Set IDs derived from Affymetrix, Uniprot IDs derived from David Conversion Tool and gene/protein names and functional analysis derived from UniProtKB.

Supplementary Table S3: Functional and pathway analysis of the three lymphoma cell lines of our study: Functional and pathway enrichment analyses of the deregulated gene and protein datasets (TableS3A-TableS3D) and of the common (TableS3E-TableS3H) and unique (TableS3I-TableS3L) deregulated genes and proteins in three treated and non-treated (control) with N3awtp53-lymphoma cell lines obtained from transcriptomic (Affymetrix; HG-U133B) and proteomic analyses (label; ICPL and label-free). Gene Ontology and pathway enrichment analyses of the significant differentially regulated mRNAs/proteins (N3a/control) were performed using GOC and the KEGG Pathway feature of DAVID software ver.6.7, respectively. Ontological and pathway analyses assigned associated deregulated mRNAs/proteins to enriched GO and pathway terms, showing coordinated up- or down-regulation of the related categories. Details on the information presented in Table S3A-TableS3L can be found at the bottom of each table.

Supplementary Table S4: Peptide Report for the samples of JMP1 cell line: All the peptides identified by Proteome Discoverer in all JMP1 samples analyzed in Esquire HCT ion trap and LTQ-Orbitrap XL were merged in one file by Scaffold software and extracted in a excel file. The file contains all the information for each peptide identified.

Supplementary Table S5: Peptide Report for the samples of MDAV cell line: All the peptides identified by Proteome Discoverer in all MDAV samples analyzed in Esquire HCT ion trap and LTQ-Orbitrap XL were merged in one file by Scaffold software and extracted in a excel file. The file contains all the information for each peptide identified.

Supplementary Table S6: Peptide Report for the samples of SUPM2 cell line: All the peptides identified by Proteome Discoverer in all SUPM2 samples analyzed in

Esquire HCT ion trap and LTQ-Orbitrap XL were merged in one file by Scaffold software and extracted in a excel file. The file contains all the information for each peptide identified.

Supplementary Table S7: Protein Report for the samples of JMP1 cell line: All the proteins identified by Proteome Discoverer in all JMP1 samples analyzed in Esquire HCT ion trap and LTQ-Orbitrap XL were merged in one file by Scaffold software and extracted in a excel file. The file contains all the information for each protein identified.

Supplementary Table S8: Protein Report for the samples of MDAV cell line: All the proteins identified by Proteome Discoverer in all MDAV samples analyzed in Esquire HCT ion trap and LTQ-Orbitrap XL were merged in one file by Scaffold software and extracted in a excel file. The file contains all the information for each protein identified.

Supplementary Table S9: Protein Report for the samples of SUPM2 cell line: All the proteins identified by Proteome Discoverer in all MDAV samples analyzed in Esquire HCT ion trap and LTQ-Orbitrap XL were merged in one file by Scaffold software and extracted in a excel file. The file contains all the information for each protein identified.

Supplementary Table S10: PeptideShaker Export For Progenesis JMP-1: All the peptides identified and quantified by Progenesis in all JMP1 samples analyzed in Orbitrap Fusion were merged in one file by PeptideShaker software and extracted in a excel file. The file contains all the information for each peptide identified.

Supplementary Table S11: PeptideShaker Export For Progenesis MDAV: All the peptides identified and quantified by Progenesis in all MDAV samples analyzed in

Orbitrap Fusion were merged in one file by PeptideShaker software and extracted in a excel file. The file contains all the information for each peptide identified.

Supplementary Table S12: PeptideShaker Export For Progenesis SUPM2: All the peptides identified and quantified by Progenesis in all SUPM2 samples analyzed in Orbitrap Fusion were merged in one file by PeptideShaker software and extracted in a excel file. The file contains all the information for each peptide identified.

Supplemental text

Enhanced N3a effect on lymphoma cells in combination to Hsp90 inhibition

Treating lymphoma cells for 24h/48h with low concentrations of N3a or 17-AAG was compared to either compound alone or in combination, leading to a dose-dependent appreciable reduction of cell viability and cell growth. After 24h-treatment, 17-AAG enhanced the cytotoxicity and inhibitory effect of N3a, reducing the percentage of viable cells compared to the control group by an additional 18% in HL, 29% in MCL and 34% in ALCL, and cell growth by an additional 23%, 32% and 33%, respectively. 17-AAG-exposure for 48h enhanced even more the cytotoxicity and inhibitory effect of N3a (percentage of viable cells compared to the control decreased up to97% in HL,63% in MCL and 98% in ALCL), decreasing cell viability compared to control up to 97% in HL, 92% in MCL and 98% in ALCL and cell growth from 45%, 16% and 23%, respectively.

In order to qualitatively evaluate whether the combination of N3a with 17-AAG can generate synergistic antiproliferative effects, CI index, a commonly used evaluation method and dose-effect analysis was calculated, in accordance with Chou Talalay method using CalcuSyn [21]. In our study, simultaneous administration of N3a and 17-AAG showed highest synergistic effects on MCL and ALCL cells at the ED50 levels

(CI_{HL}: 0.785, CI_{MCL}: 0.239 and CI_{ALCL}: 0.399, Figure S7, Table S1B). Combination index values indicate synergy (values <1) for N3a plus 17-AAG maintained in a fixed ratio.

Enhanced N3a effect on lymphoma cells in combination to PI3K/mTOR inhibition

Previous publications report simultaneous inhibition of PI3K/mTOR signaling using mTOR inhibitor Rapamycin (Rapa) and/or PI3K inhibitor LY294002 with N3a-p53-activation reinforced induction of p53-mediated apoptosis in CTCL cells [22]. In this regard, we investigated the effect of PI3K/mTOR inhibitors in targeting both mTOR and N3a-induced p53-activation in our HL/MCL/ALCL cells (Fig.7, S8). Lymphoma cells were treated for 24h/48h with low concentrations of N3a, LY294002, Rapa or the combination of N3a/LY294002 and N3a/Rapa, leading to an appreciable reduction of cell viability and cell growth. 24h-treatment of all three cell lines with N3a/LY/Rapa did not show any significant effect (data not shown). However, following 48h application of LY294002 and Rapa, the cytotoxicity and inhibitory effect of N3a was augmented, causing a significant decrease in cell viability, while cell growth was reduced in HL from 72 to 48 and 66%, in MCL from 70 to 42 and 72% and in ALCL from 79 to 9 and 60%, respectively. ALCL cell line SUDHL-1 containing mutant p53 was used as a control, showing no and less sensitivity to N3a, LY294002 and Rapa-induced effect, correspondingly.

References

1. Drakos, E.; Atsaves, V.; Schlette, E.; Li, J.; Papanastasi, I.; Rassidakis, G.Z.; Medeiros, L.J. The therapeutic potential of p53 reactivation by nutlin-3a in ALK+ anaplastic large cell lymphoma with wild-type or mutated p53. *Leukemia* **2009**, *23*, 2290–2299. https://doi.org/10.1038/leu.2009.180.
2. Chou, T.-C. Drug combination studies and their synergy quantification using the Chou-Talalay method. *Cancer Res.* **2010**, *70*, 440–446. https://doi.org/10.1158/0008-5472.can-09-1947.

3. Tebbe, A.; Schmidt, A.; Konstantinidis, K.; Falb, M.; Bisle, B.; Klein, C.; Aivaliotis, M.; Kellermann, J.; Siedler, F.; Pfeiffer, F.; et al. Life-style changes of a halophilic archaeon analyzed by quantitative proteomics. *Proteomics* **2009**, *9*, 3843–3855. https://doi.org/10.1002/pmic.200800944.
4. Candiano, G.; Bruschi, M.; Musante, L.; Santucci, L.; Ghiggeri, G.M.; Carnemolla, B.; Orecchia, P.; Zardi, L.; Righetti, P.G. Blue silver: A very sensitive colloidal Coomassie G-250 staining for proteome analysis. *Electrophoresis* **2004**, *25*, 1327–1333. https://doi.org/10.1002/elps.200305844.
5. Shevchenko, A.; Wilm, M.; Vorm, O.; Mann, M. Mass Spectrometric Sequencing of Proteins from Silver-Stained Polyacrylamide Gels. *Anal. Chem.* **1996**, *68*, 850–858. https://doi.org/10.1021/ac950914h.
6. Wiśniewski, J.R.; Zougman, A.; Nagaraj, N.; Mann, M. Universal sample preparation method for proteome analysis. *Nat. Methods* **2009**, *6*, 359–362. https://doi.org/10.1038/nmeth.1322.
7. Kollipara, L.; Zahedi, R.P. Protein carbamylation: In vivo modification or in vitro artefact? *Proteomics* **2013**, *13*, 941–944.
8. Burkhart, J.M.; Schumbrutzki, C.; Wortelkamp, S.; Sickmann, A.; Zahedi, R.P. Systematic and quantitative comparison of digest efficiency and specificity reveals the impact of trypsin quality on MS-based proteomics. *J. Proteom.* **2012**, *75*, 1454–1462. https://doi.org/10.1016/j.jprot.2011.11.016.
9. Aivaliotis, M.; Gevaert, K.; Falb, M.; Tebbe, A.; Konstantinidis, K.; Bisle, B.; Klein, C.; Martens, L.; Staes, A.; Timmerman, E.; et al. Large-Scale Identification of N-Terminal Peptides in the Halophilic Archaea *Halobacterium salinarum* and *Natronomonas pharaonis*. *J. Proteome Res.* **2007**, *6*, 2195–2204. https://doi.org/10.1021/pr0700347.
10. Aivaliotis, M.; Macek, B.; Gnad, F.; Reichelt, P.; Mann, M.; Oesterhelt, D. Ser/Thr/Tyr Protein Phosphorylation in the Archaeon Halobacterium salinarum—A Representative of the Third Domain of Life. *PLoS ONE* **2009**, *4*, e4777. https://doi.org/10.1371/journal.pone.0004777.
11. UniProt Consortium. The Universal Protein Resource (UniProt) in 2010. *Nucleic Acids Res.* **2010**, *38*, D142–D148.
12. Rappsilber, J.; Ryder, U.; Lamond, A.I.; Mann, M. Large-Scale Proteomic Analysis of the Human Spliceosome. *Genome Res.* **2002**, *12*, 1231–1245. https://doi.org/10.1101/gr.473902.
13. Vaudel, M.; Barsnes, H.; Berven, F.S.; Sickmann, A.; Martens, L. SearchGUI: An open-source graphical user interface for simultaneous OMSSA and X!Tandem searches. *Proteomics* **2011**, *11*, 996–999. https://doi.org/10.1002/pmic.201000595.
14. Vaudel, M.; Burkhart, J.M.; Zahedi, R.; Oveland, E.; Berven, F.S.; Sickmann, A.; Martens, L.; Barsnes, H. PeptideShaker enables reanalysis of MS-derived proteomics data sets. *Nat. Biotechnol.* **2015**, *33*, 22–24. https://doi.org/10.1038/nbt.3109.
15. Cox, J.; Mann, M. 1D and 2D annotation enrichment: A statistical method integrating quantitative proteomics with complementary high-throughput data. *BMC Bioinform.* **2012**, *13*, S12. https://doi.org/10.1186/1471-2105-13-s16-s12.
16. Gene Ontology Consortium. Gene Ontology Consortium: Going forward. *Nucleic Acids Res*. **2015**, *43*, D1049–D1056.
17. Kanehisa, M.; Sato, Y.; Kawashima, M.; Furumichi, M.; Tanabe, M. KEGG as a reference resource for gene and protein annotation. *Nucleic Acids Res.* **2015**, *44*, D457–D462. https://doi.org/10.1093/nar/gkv1070.
18. Franceschini, A.; Szklarczyk, D.; Frankild, S.; Kuhn, M.; Simonovic, M.; Roth, A.; Lin, J.; Minguez, P.; Bork, P.; Von Mering, C.; et al. STRING v9.1: Protein-protein interaction networks, with increased coverage and integration. *Nucleic Acids Res.* **2013**, *41*, D808–D815.
19. Szklarczyk, D.; Morris, J.H.; Cook, H.; Kuhn, M.; Wyder, S.; Simonovic, M.; Santos, A.; Doncheva, N.T.; Roth, A.; Bork, P.; et al. The STRING database in 2017: Quality-controlled protein-protein association networks, made broadly accessible. *Nucleic Acids Res.* **2017**, *45*, D362–D368.
20. Smoot, M.E.; Ono, K.; Ruscheinski, J.; Wang, P.-L.; Ideker, T. Cytoscape 2.8: New features for data integration and network visualization. *Bioinformatics* **2011**, *27*, 431–432. https://doi.org/10.1093/bioinformatics/btq675.
21. Chou, T.-C. Theoretical Basis, Experimental Design, and Computerized Simulation of Synergism and Antagonism in Drug Combination Studies. *Pharmacol. Rev.* **2006**, *58*, 621–681. https://doi.org/10.1124/pr.58.3.10.
22. Manfe, V.; Biskup, E.; Rosbjerg, A.; Kamstrup, M.; Skov, A.G.; Lerche, C.M.; Lauenborg, B.T.; Ødum, N.; Gniadecki, R. miR-122 regulates p53/Akt signalling and the chemotherapy-induced apoptosis in cutaneous T-cell lymphoma. *PLoS ONE* **2012**, *7*, e29541.
