# Supplementary material for: Interruption of p53-MDM2 Interaction by Nutlin-3a in Human Lymphoma Cell Models Initiates a Cell-Dependent Global Effect on Transcriptome and Proteome Level
Source: Cancers (Basel). 2023 Jul 31;15(15):3903. doi: 10.3390/cancers15153903 (PMC10417430; doi:10.3390/cancers15153903)
Supplement: Supplementary file 1 [file cancers-15-03903-s001.zip › Figure S11_WBs_Validation of selected proteins.pdf]

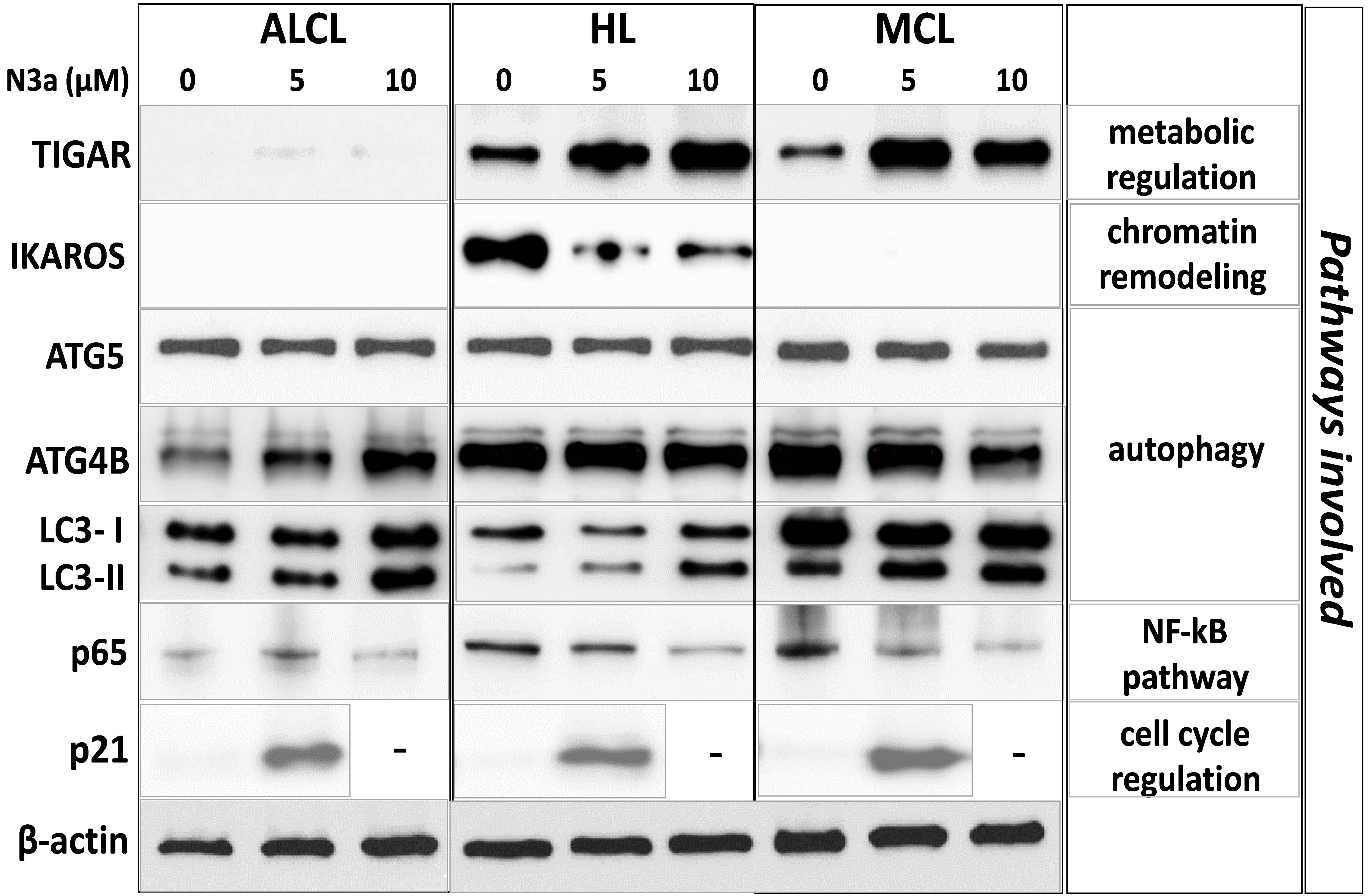

ALCL
HL
MCL
N3a (μM)
0
5
10
0
5
10
0
5
10
TIGAR
IKAROS
ATG5
ATG4B
LC3- I
LC3-II
p65
p21
β-actin
metabolic regulation
chromatin remodeling
autophagy
NF-kB pathway
cell cycle regulation
Pathways involved
