# Supplementary material for: Interruption of p53-MDM2 Interaction by Nutlin-3a in Human Lymphoma Cell Models Initiates a Cell-Dependent Global Effect on Transcriptome and Proteome Level
Source: Cancers (Basel). 2023 Jul 31;15(15):3903. doi: 10.3390/cancers15153903 (PMC10417430; doi:10.3390/cancers15153903)

Figure S12: Electron micrographs showing representative apoptotic and autophagic features in control and N3a-treated cells. Psatha et al. 2023

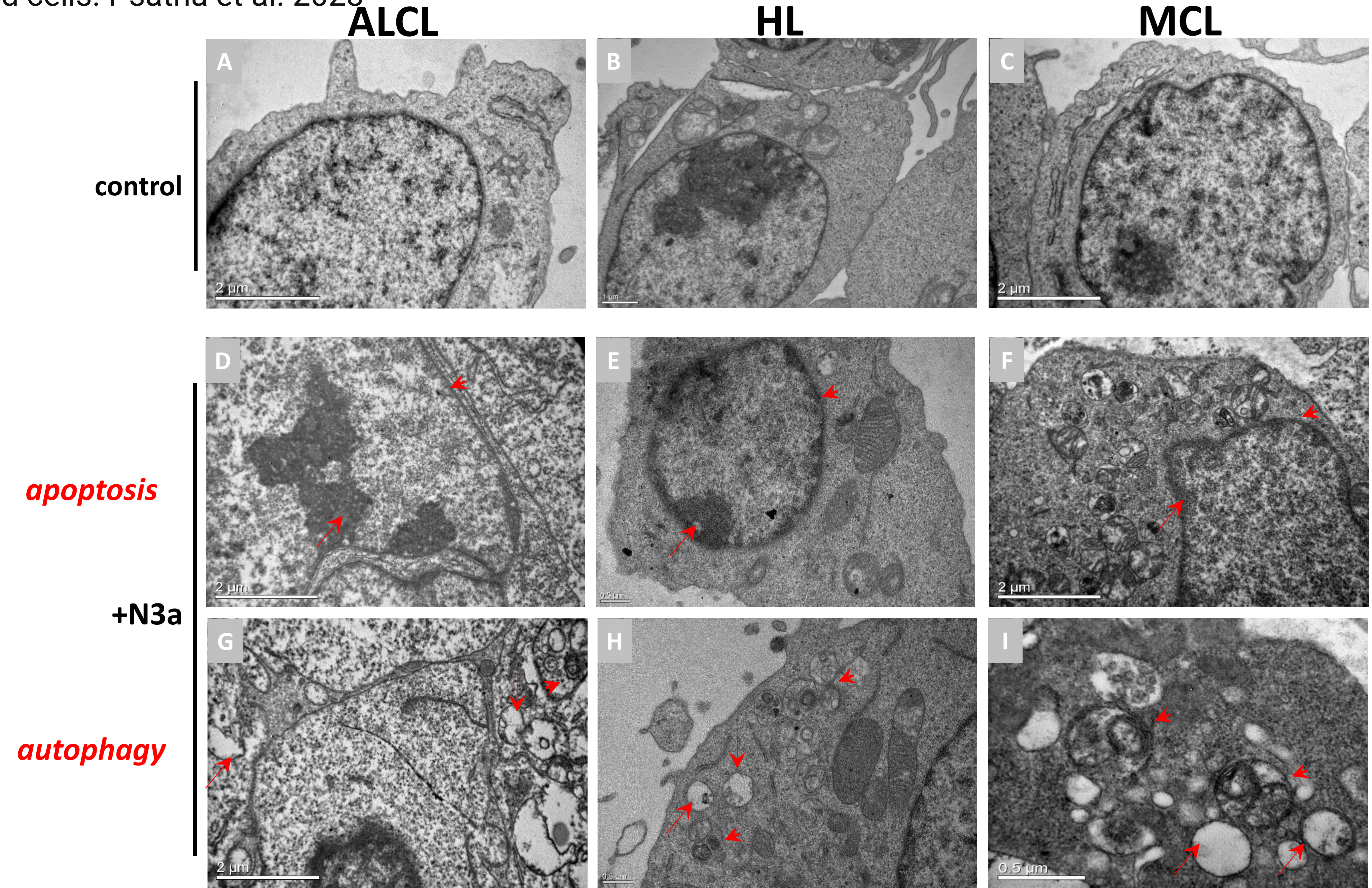

Supplement: Supplementary file 1 [file cancers-15-03903-s001.zip › Figure S12 TEM.pdf]
