# Supplementary material for: Interruption of p53-MDM2 Interaction by Nutlin-3a in Human Lymphoma Cell Models Initiates a Cell-Dependent Global Effect on Transcriptome and Proteome Level
Source: Cancers (Basel). 2023 Jul 31;15(15):3903. doi: 10.3390/cancers15153903 (PMC10417430; doi:10.3390/cancers15153903)
Supplement: Supplementary file 1 [file cancers-15-03903-s001.zip › Graphical Abstract Psatha et al Final.pdf]

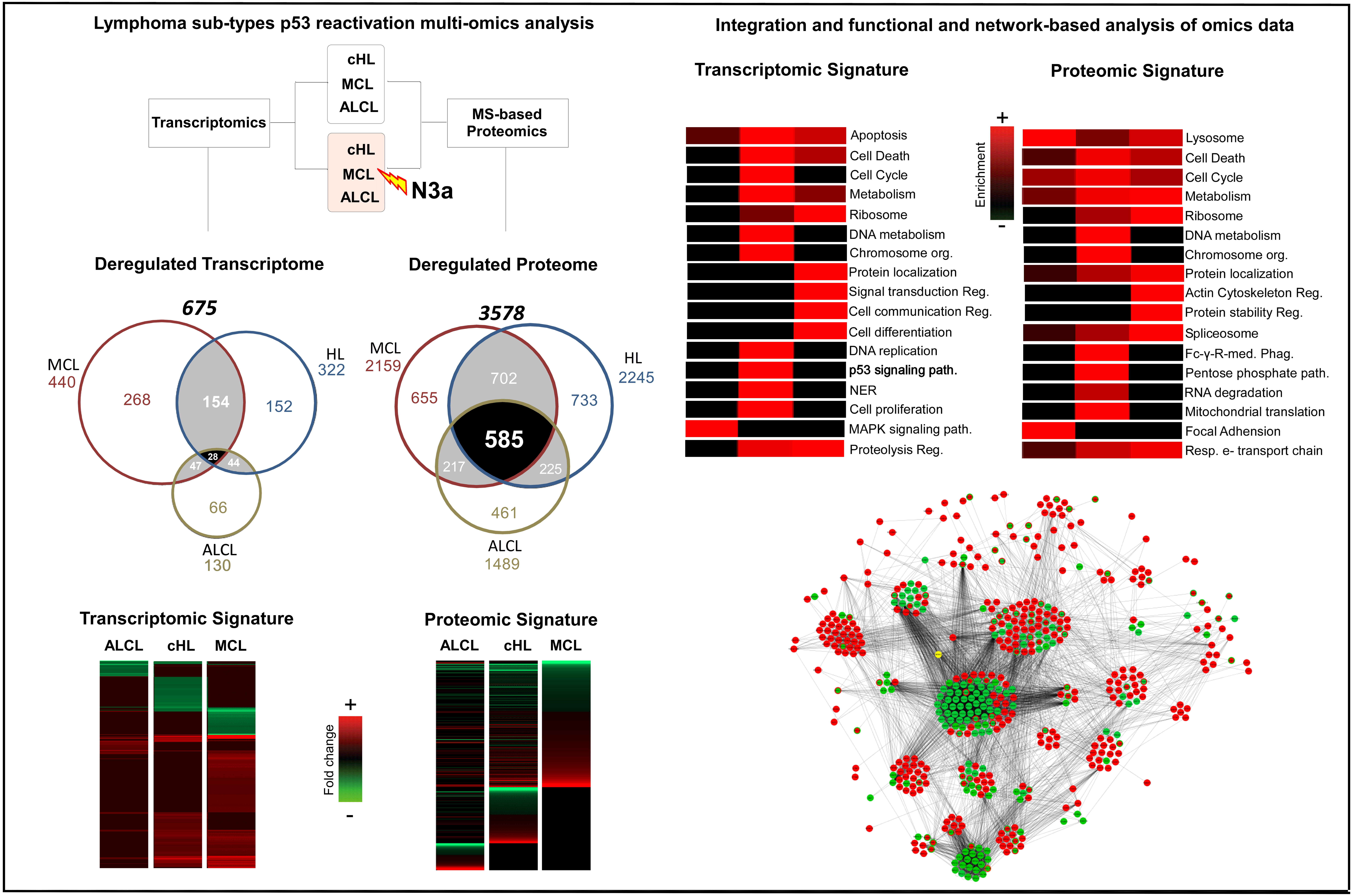

Lymphoma sub-types p53 reactivation multi-omics analysis
Transcriptomics
cHL
MCL
ALCL
cHL
MCL
ALCL
N3a
MS-based Proteomics
Deregulated Transcriptome
675
MCL 440
HL 322
ALCL 130
268
154
152
28
47
44
66
Deregulated Proteome
3578
MCL 2159
HL 2245
ALCL 1489
655
702
733
585
217
225
461
Transcriptomic Signature
ALCL
cHL
MCL
Fold change
Proteomic Signature
ALCL
cHL
MCL
Integration and functional and network-based analysis of omics data
Transcriptomic Signature
Apoptosis
Cell Death
Cell Cycle
Metabolism
Ribosome
DNA metabolism
Chromosome org.
Protein localization
Signal transduction Reg.
Cell communication Reg.
Cell differentiation
DNA replication
p53 signaling path.
NER
Cell proliferation
MAPK signaling path.
Proteolysis Reg.
Enrichment
Proteomic Signature
Lysosome
Cell Death
Cell Cycle
Metabolism
Ribosome
DNA metabolism
Chromosome org.
Protein localization
Actin Cytoskeleton Reg.
Protein stability Reg.
Spliceosome
Fc-γ-R-med. Phag.
Pentose phosphate path.
RNA degradation
Mitochondrial translation
Focal Adhension
Resp. e- transport chain
