# Supplementary material for: Interruption of p53-MDM2 Interaction by Nutlin-3a in Human Lymphoma Cell Models Initiates a Cell-Dependent Global Effect on Transcriptome and Proteome Level
Source: Cancers (Basel). 2023 Jul 31;15(15):3903. doi: 10.3390/cancers15153903 (PMC10417430; doi:10.3390/cancers15153903)

RAW FIGURES WBs

Monitoring N3a’s-effect on p53 & MDM2 in HL/NHL cells

MDM2 in HL/NHL -/+N3a

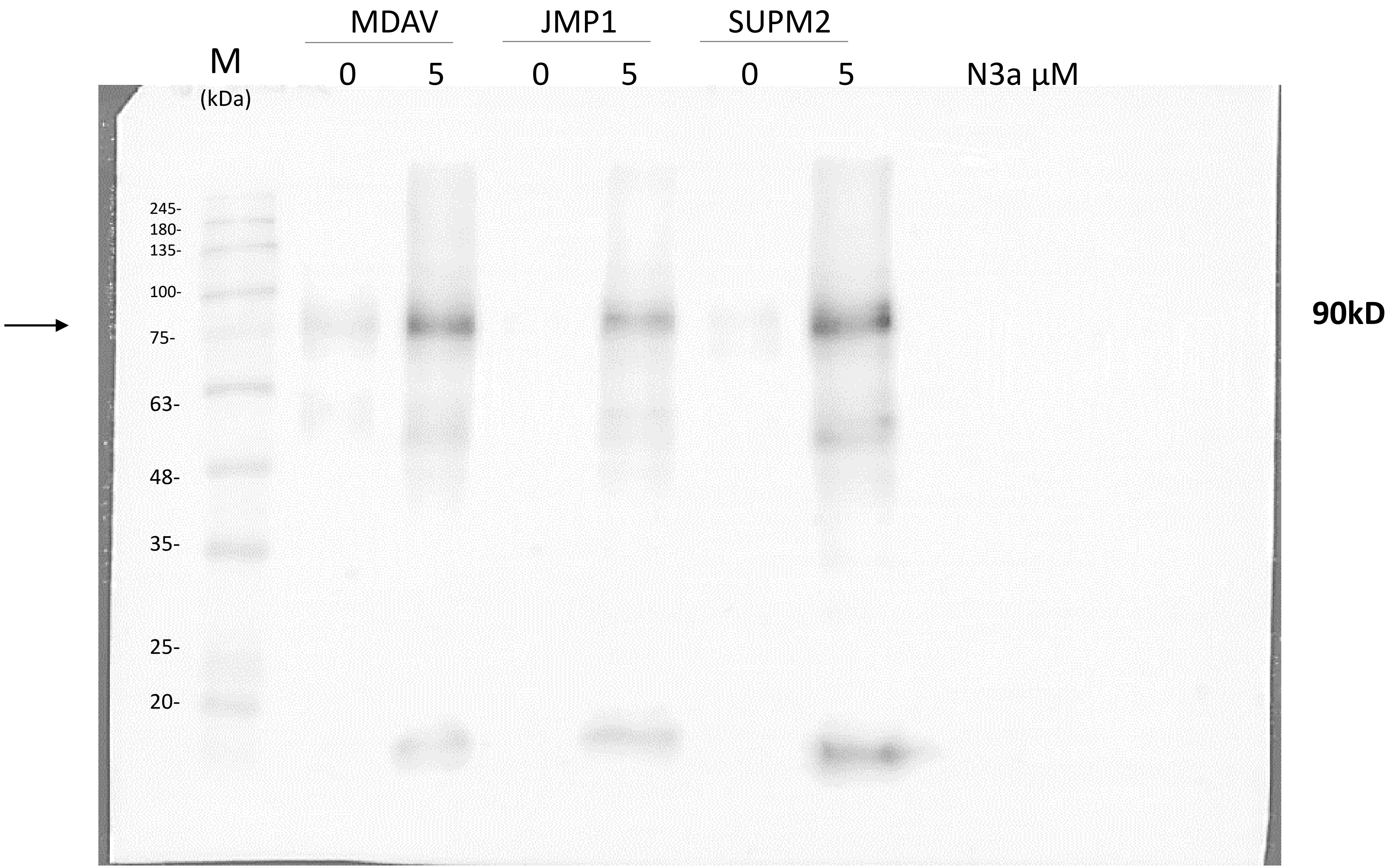

β-actin as a loading control in HL/NHL -/+N3a

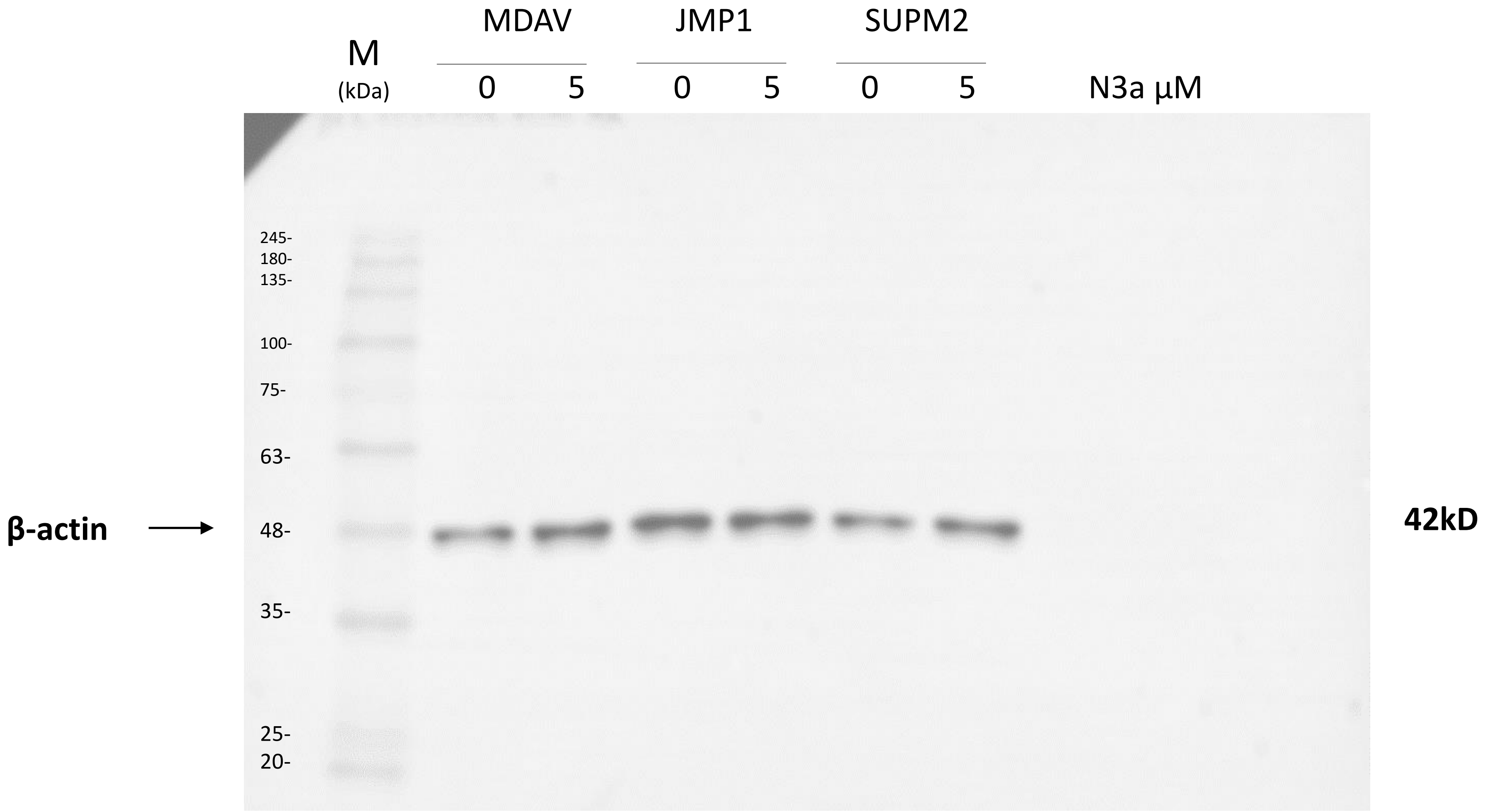

p53 in HL/NHL -/+N3a

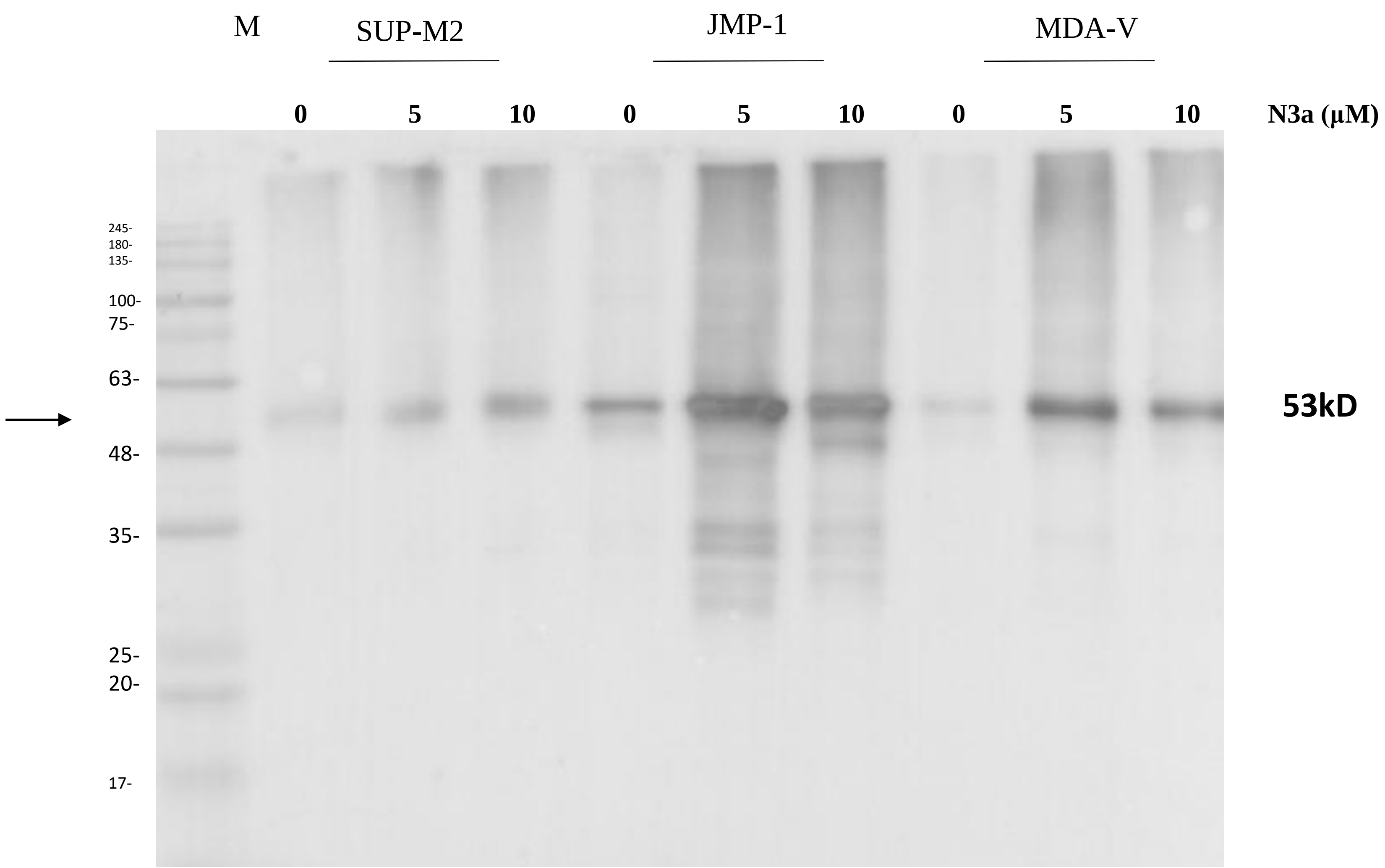

Monitoring N3a's-effect on cell cycle-related protein in HL/NHL cells

p21 in HL/NHL -/+N3a

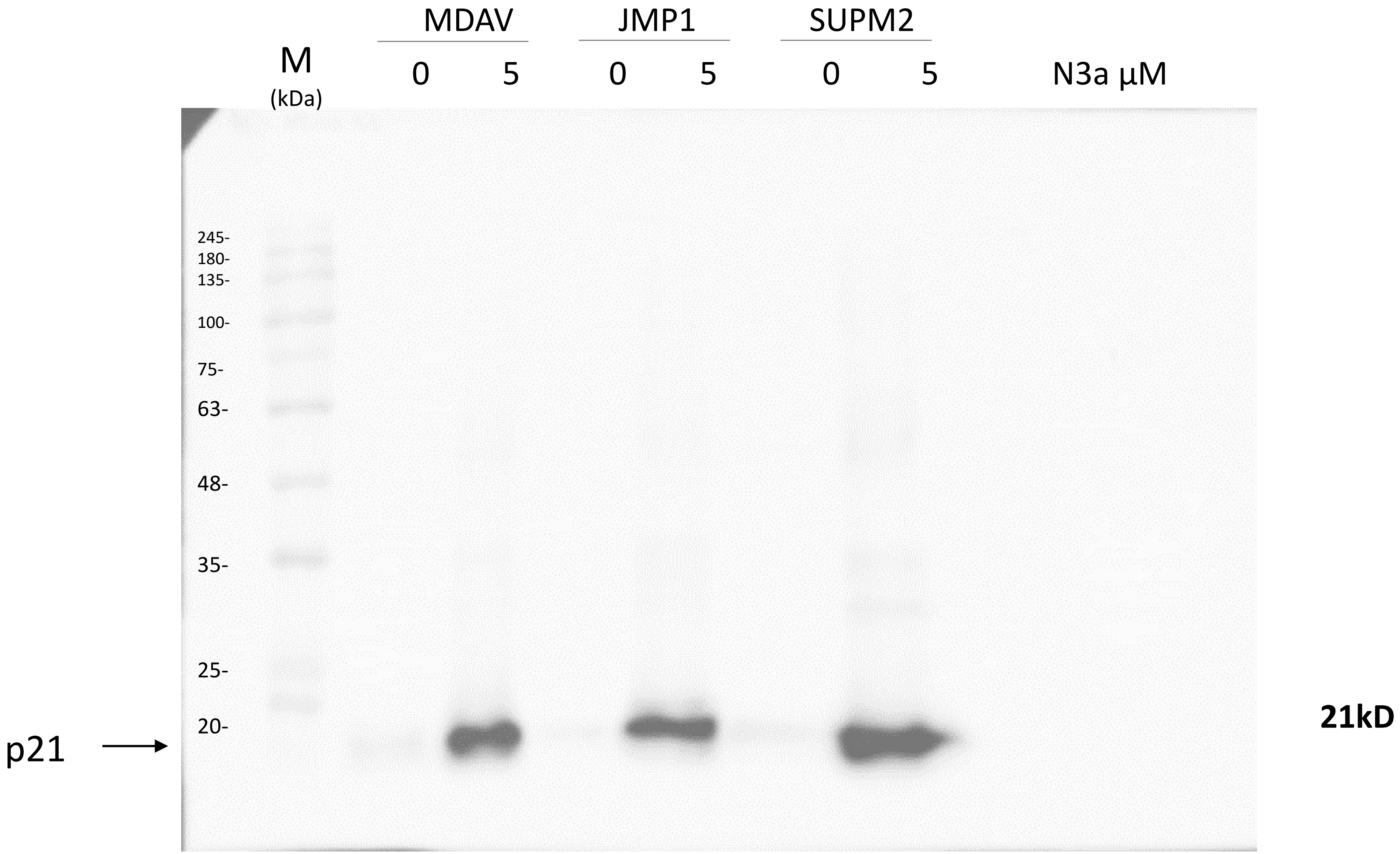

Monitoring N3a's-effect on mTOR-related proteins in HL/NHL cells

mTOR in HL/NHL -/+N3a

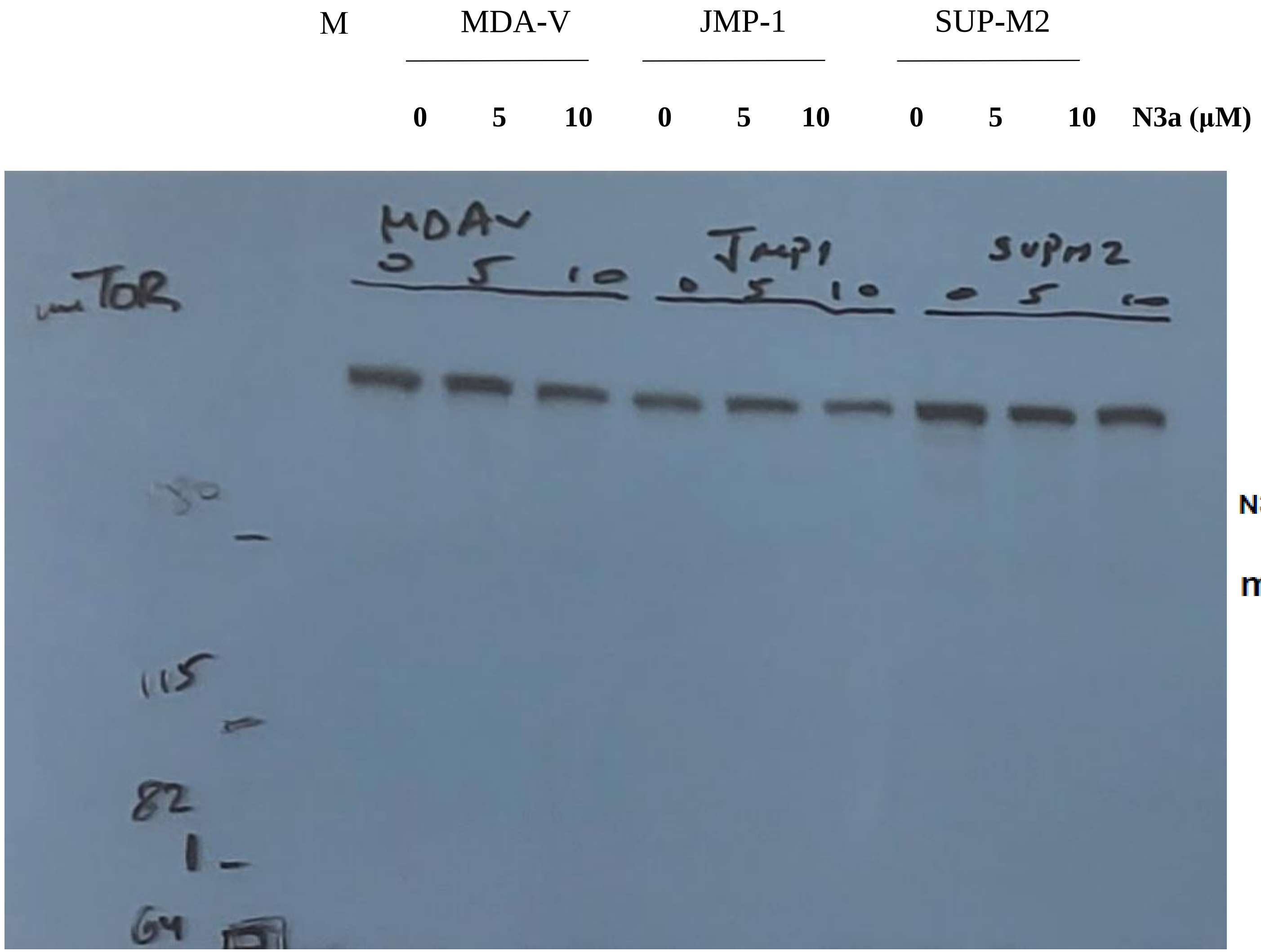

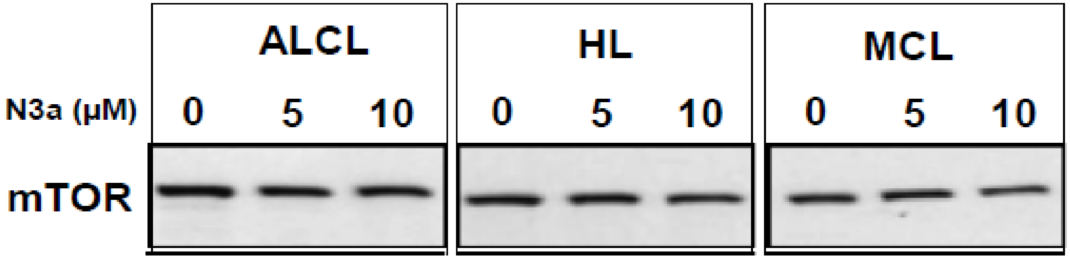

p-mTOR in HL/NHL -/+N3a

M	MDA-V			JMP-1			SUP-M2			
	**0**	**5**	**10**	**0**	**5**	**10**	**0**	**5**	**10**	**N3a (µM)**

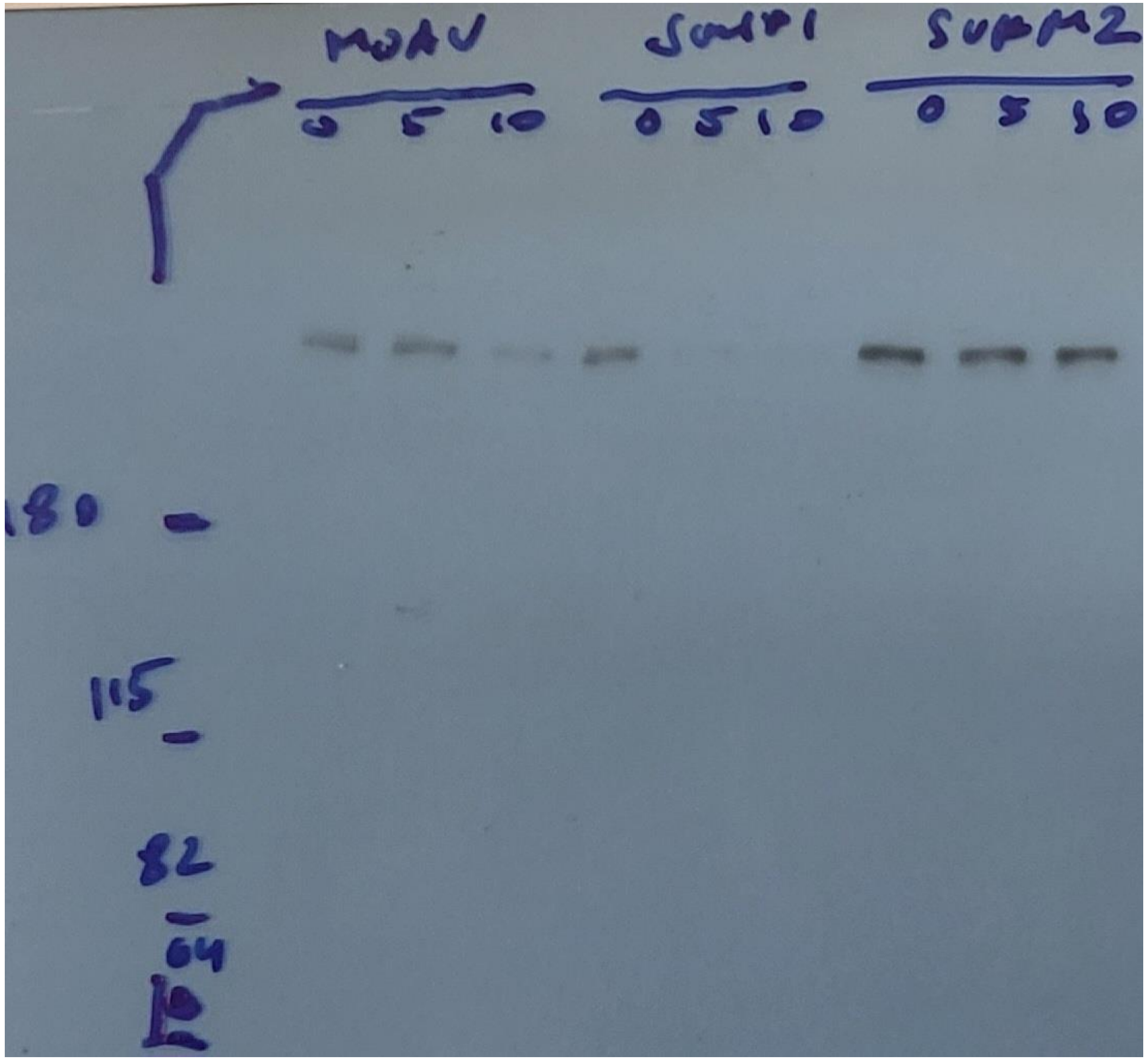

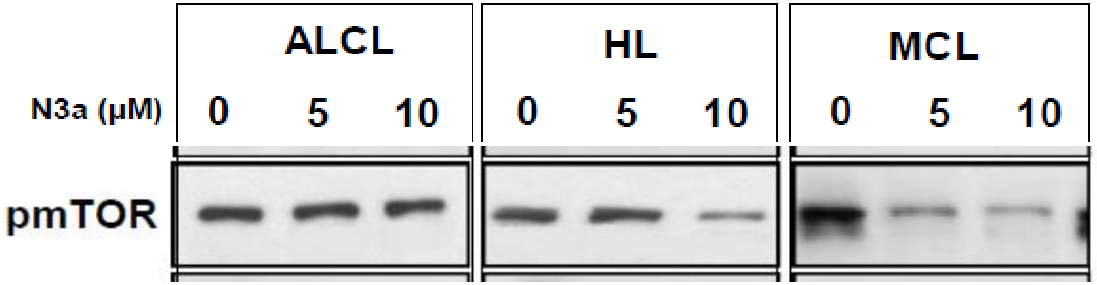

rbS6 in HL/NHL -/+N3a

M	MDA-V			JMP-1			SUP-M2			
	**0**	**5**	**10**	**0**	**5**	**10**	**0**	**5**	**10**	**N3a (μM)**

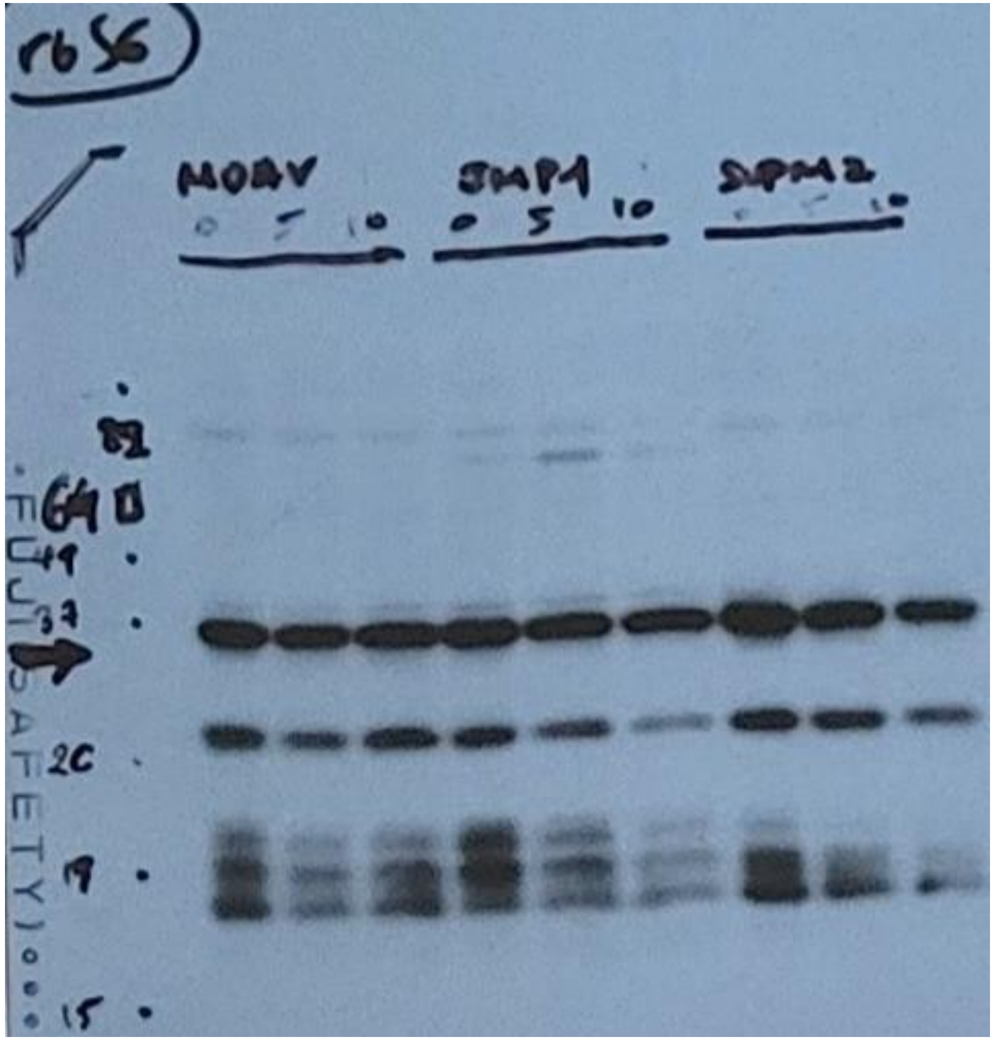

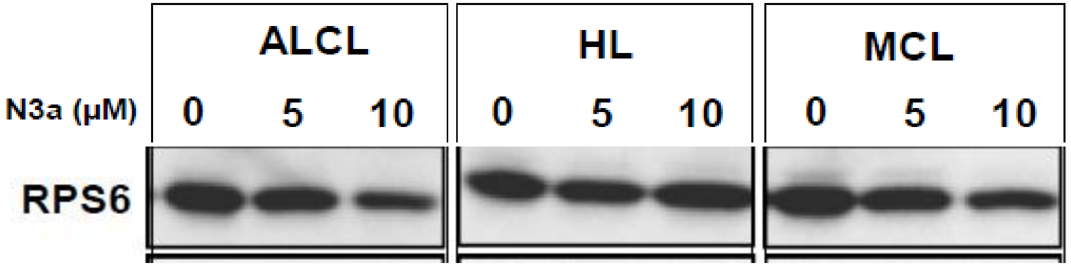

p-rbS6 in HL/NHL -/+N3a

M MDA-V JMP-1 SUP-M2

0 5 10 0 5 10 0 5 10 N3a (µM)

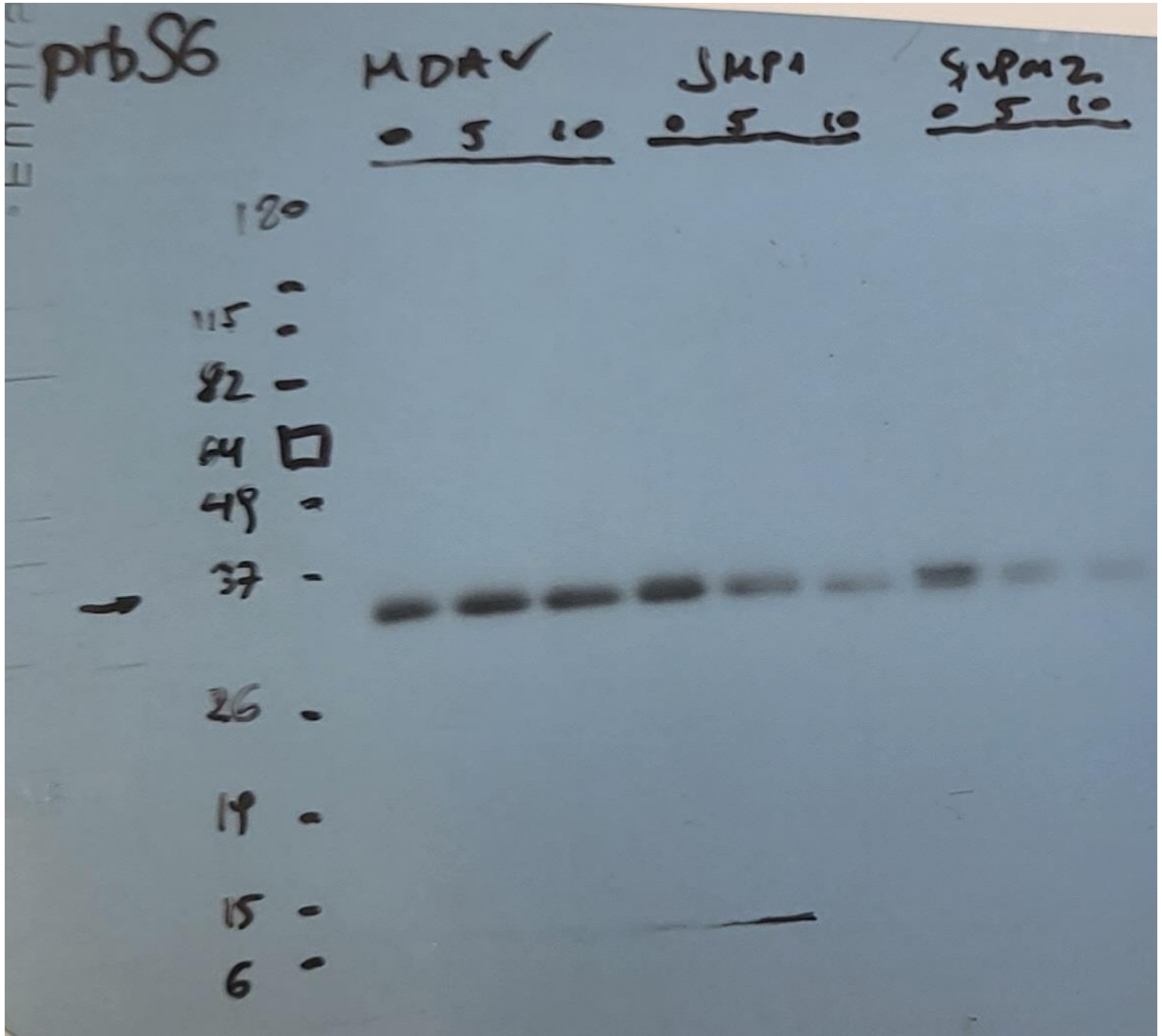

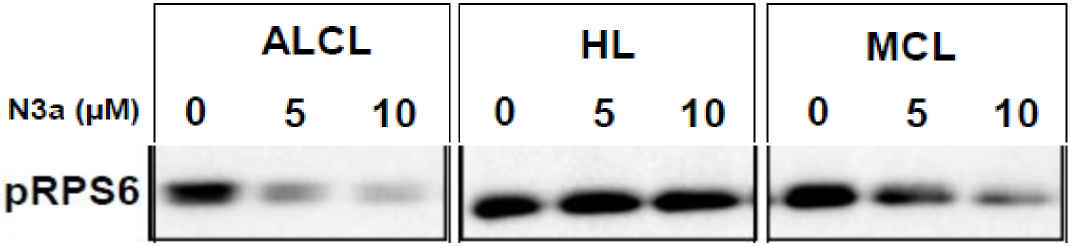

4EBP1 in HL/NHL -/+N3a

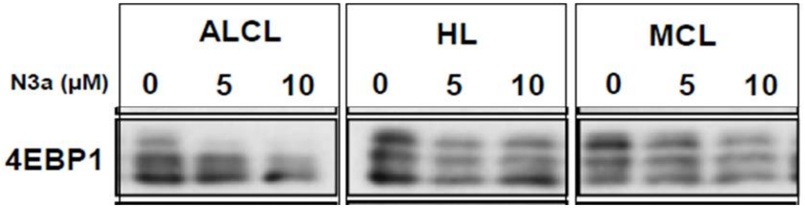

M   MDA-V   JMP-1   SUP-M2

0 5 10 0 5 10 0 5 10 N3a (µM)

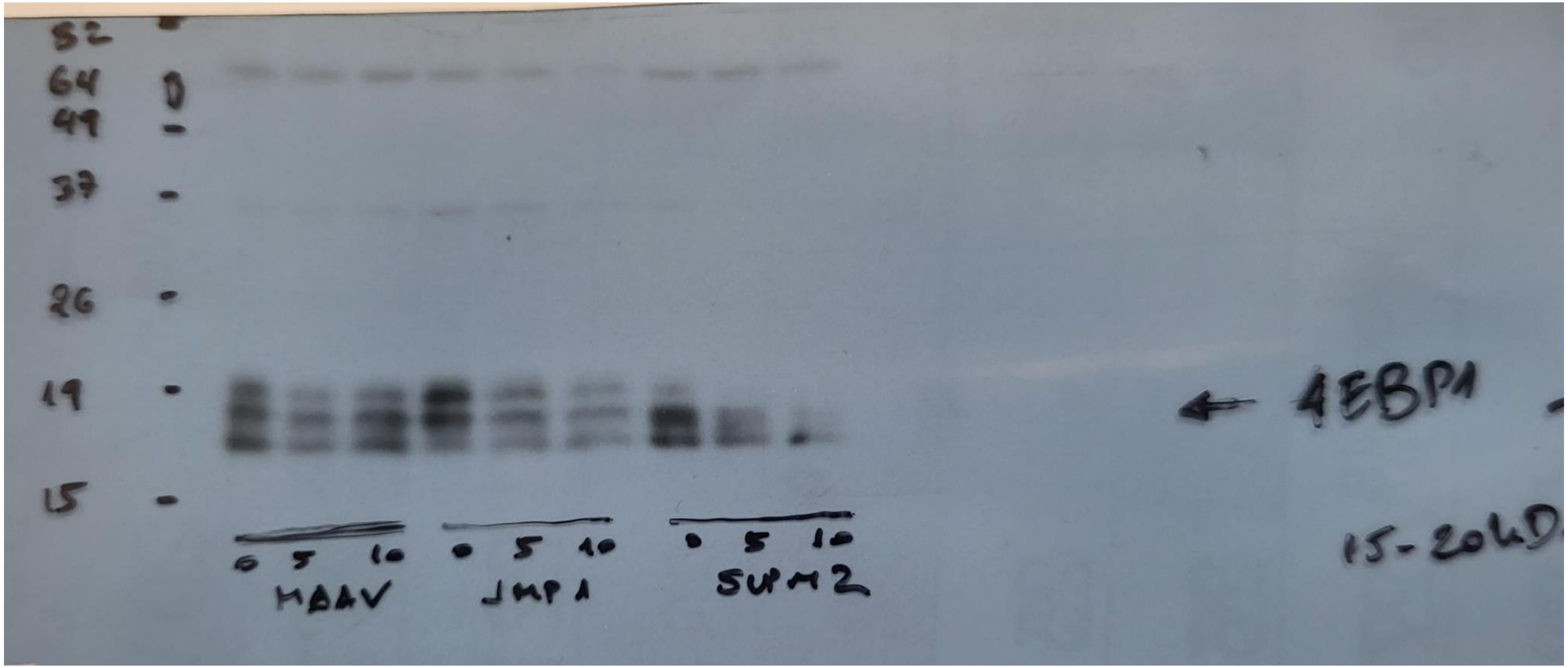

p- 4EBP1 in HL/NHL -/+N3a

M MDA-V JMP-1 SUP-M2

0 5 10 0 5 10 0 5 10 N3a (µM)

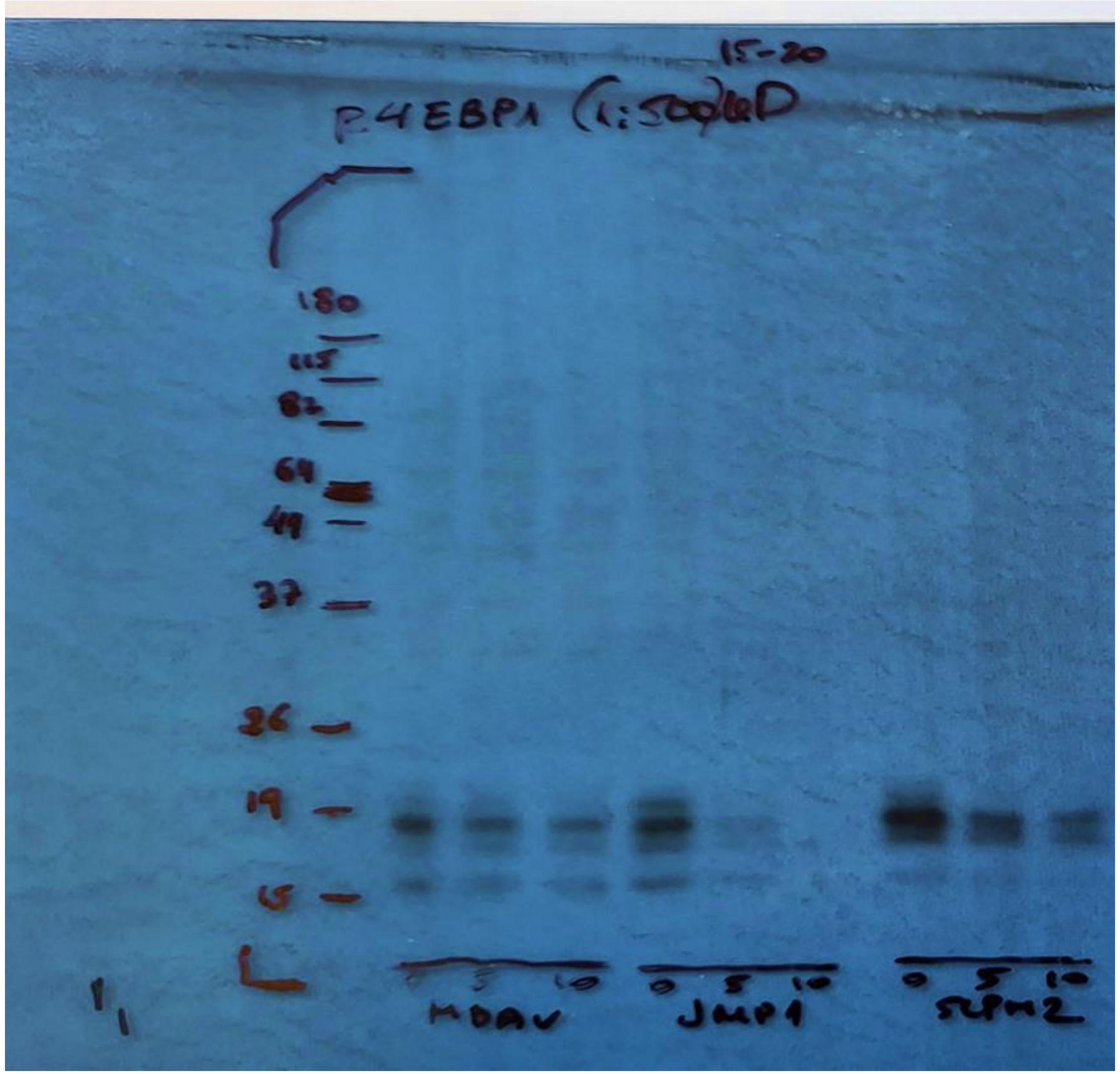

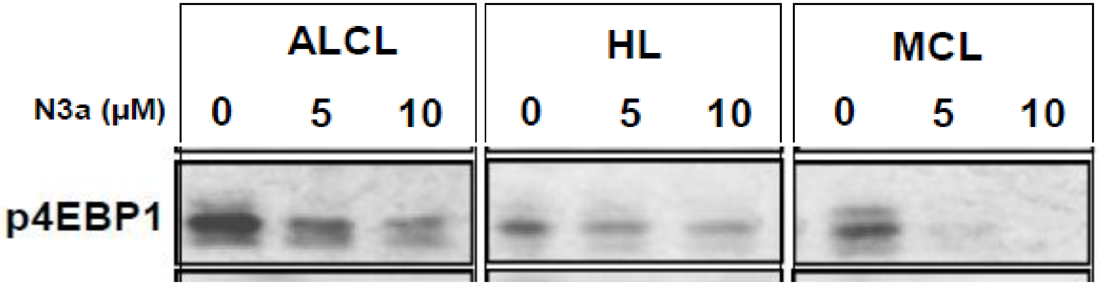

eiF4GI expression in HL/NHL -/+N3a

M	MDA-V			JMP-1			SUP-M2			
	**0**	**5**	**10**	**0**	**5**	**10**	**0**	**5**	**10**	**N3a (µM)**

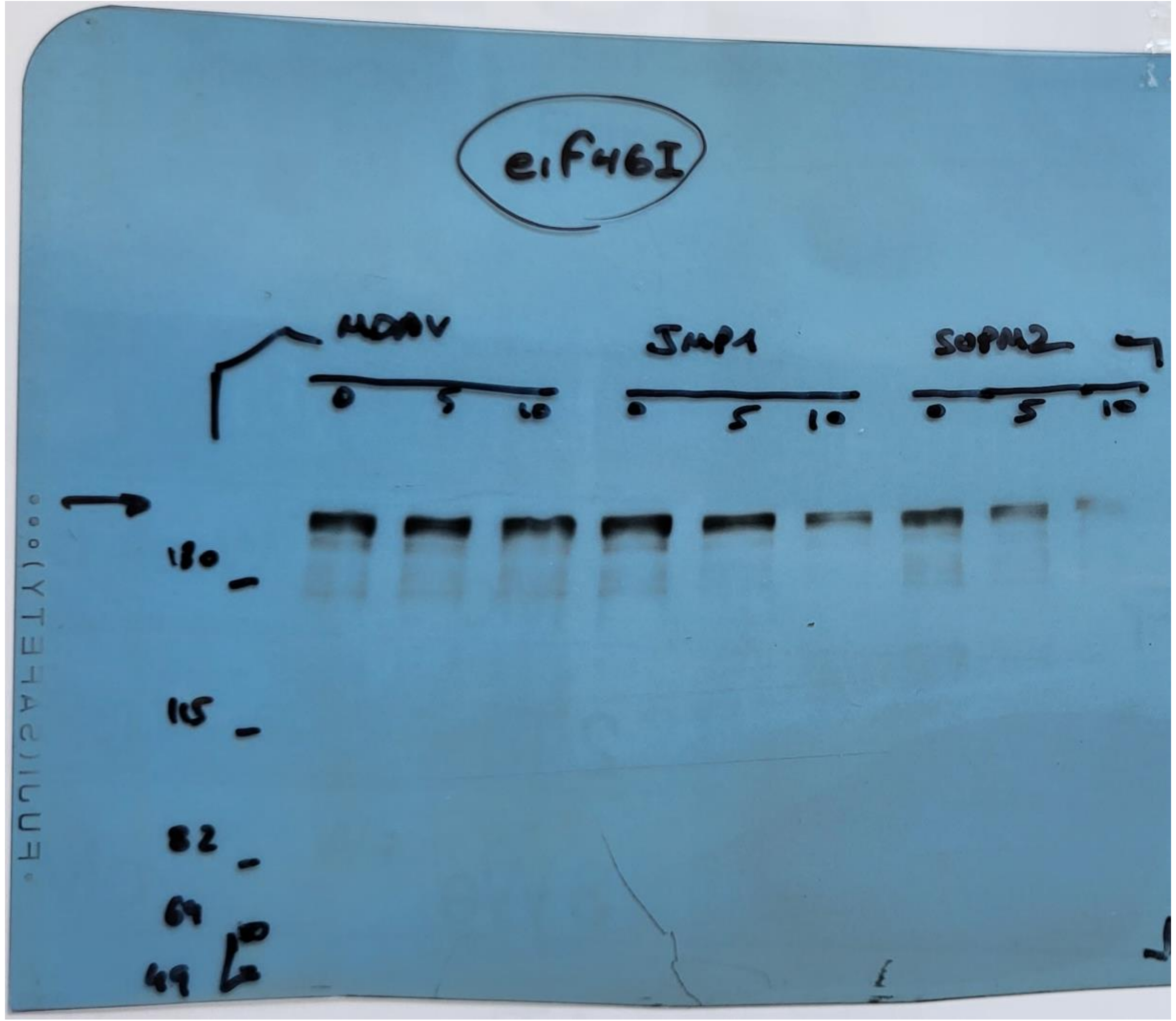

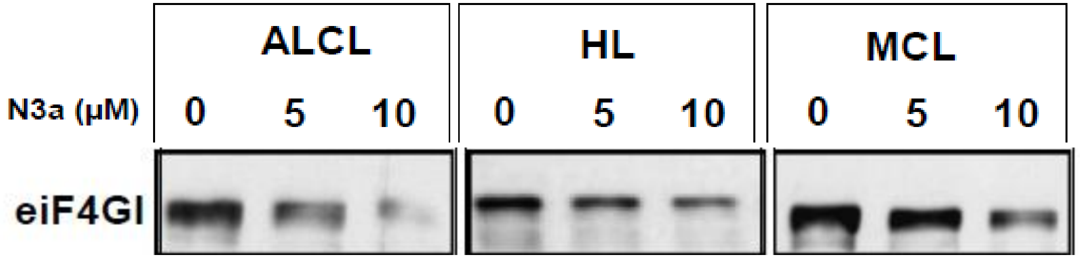

eiF4E expression in HL/NHL -/+N3a

M MDA-V JMP-1 SUP-M2

0 5 10 0 5 10 0 5 10 **N3a (µM)**

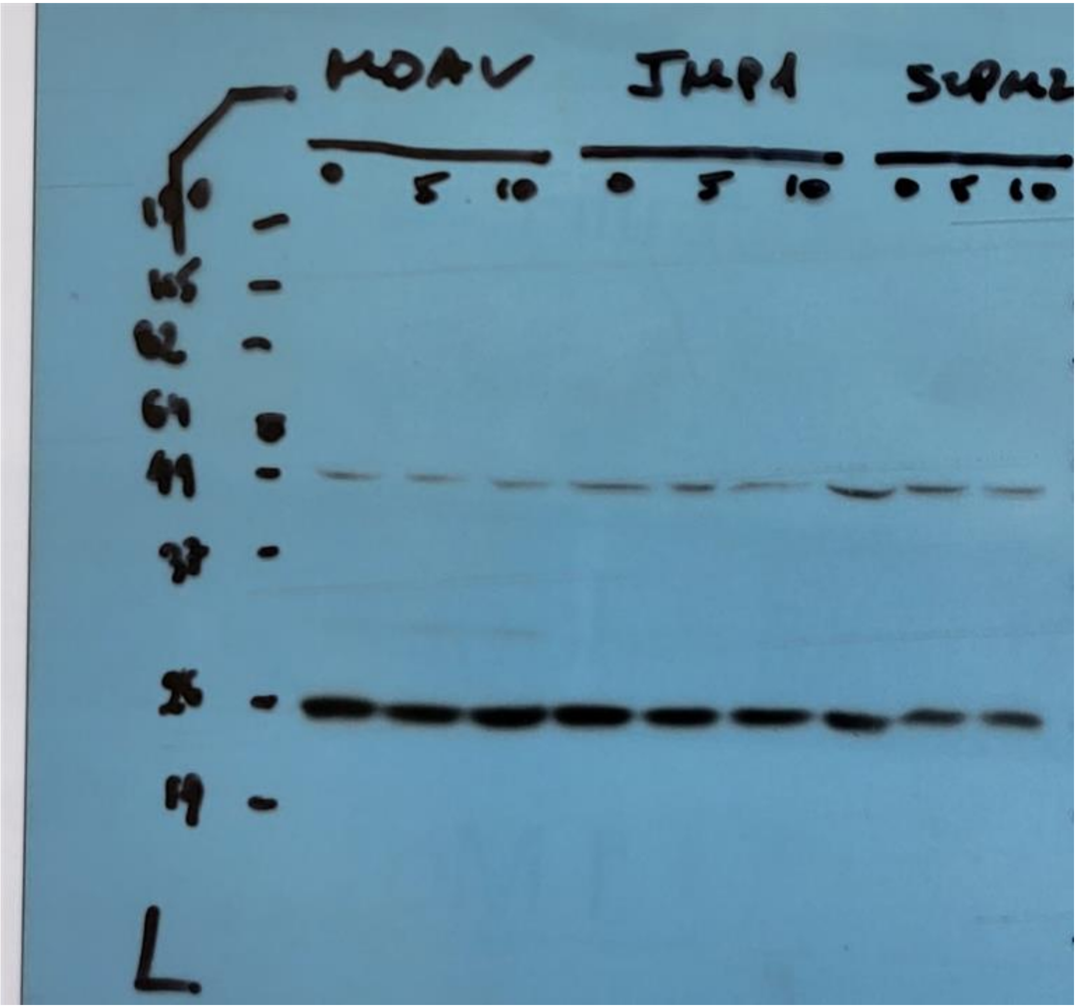

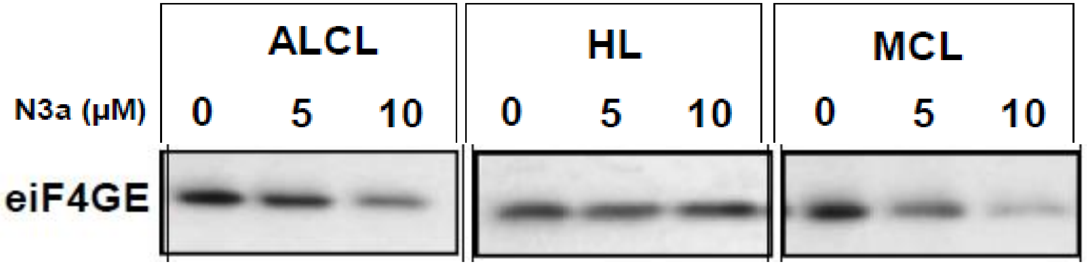

p53 in HL/NHL -/+N3a

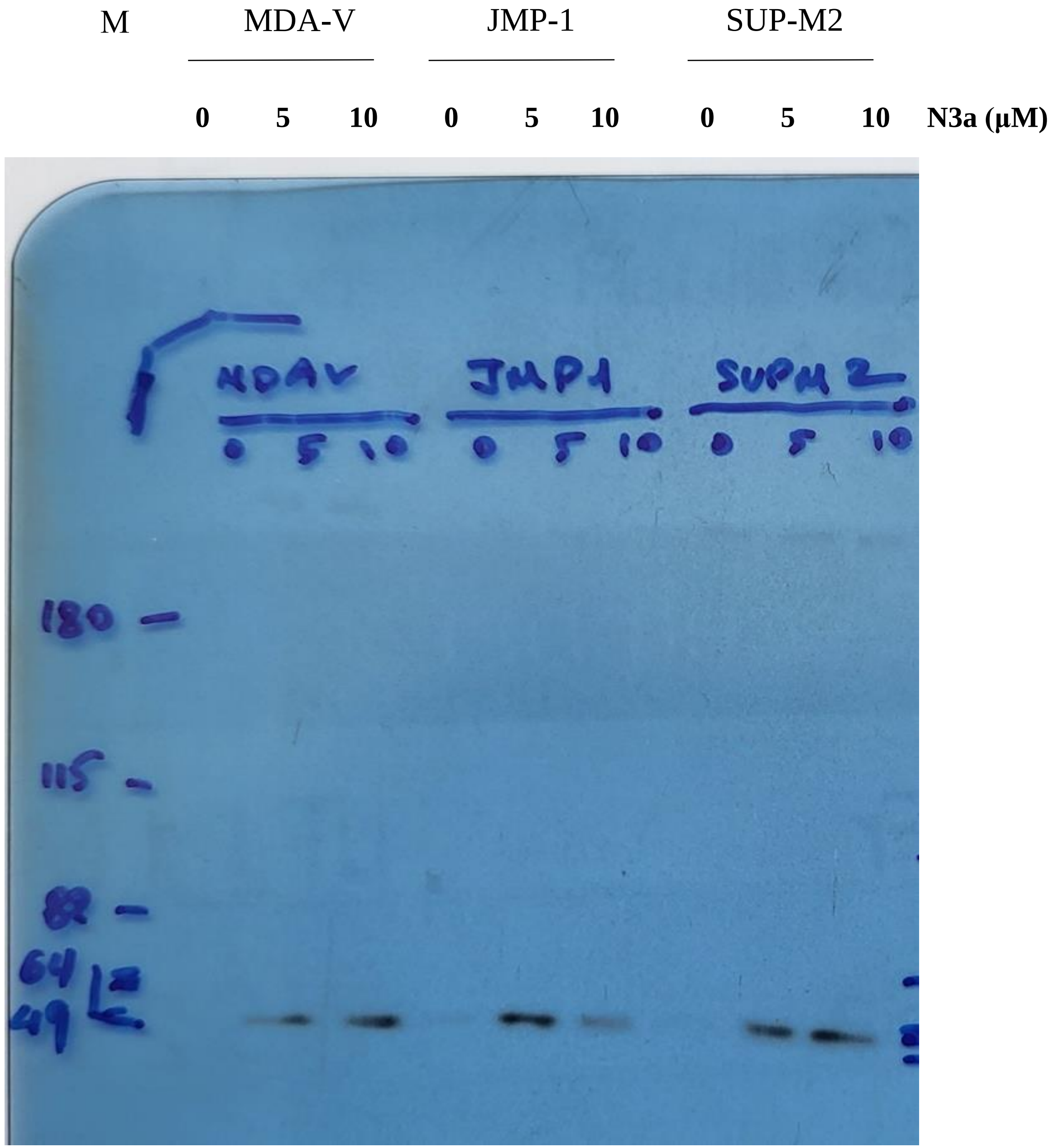

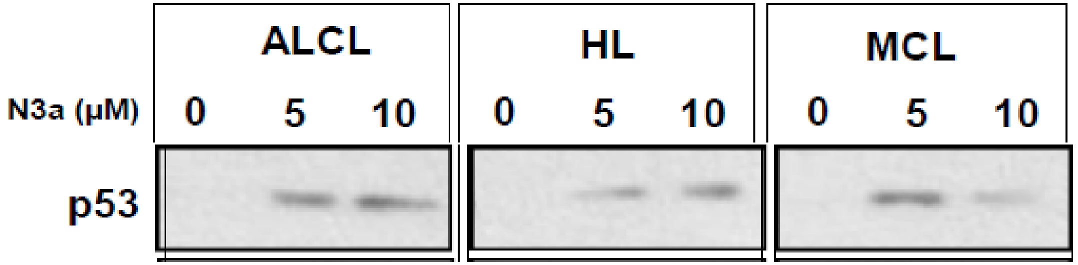

Monitoring N3a's-effect on mTOR-related proteins in HL/NHL cells

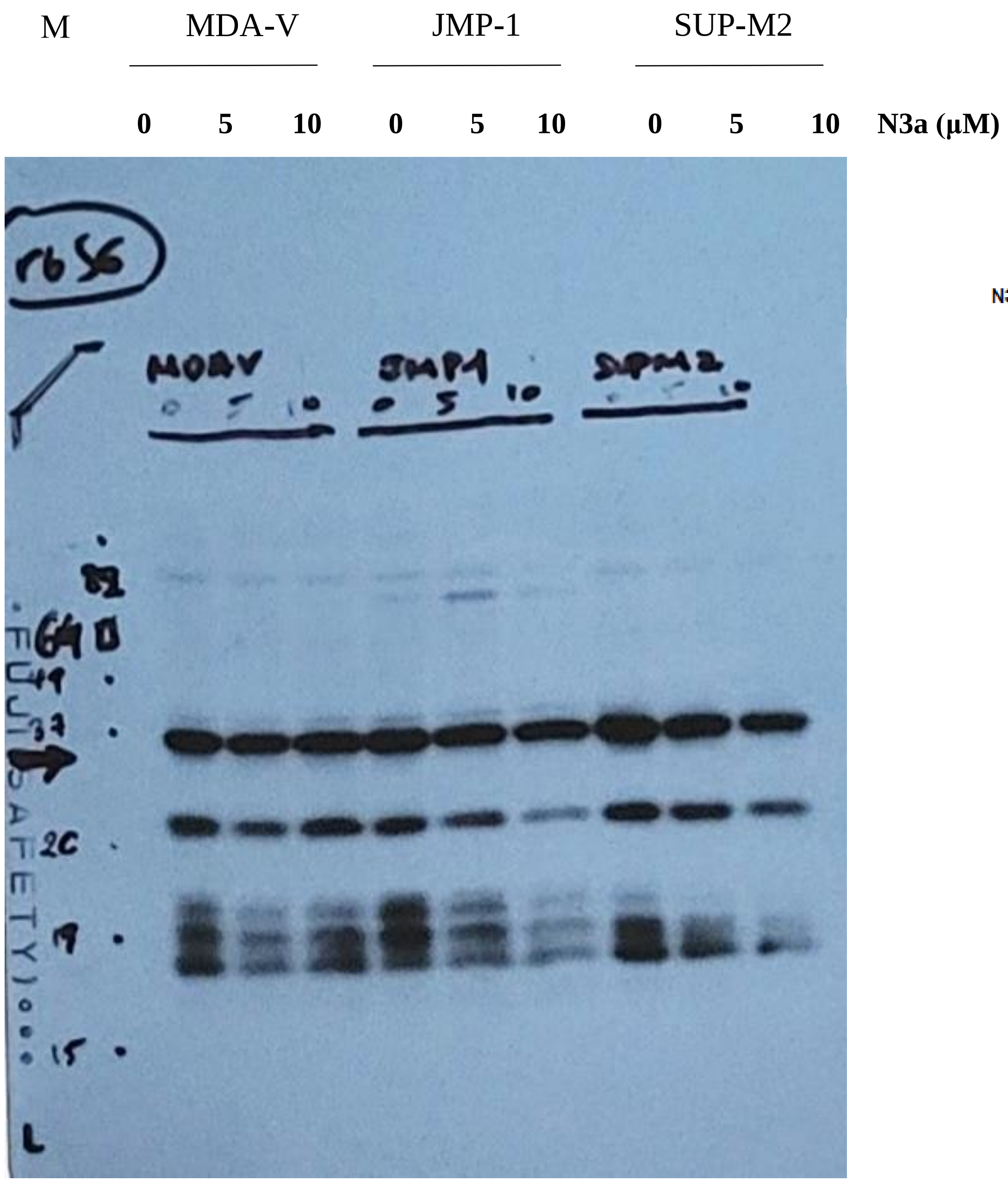

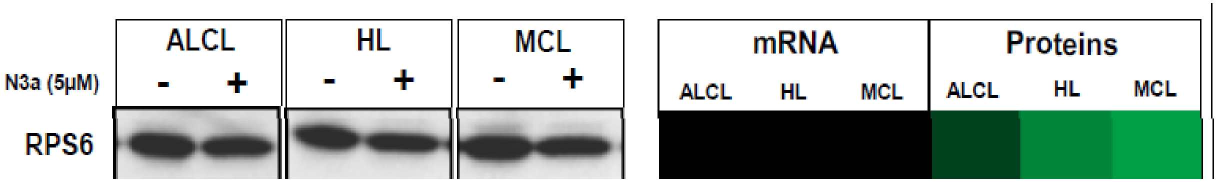

Monitoring N3a's-effect on mTOR-related proteins in HL/NHL cells

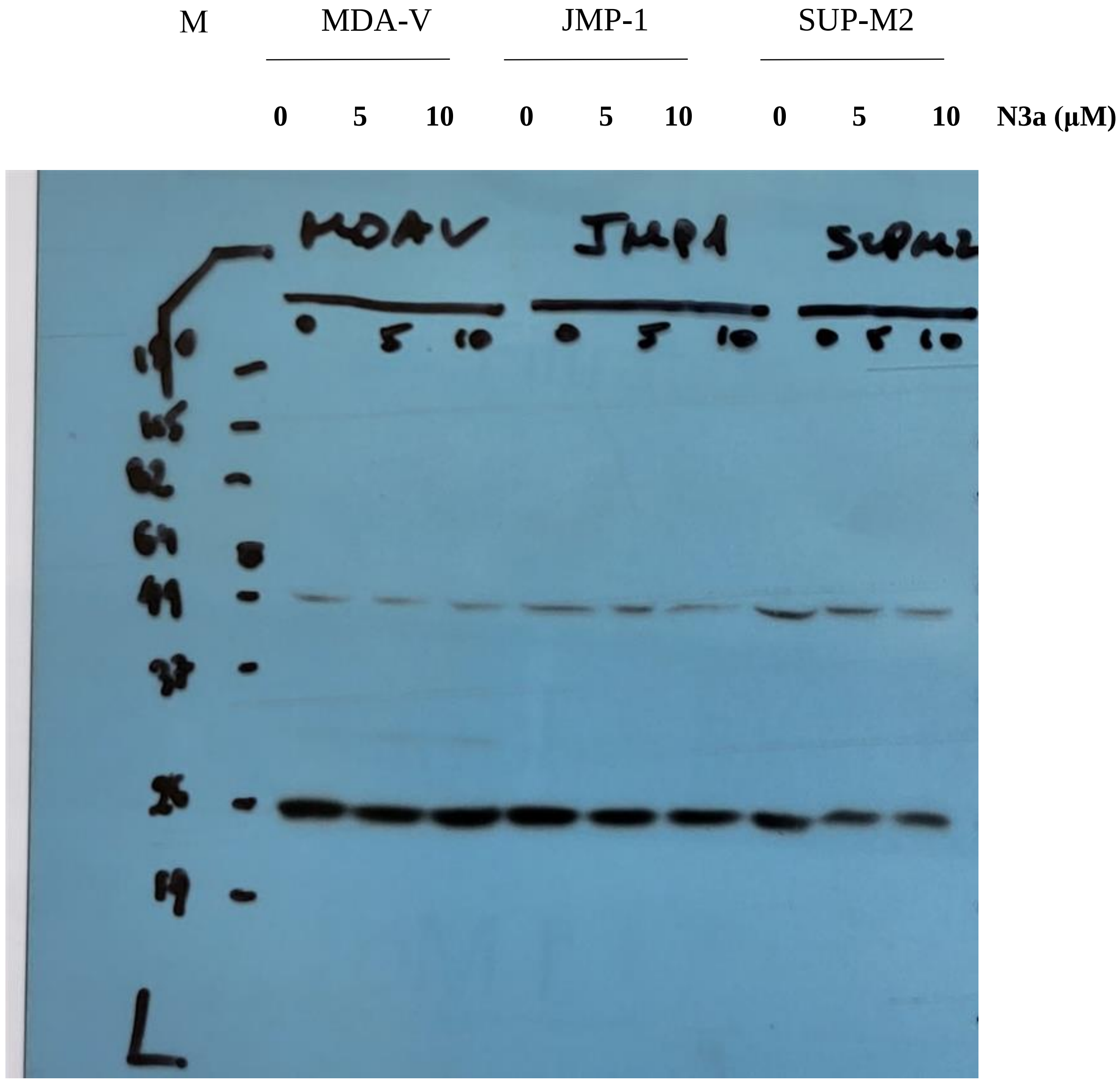

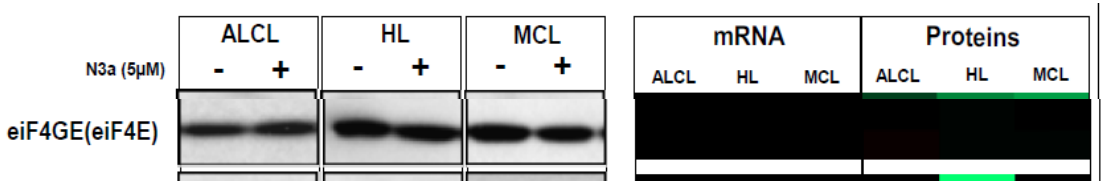

Monitoring N3a’s-effect on mTOR-related proteins in HL/NHL cells

M	MDA-V			JMP-1			SUP-M2			
	0	5	10	0	5	10	0	5	10	N3a (µM)

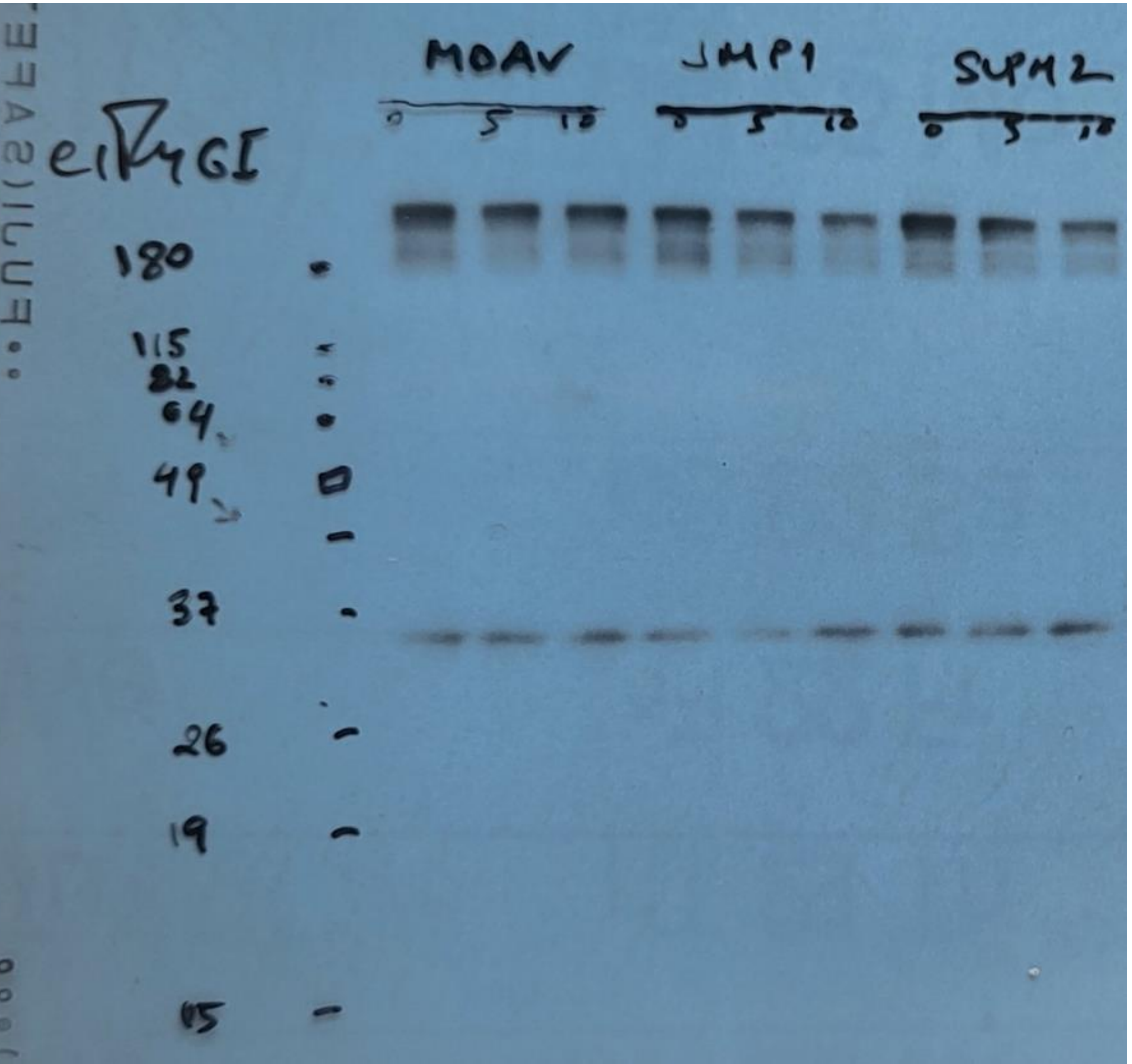

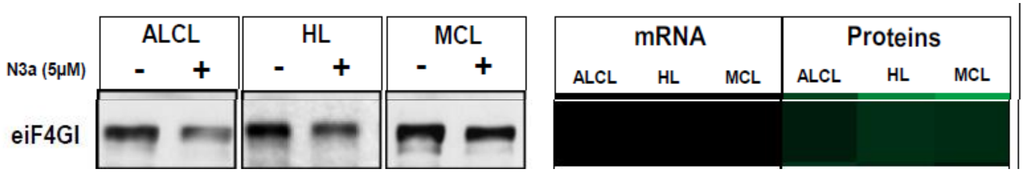

AMPKa

M MDA-V JMP-1 SUP-M2

0 5 10 0 5 10 0 5 10 N3a (µM)

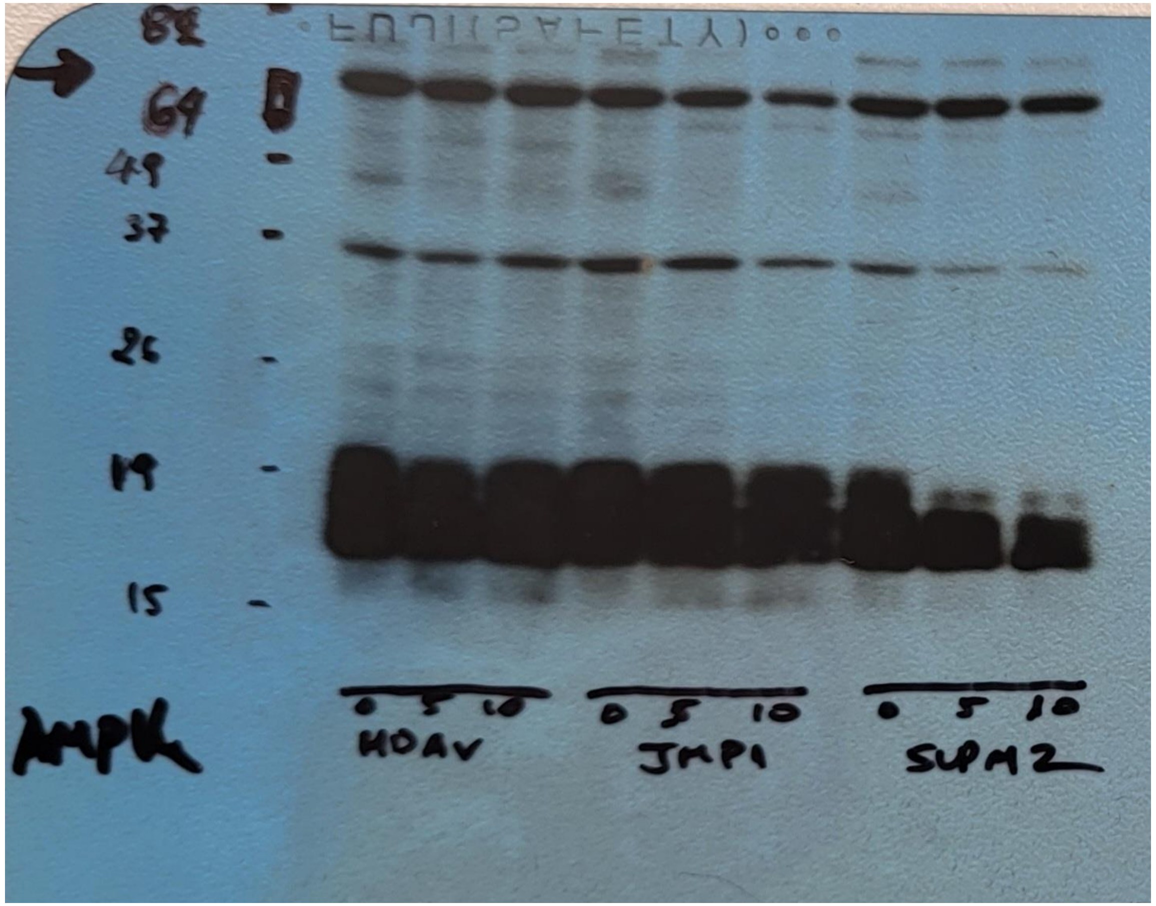

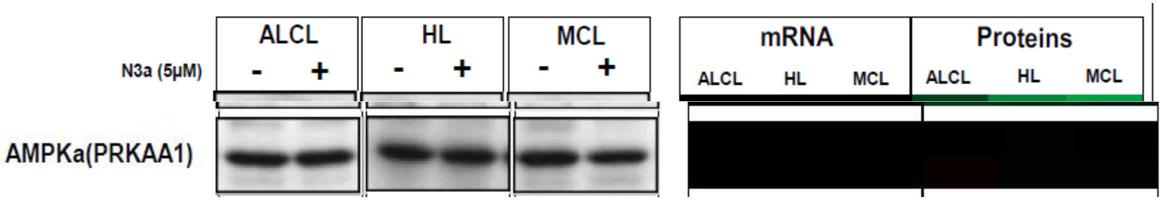

p70S6k

M MDA-V JMP-1 SUP-M2

	MDA-V			JMP-1			SUP-M2			
M	0	5	10	0	5	10	0	5	10	N3a (µM)

SUP-M2

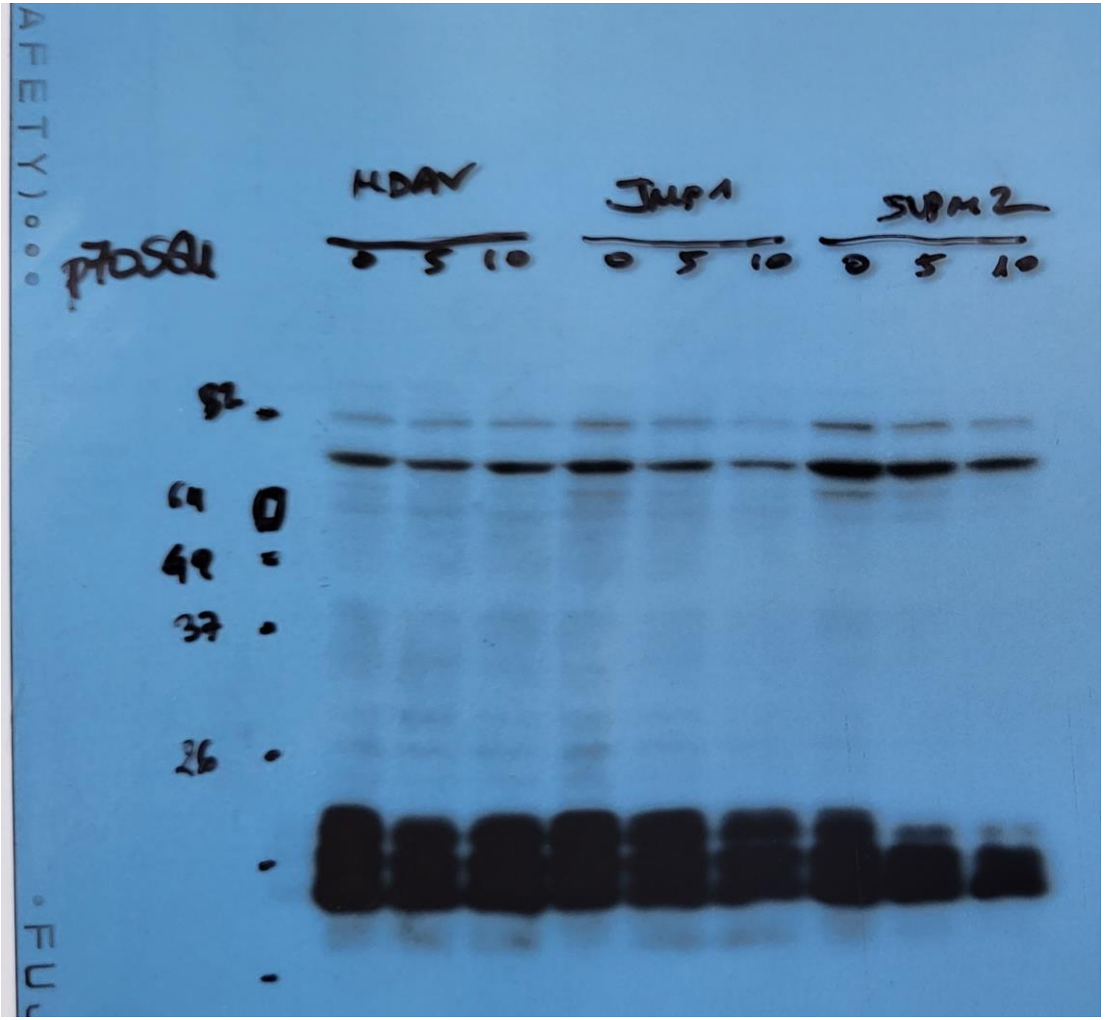

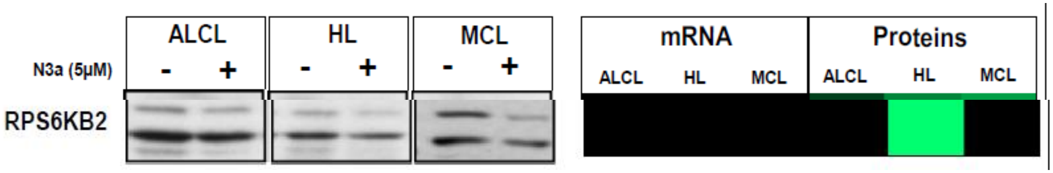

β-actin in HL/NHL -/+N3a

M MDA-V JMP-1 SUP-M2

0 5 10 0 5 10 0 5 10 N3a (μM)

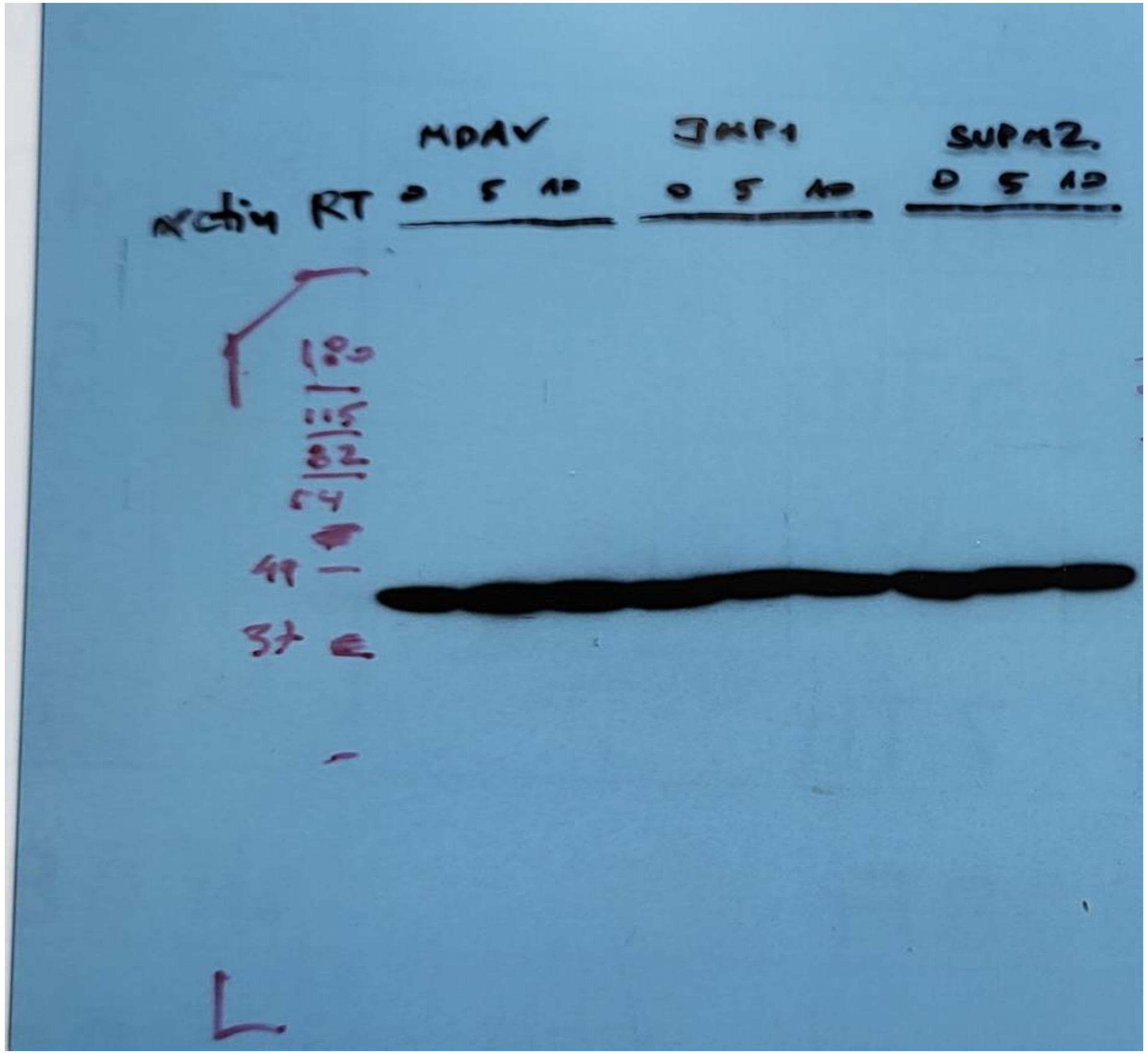

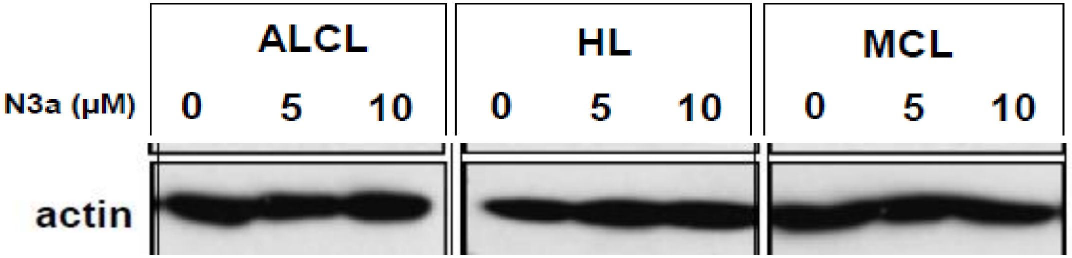

Monitoring N3a's-effect on autophagy-related proteins in HL/NHL cells

ATG4B expression in HL/NHLs -/+N3a

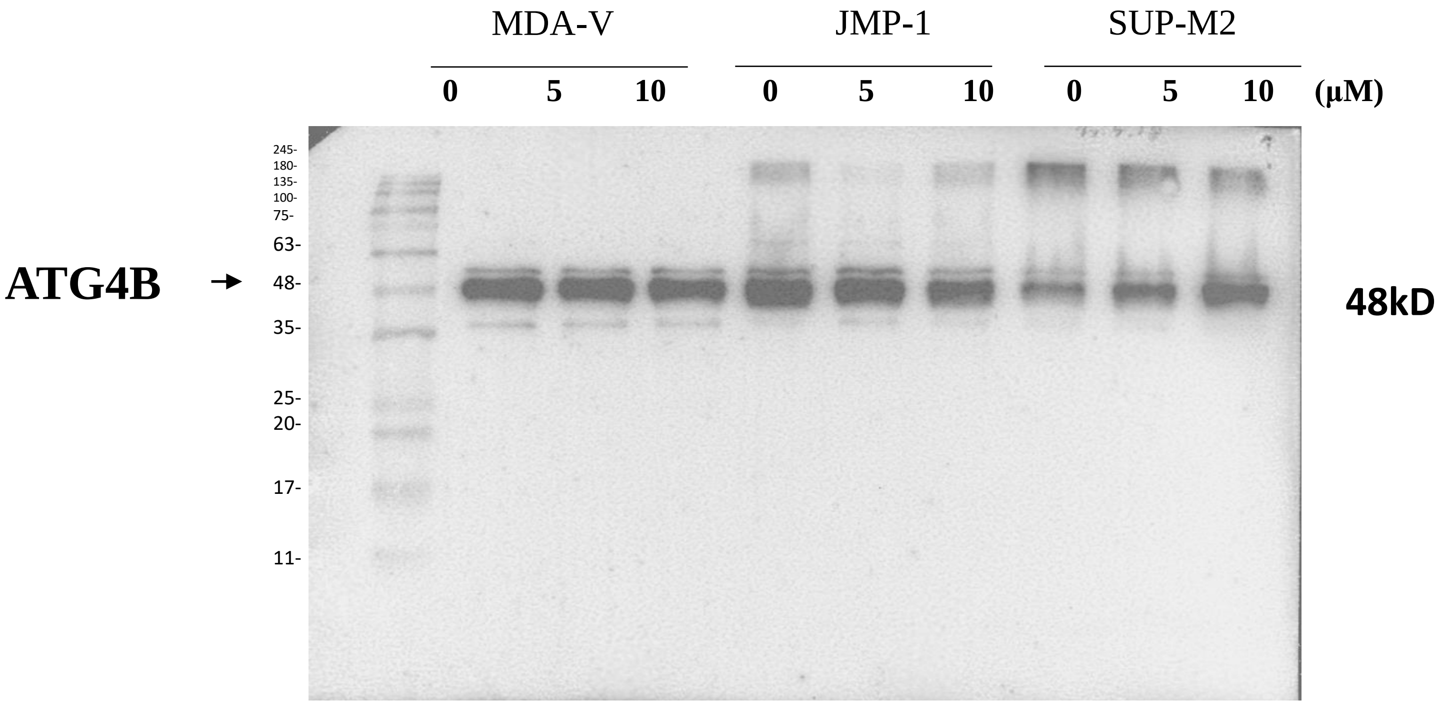

Increased protein levels of LC3-II in HL/NHLs after N3a

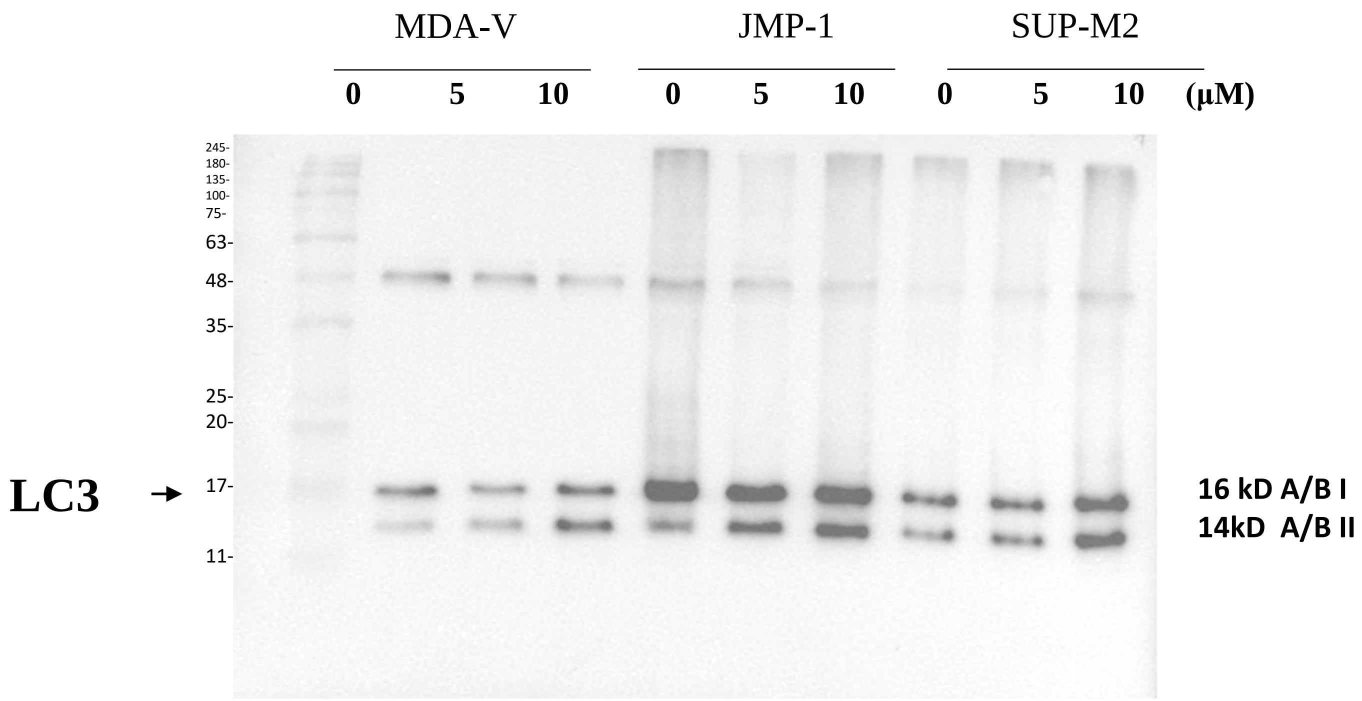

Stable ATG5/ATG12 expression in HL/NHL -/+N3a

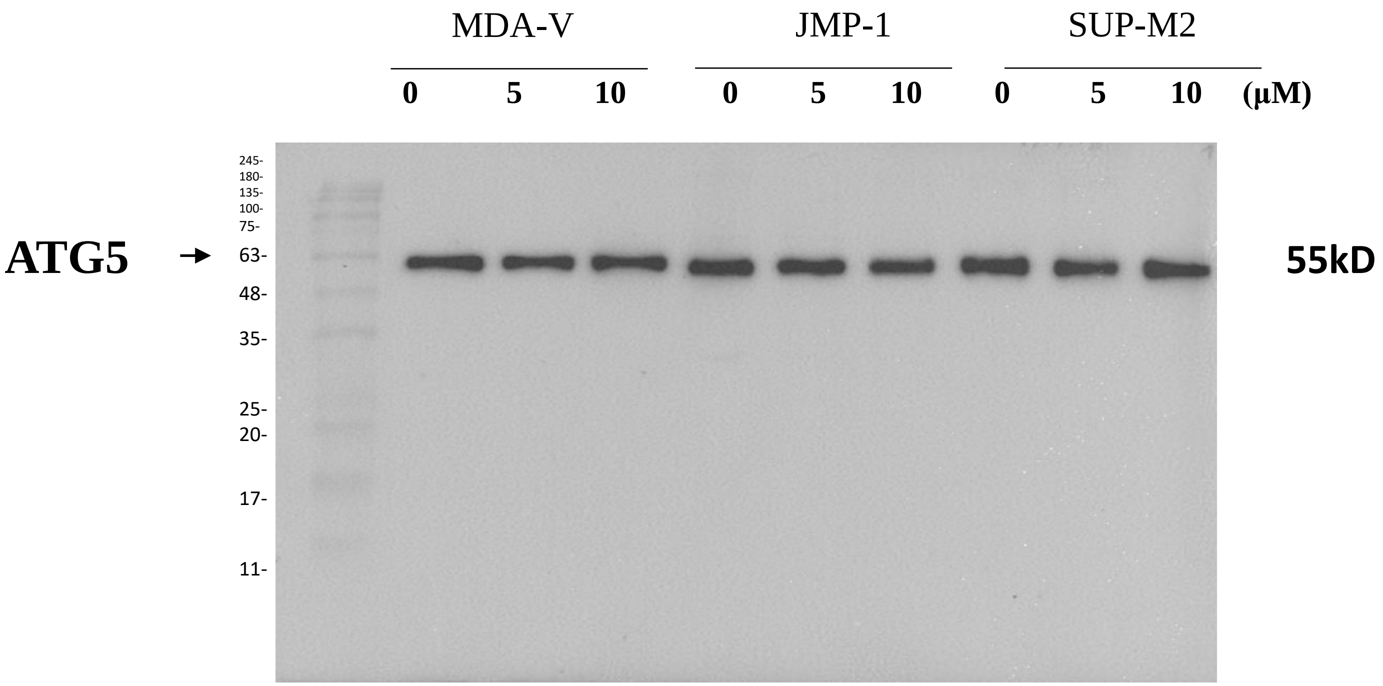

B-Actin as a loading control in HL/NHLs -/+N3a

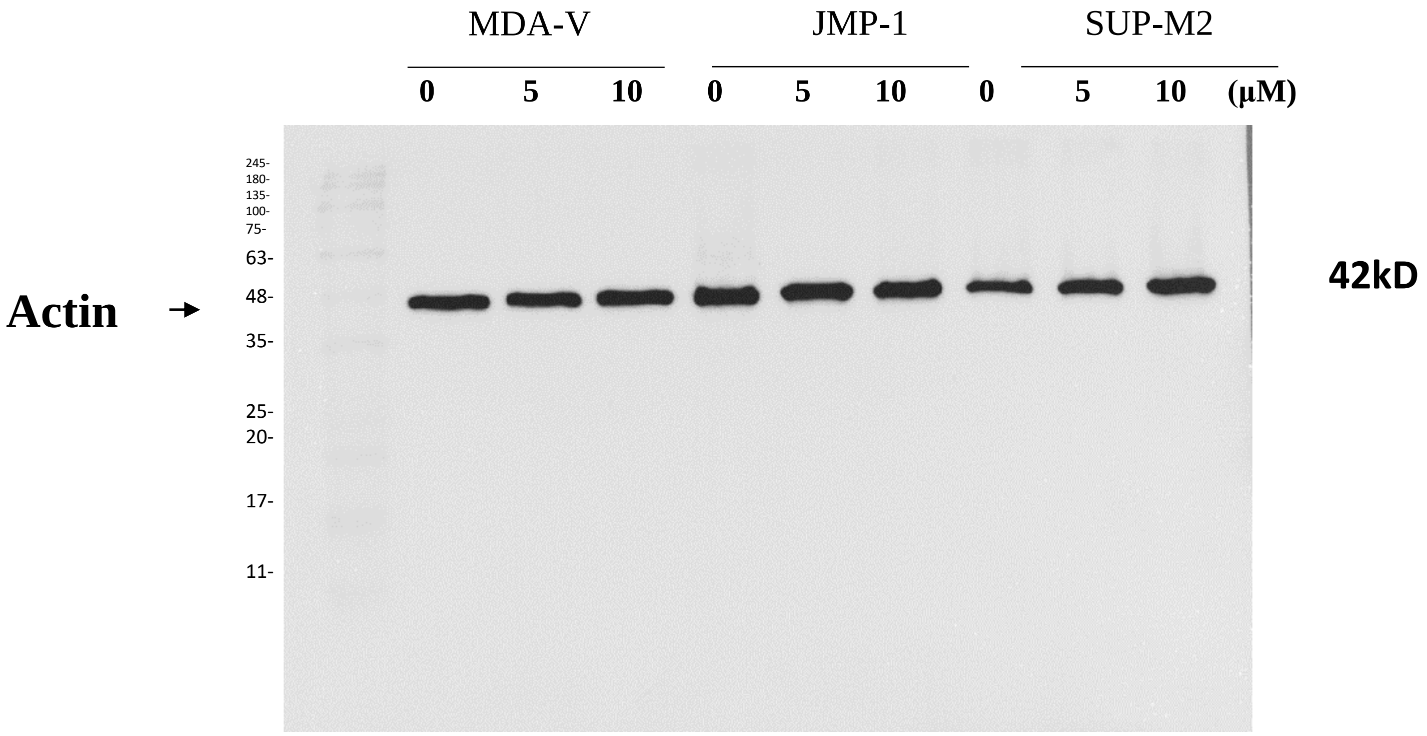

Monitoring N3a's-effect on NF-kB signaling proteins in HL/NHL cells

Decreased p65 expression in HL/MCL -/+N3a

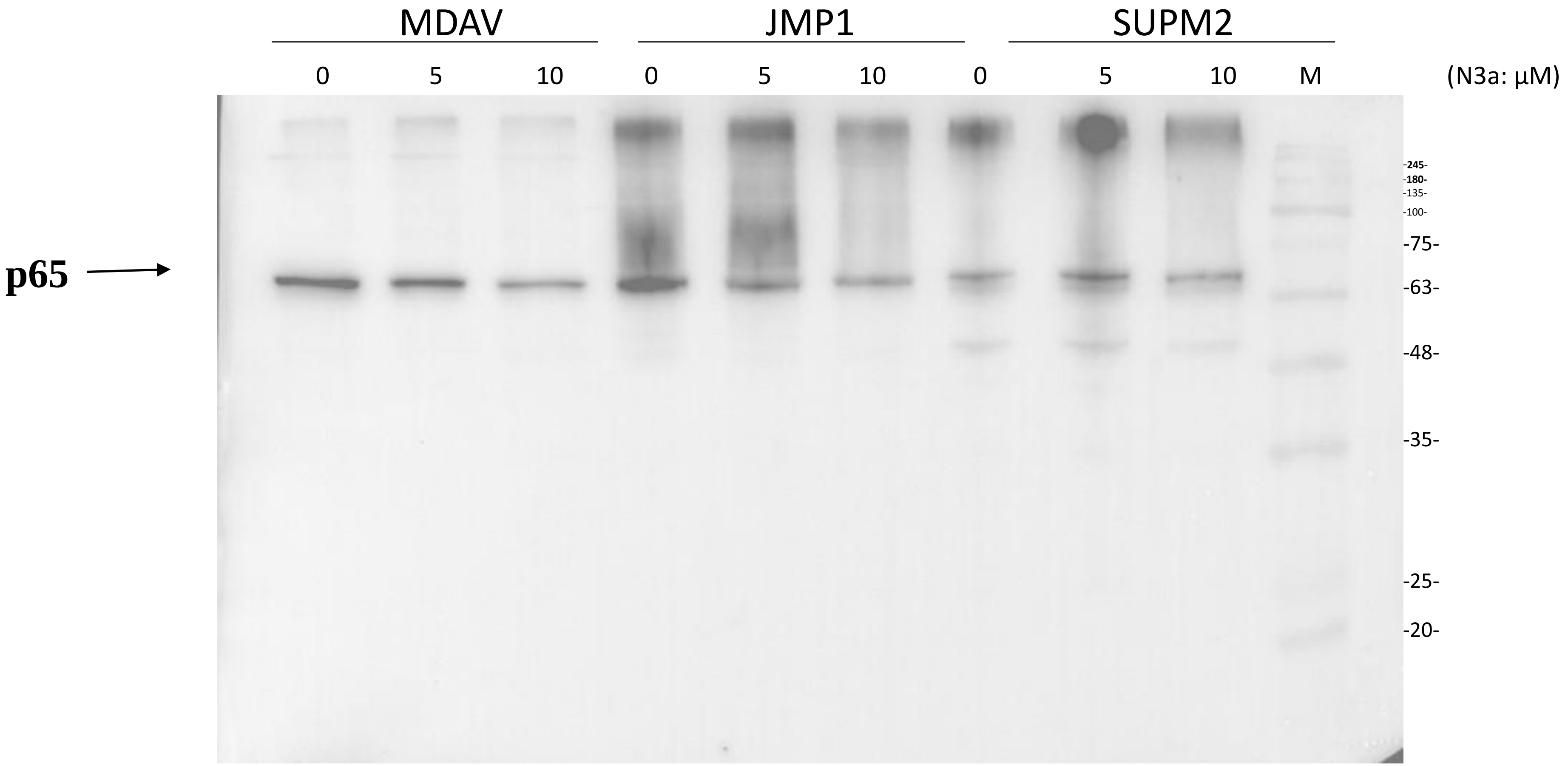

B-Actin as a loading control in HL/NHLs -/+N3a

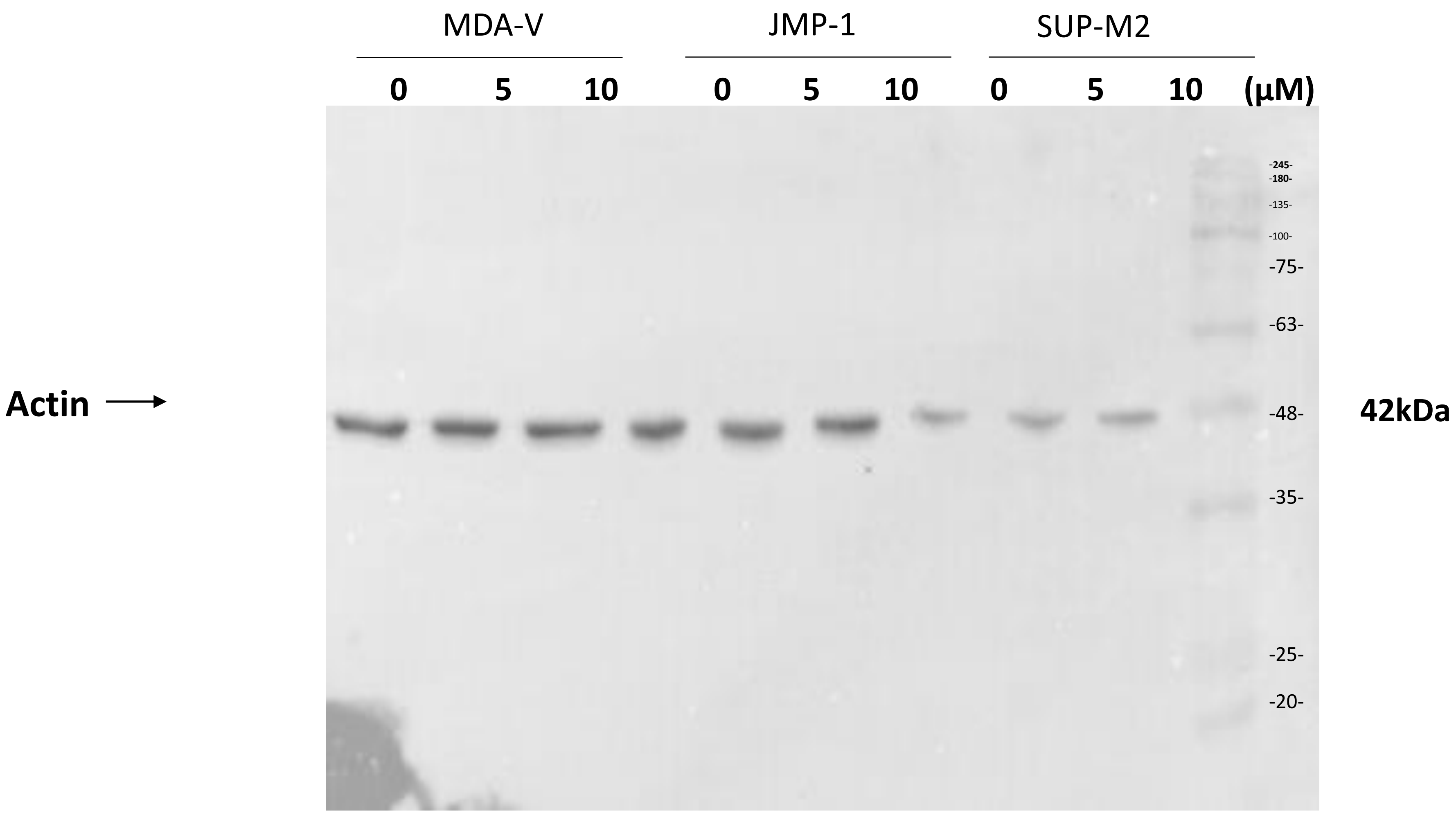

Monitoring N3a’s-effect on transcription factors TIGAR, IKAROS in HL/NHL cells

Increased TIGAR expression in HL/MCL -/+N3a

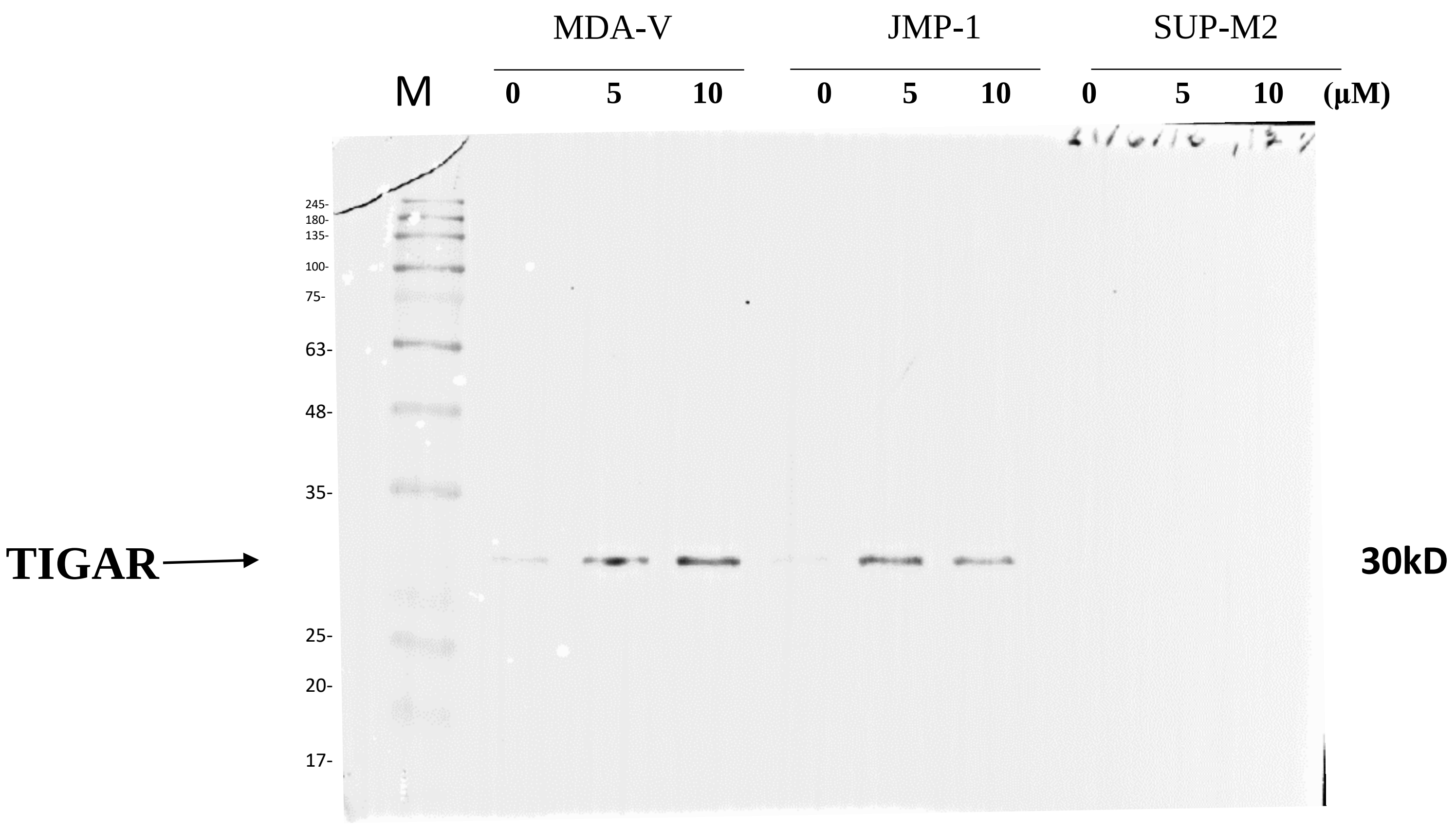

Reduced Ikaros expression in HL -/+N3a

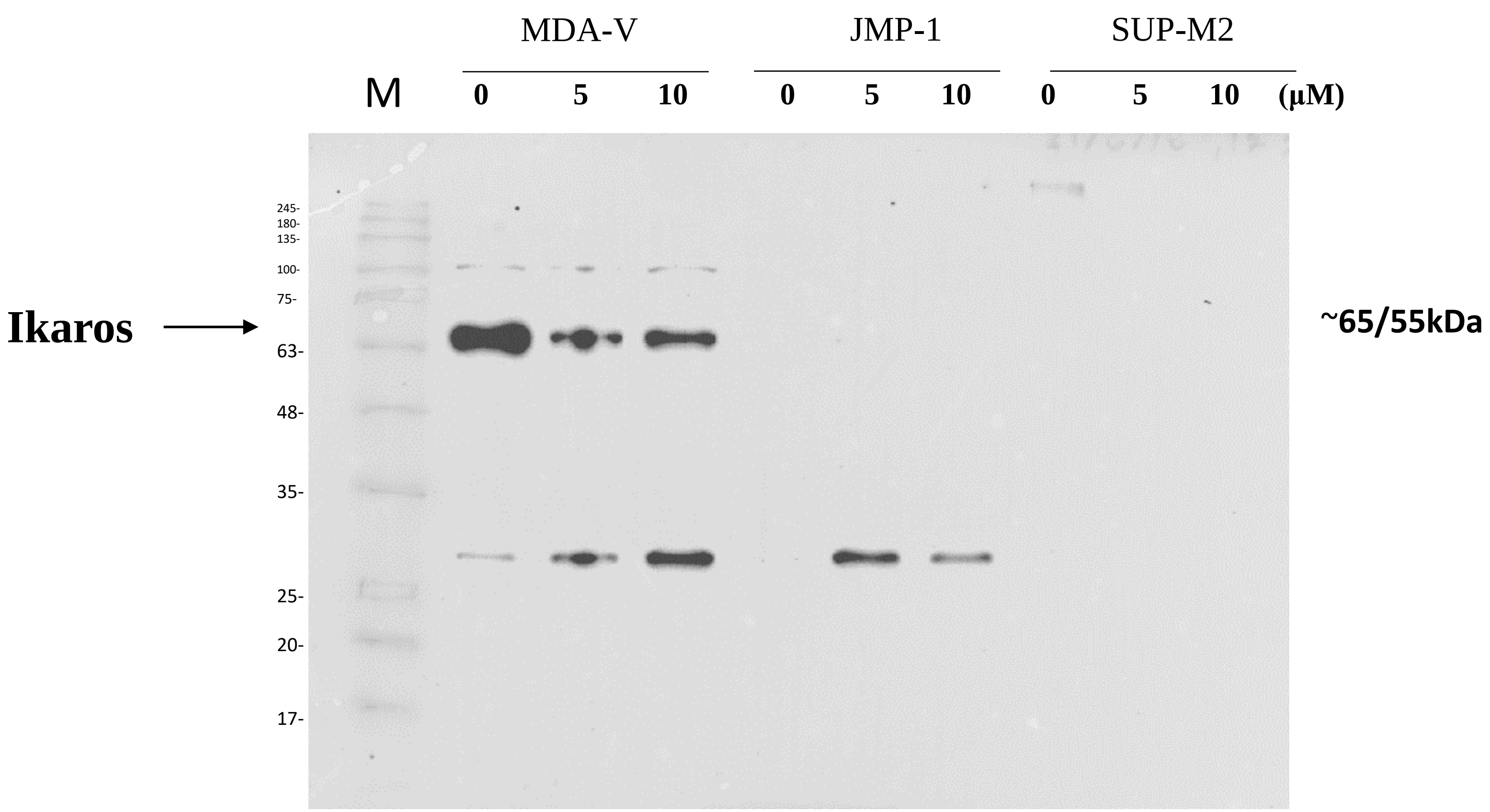

B-Actin as a loading control in HL/NHLs -/+N3a

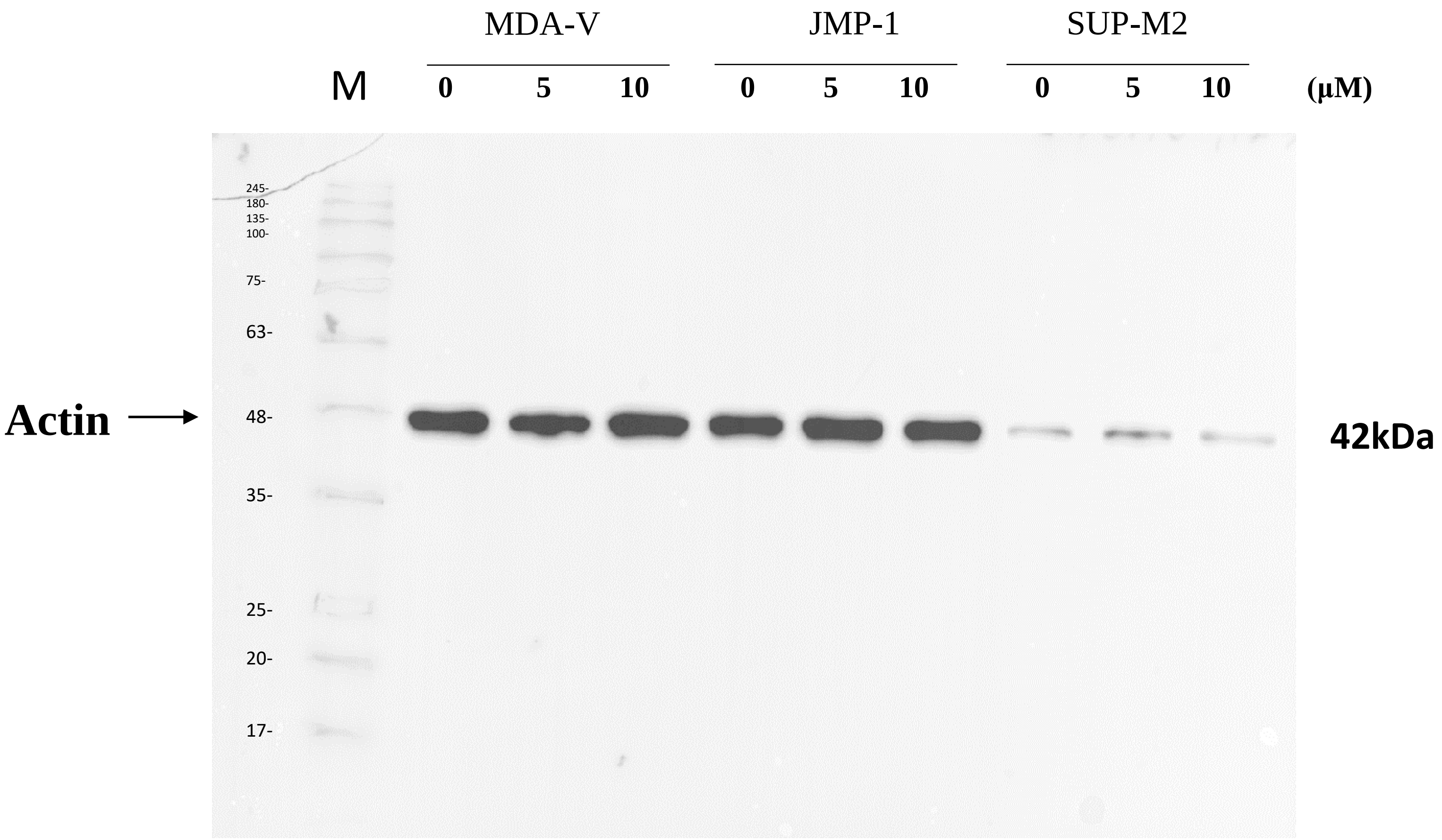

Monitoring N3a's-effect on HSP90, HSP70 in HL/NHL cells

Reduced HSP90 expression in ALCL, HL -/+N3a

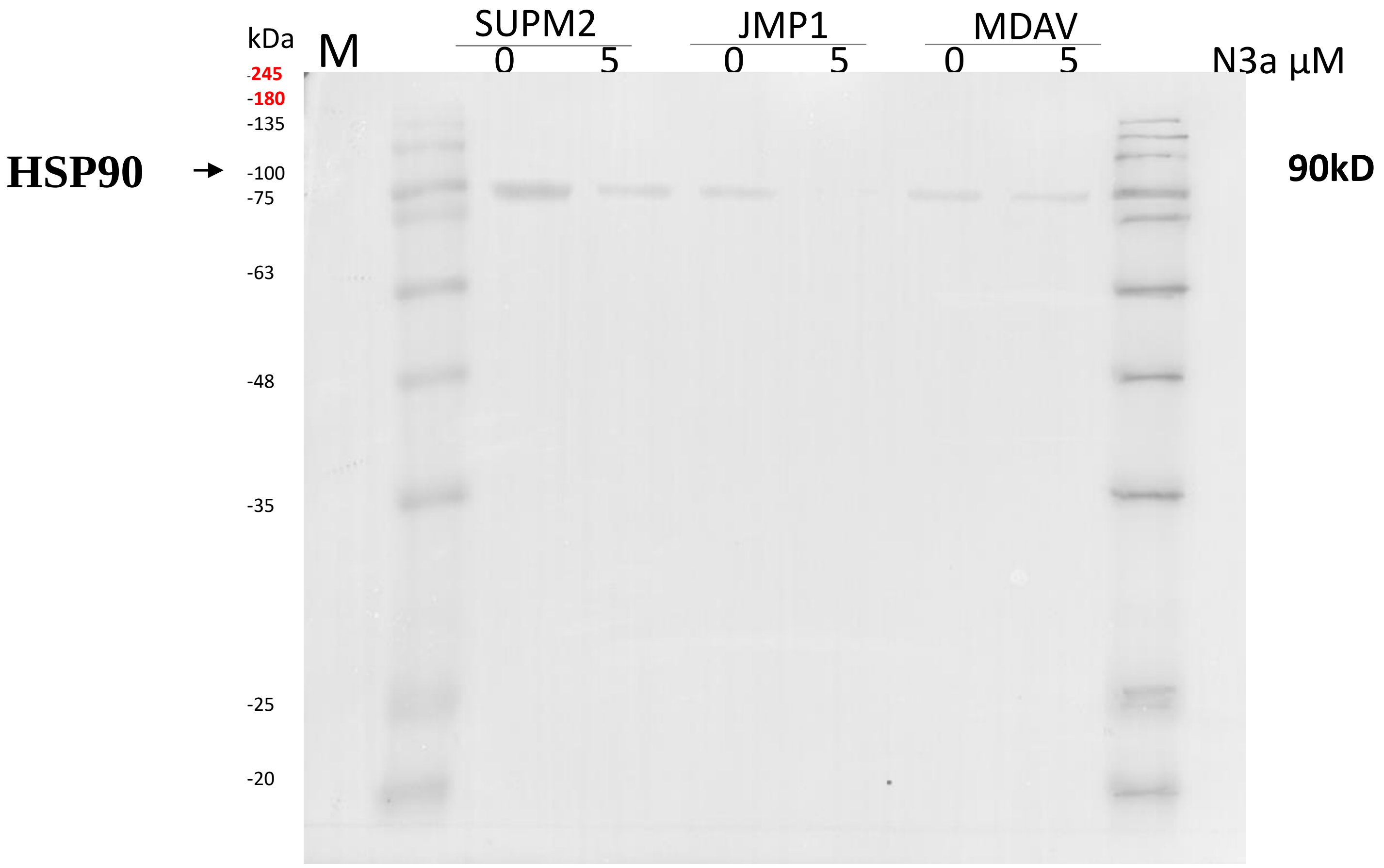

HSP70 expression in HL, NHL -/+N3a

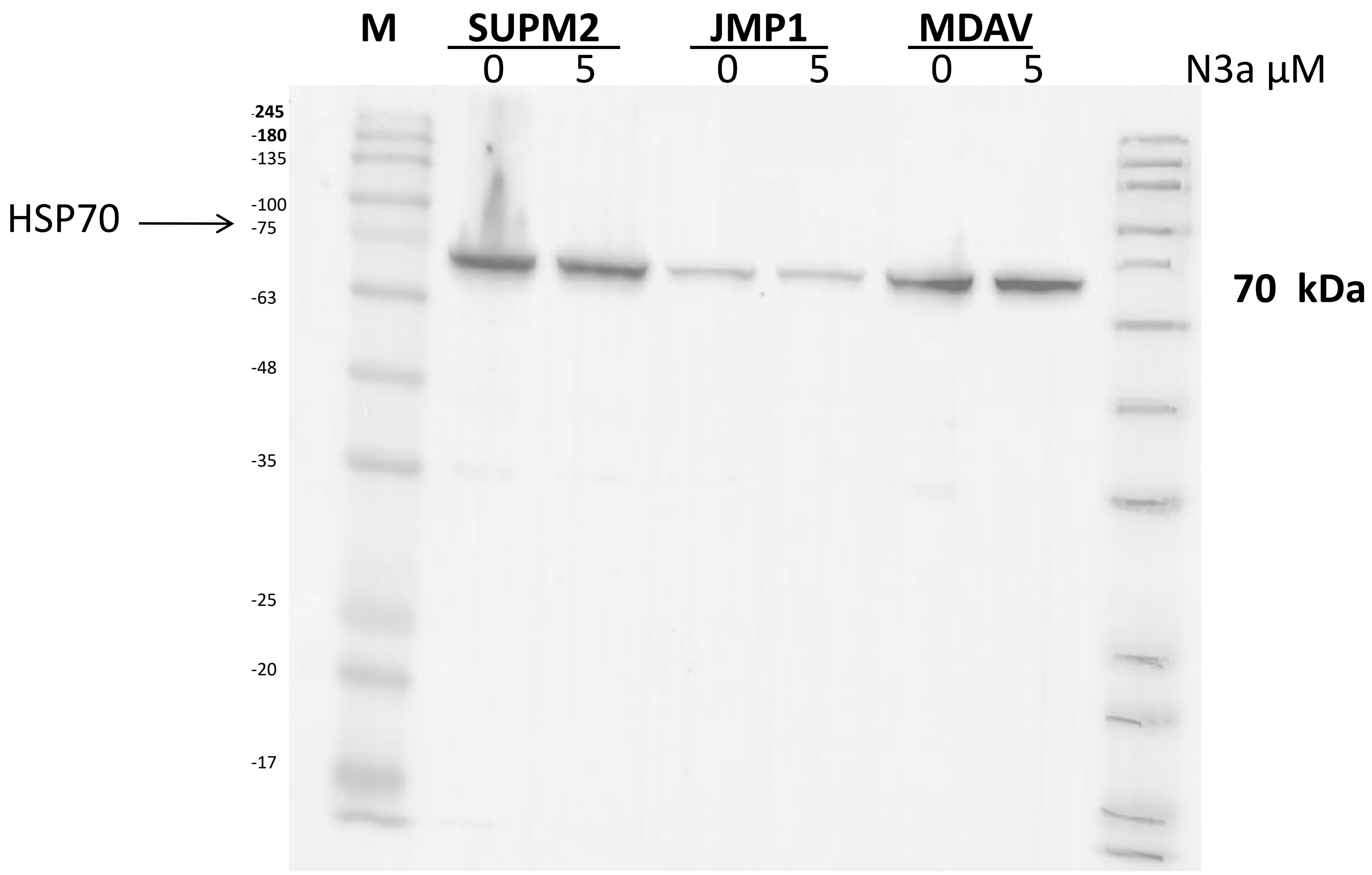

Actin in HL/NHL -/+N3a

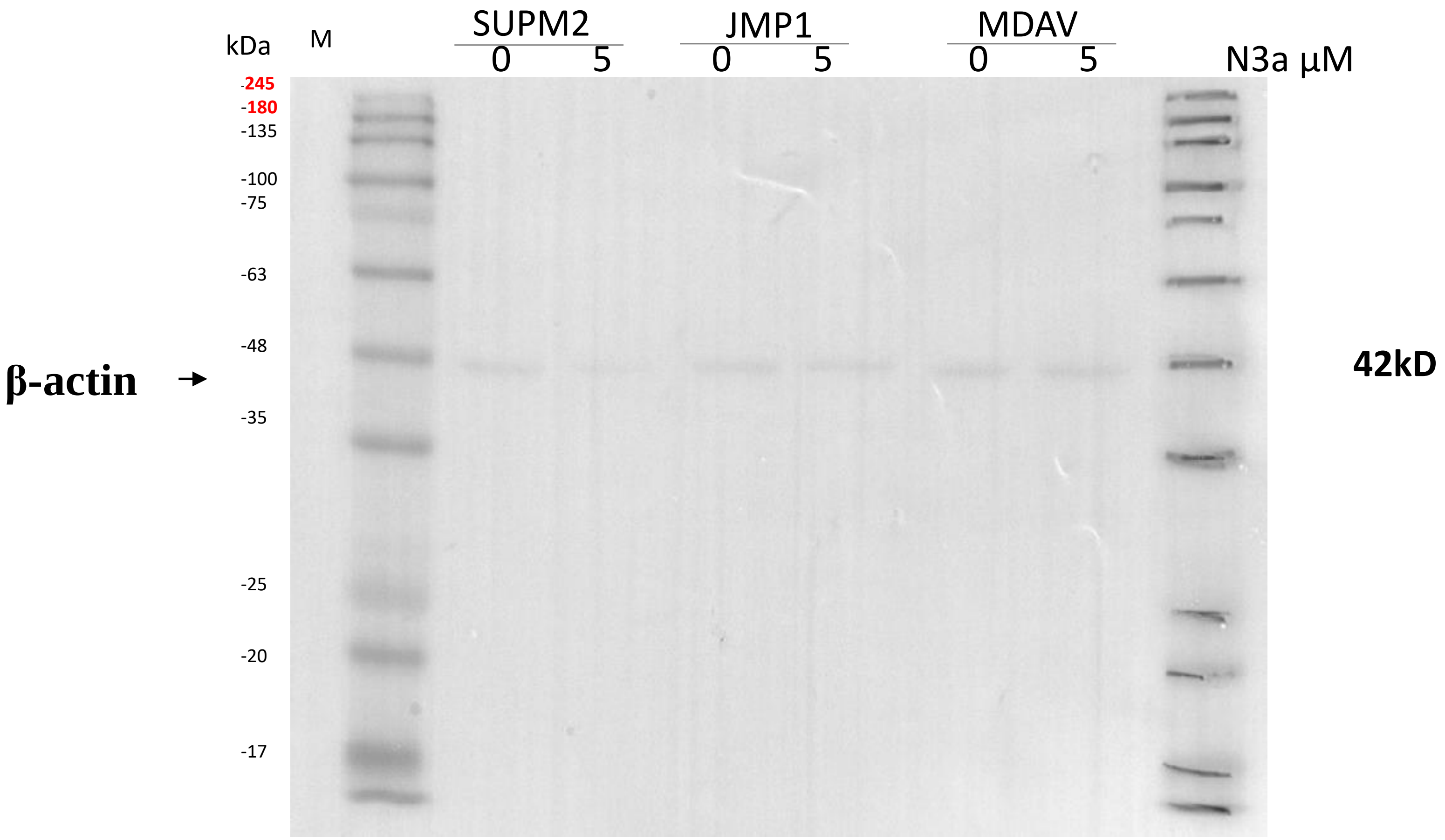

Supplement: Supplementary file 1 [file cancers-15-03903-s001.zip › Raw data_Antibodies PSatha et al. 2023.pdf]
