# Supplementary figures and images for: Interruption of p53-MDM2 Interaction by Nutlin-3a in Human Lymphoma Cell Models Initiates a Cell-Dependent Global Effect on Transcriptome and Proteome Level
Source: Cancers (Basel). 2023 Jul 31;15(15):3903. doi: 10.3390/cancers15153903 (PMC10417430; doi:10.3390/cancers15153903)

**A**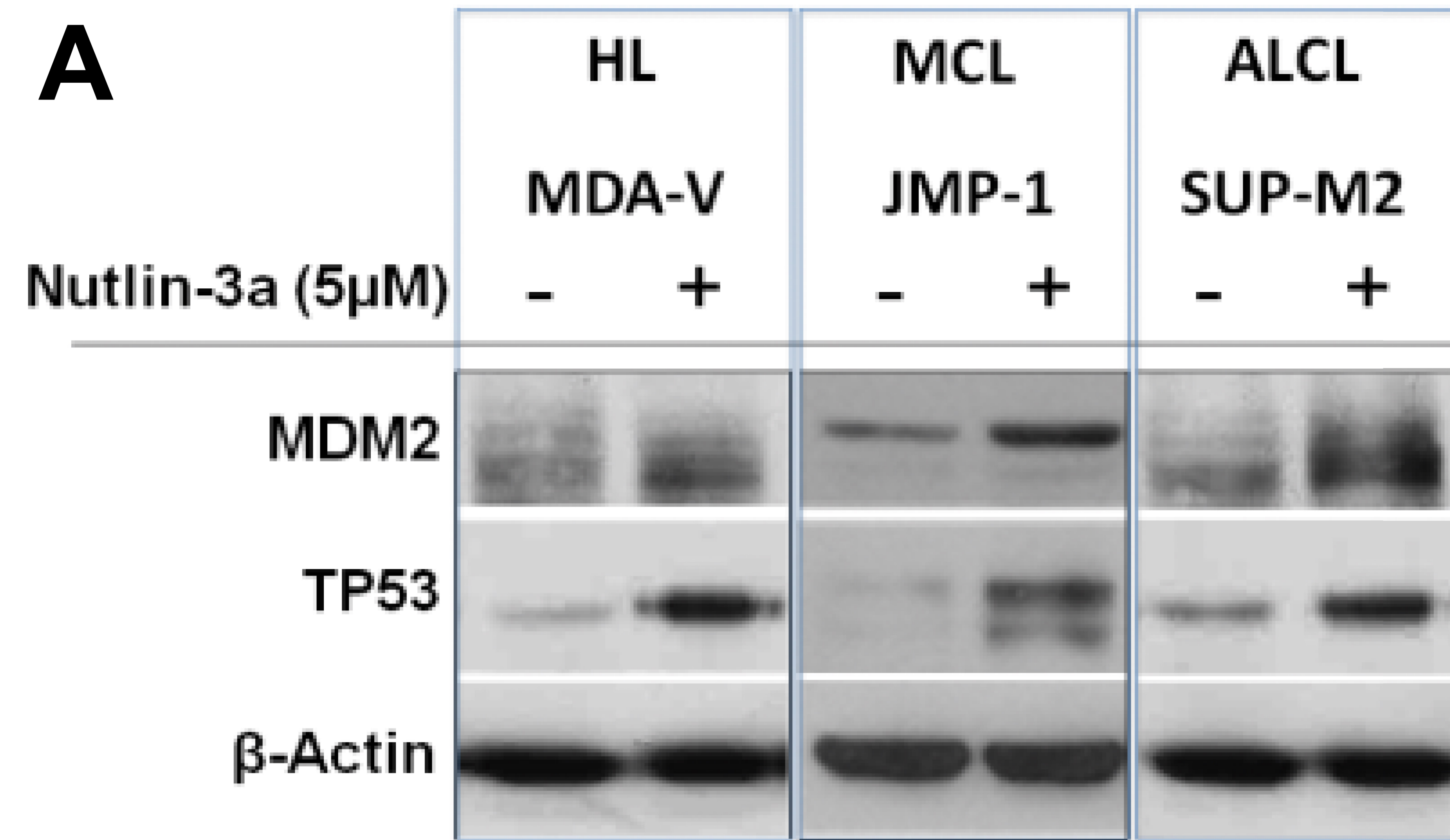**B**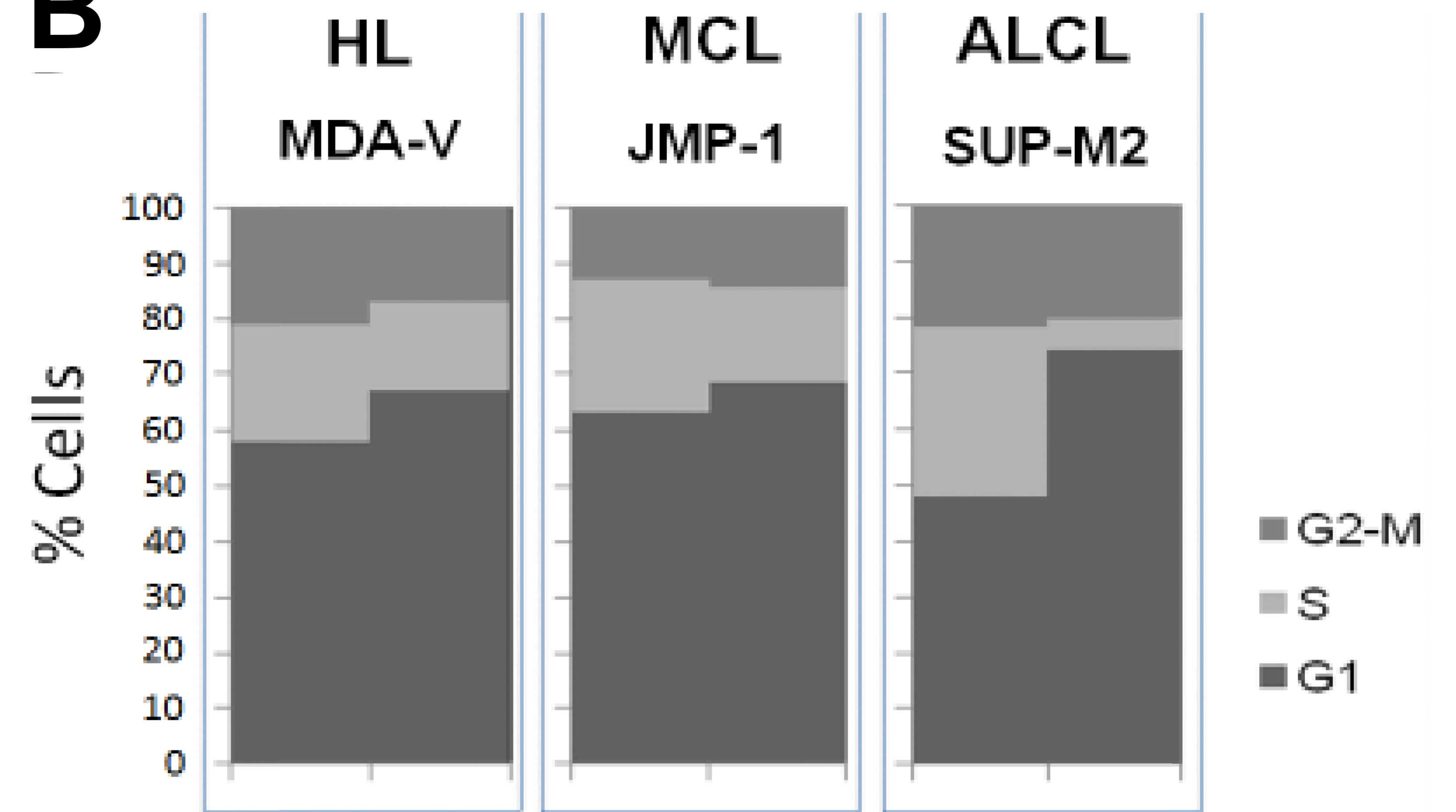**C**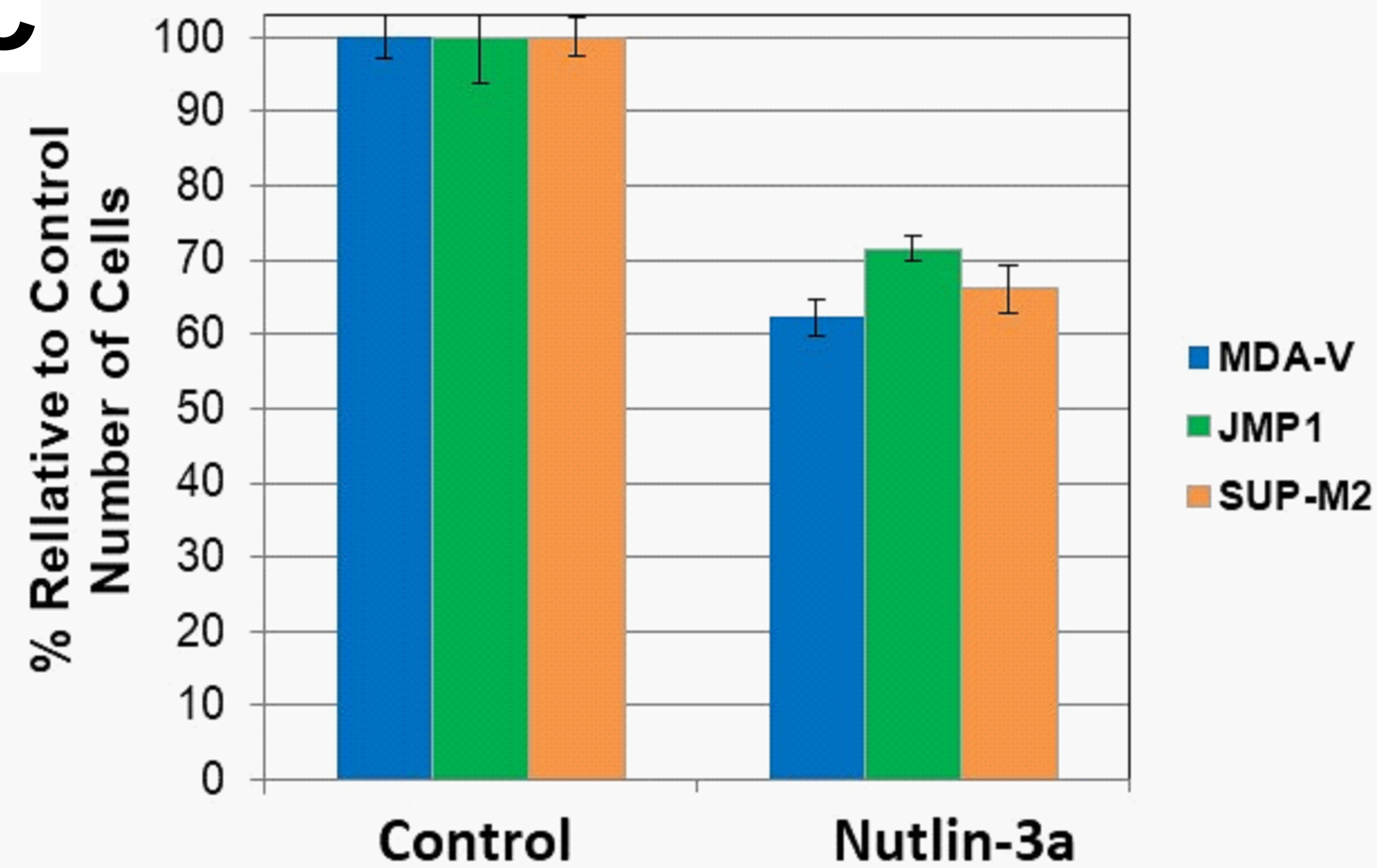**D**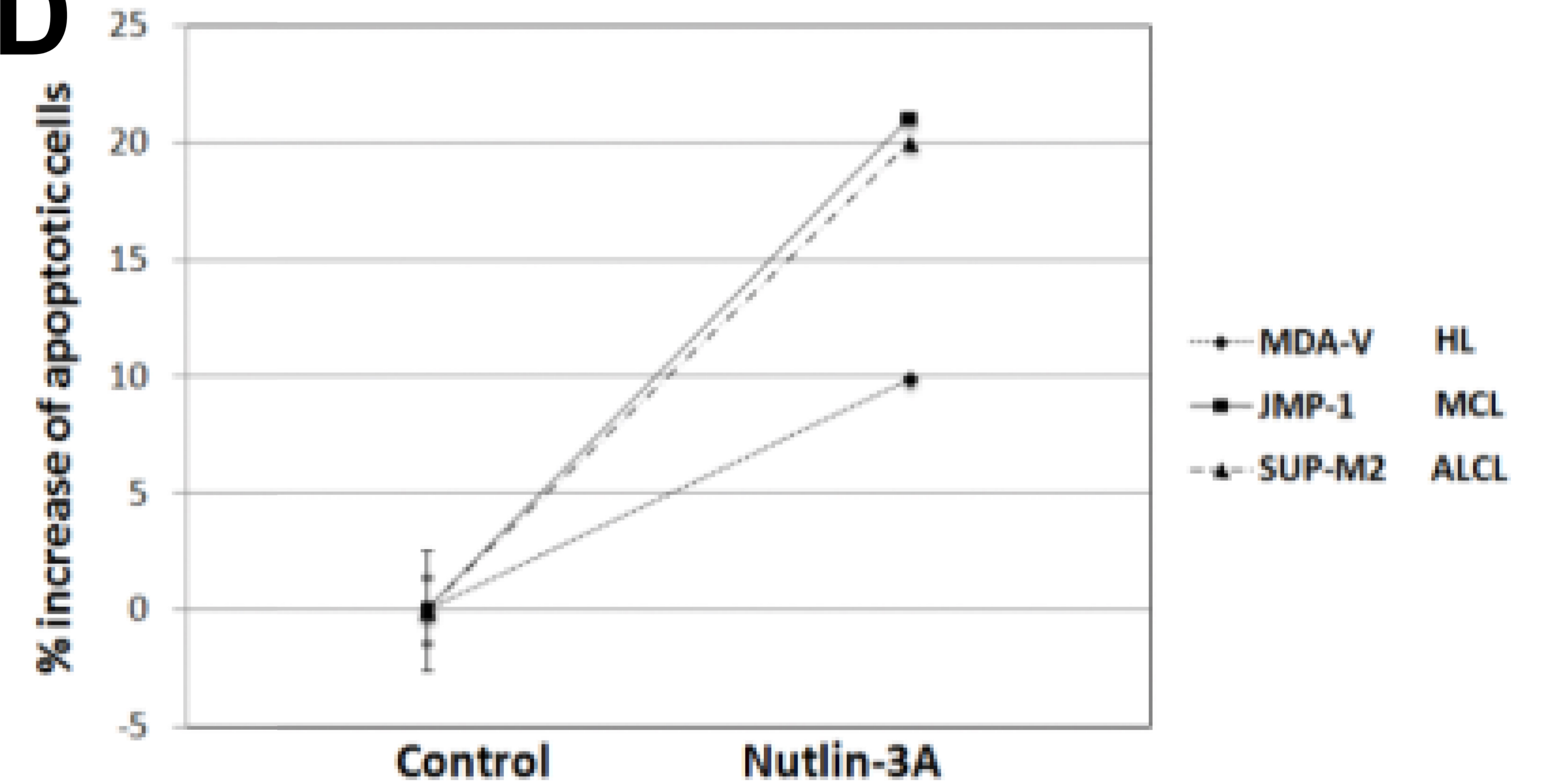

Supplement: Supplementary file 1 [file cancers-15-03903-s001.zip › Figure S10_N3a_Cell_viability_apoptosis new.pdf]

Figure S11: Validation of selected proteins in ALCL, HL and MCL human lymphoma cells. Psatha et al. 2023

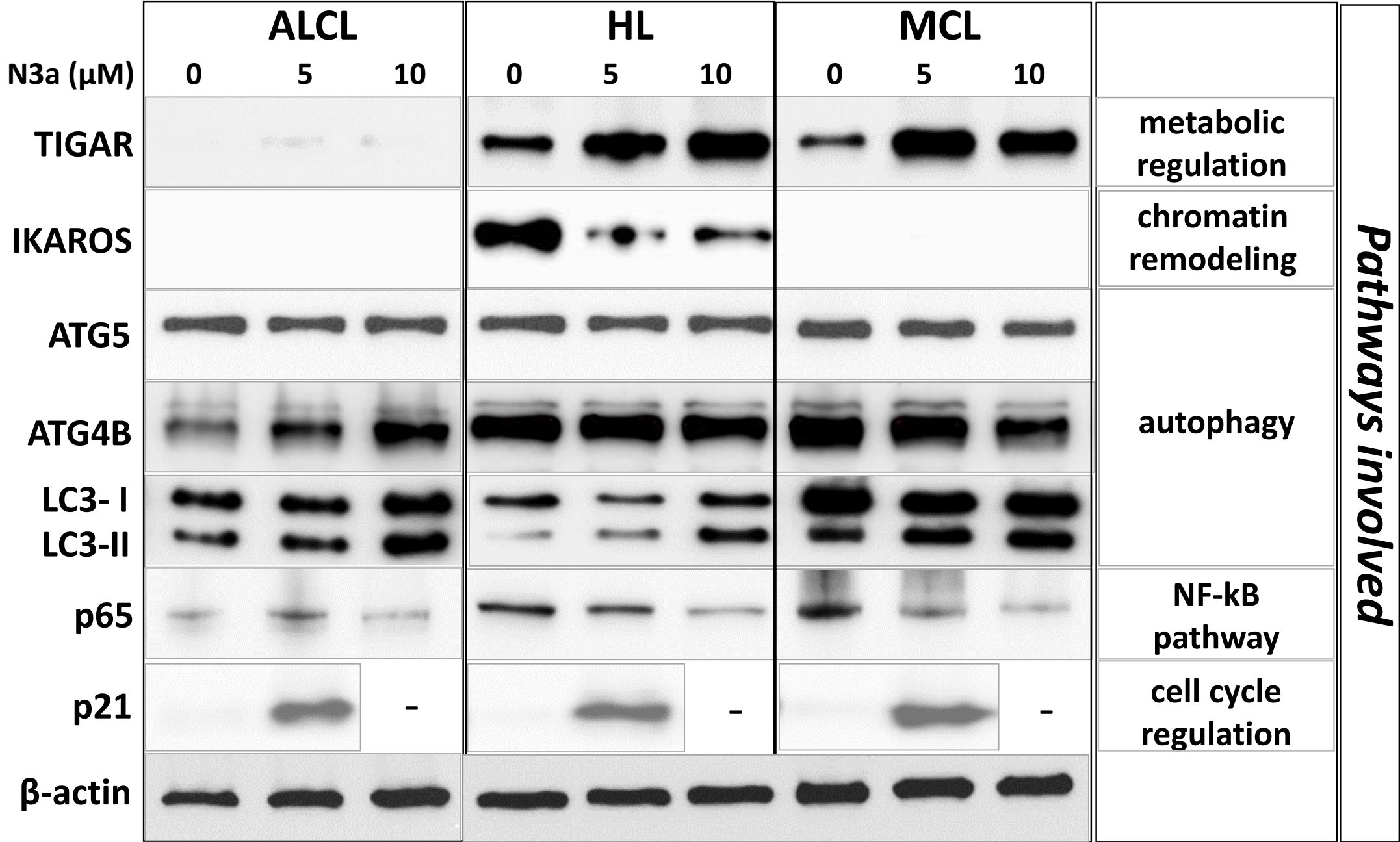

Supplement: Supplementary file 1 [file cancers-15-03903-s001.zip › Figure S11 Additional Western Blots.pdf]

**ALCL**

**HL**

**MCL**

**control**

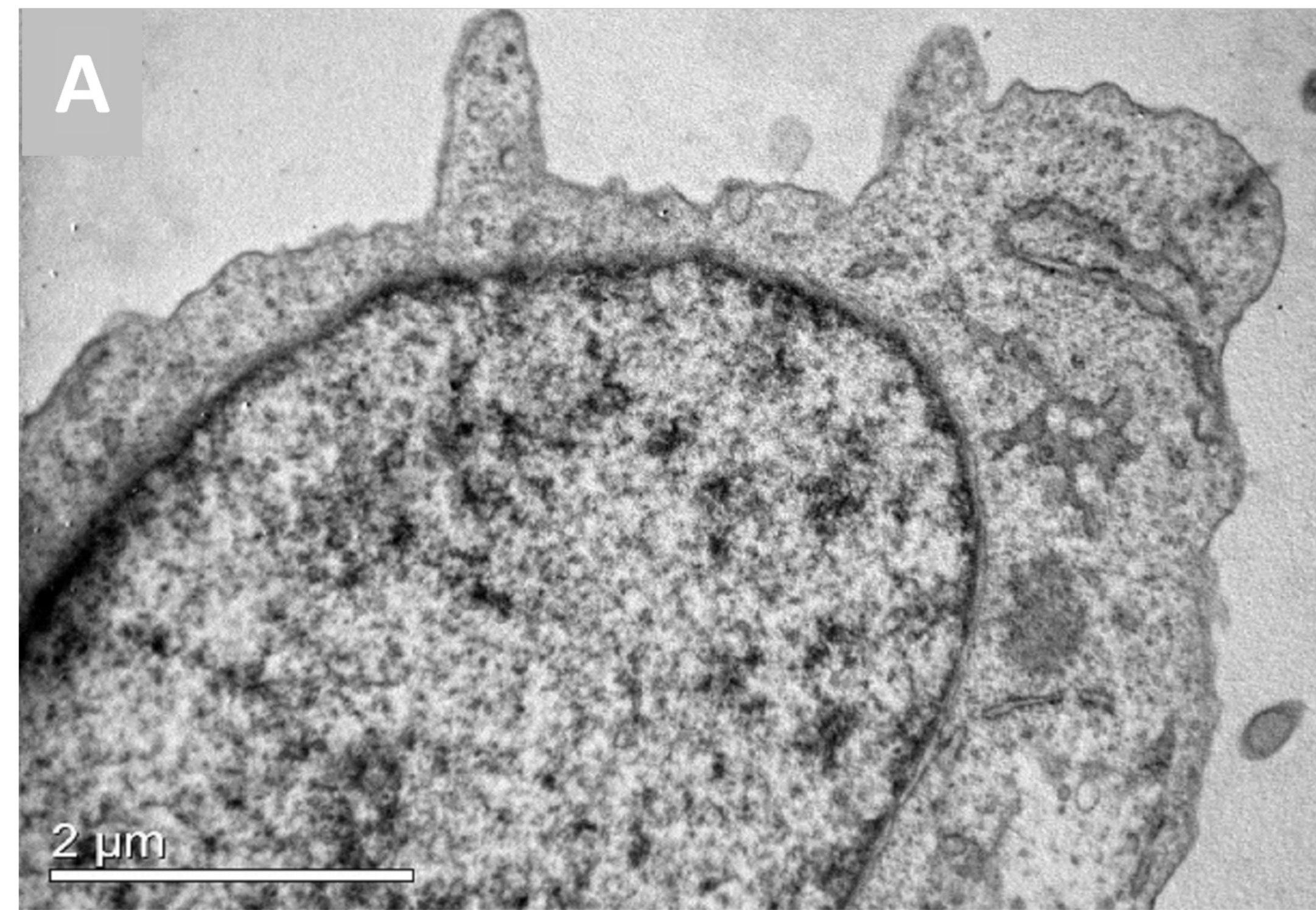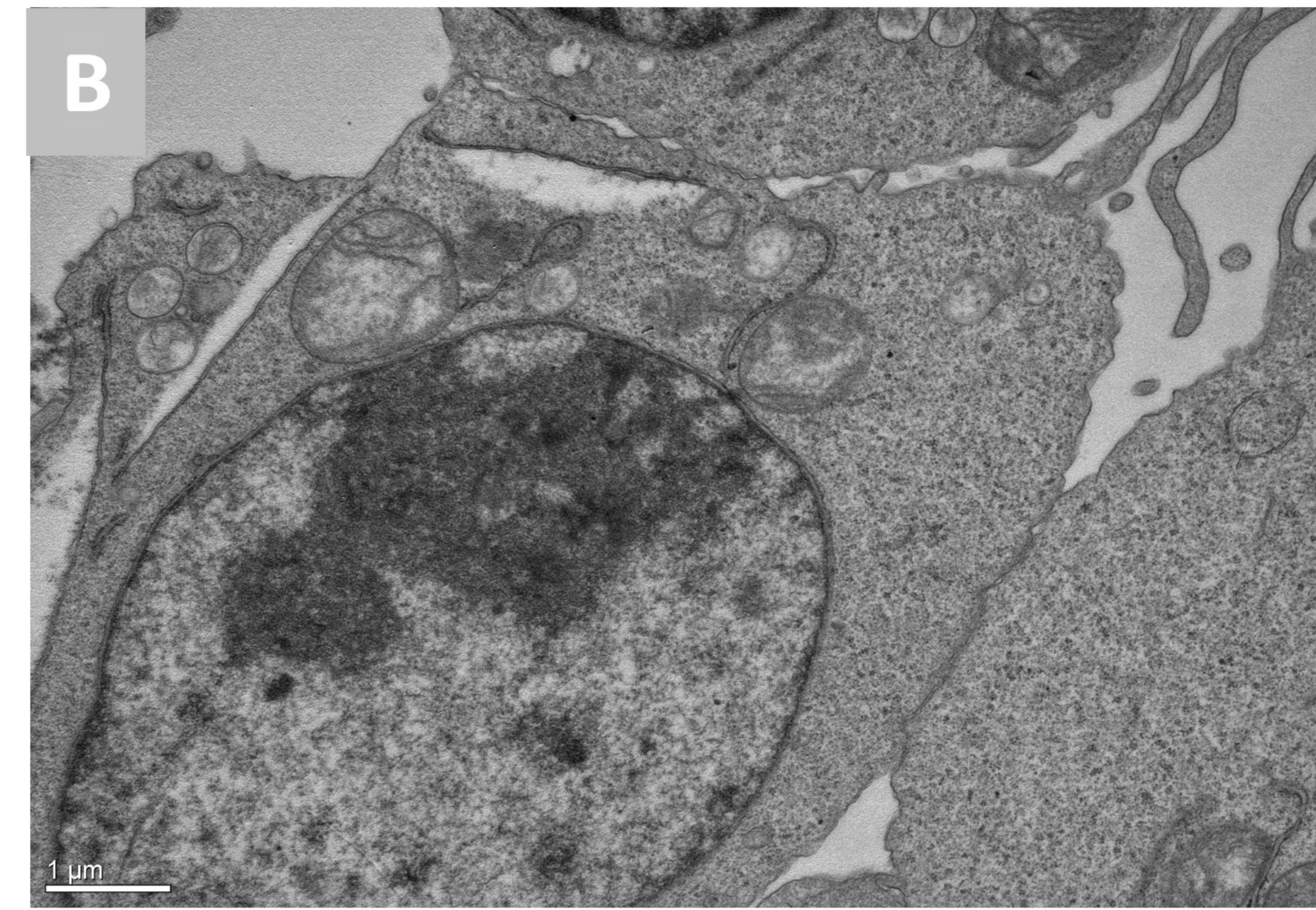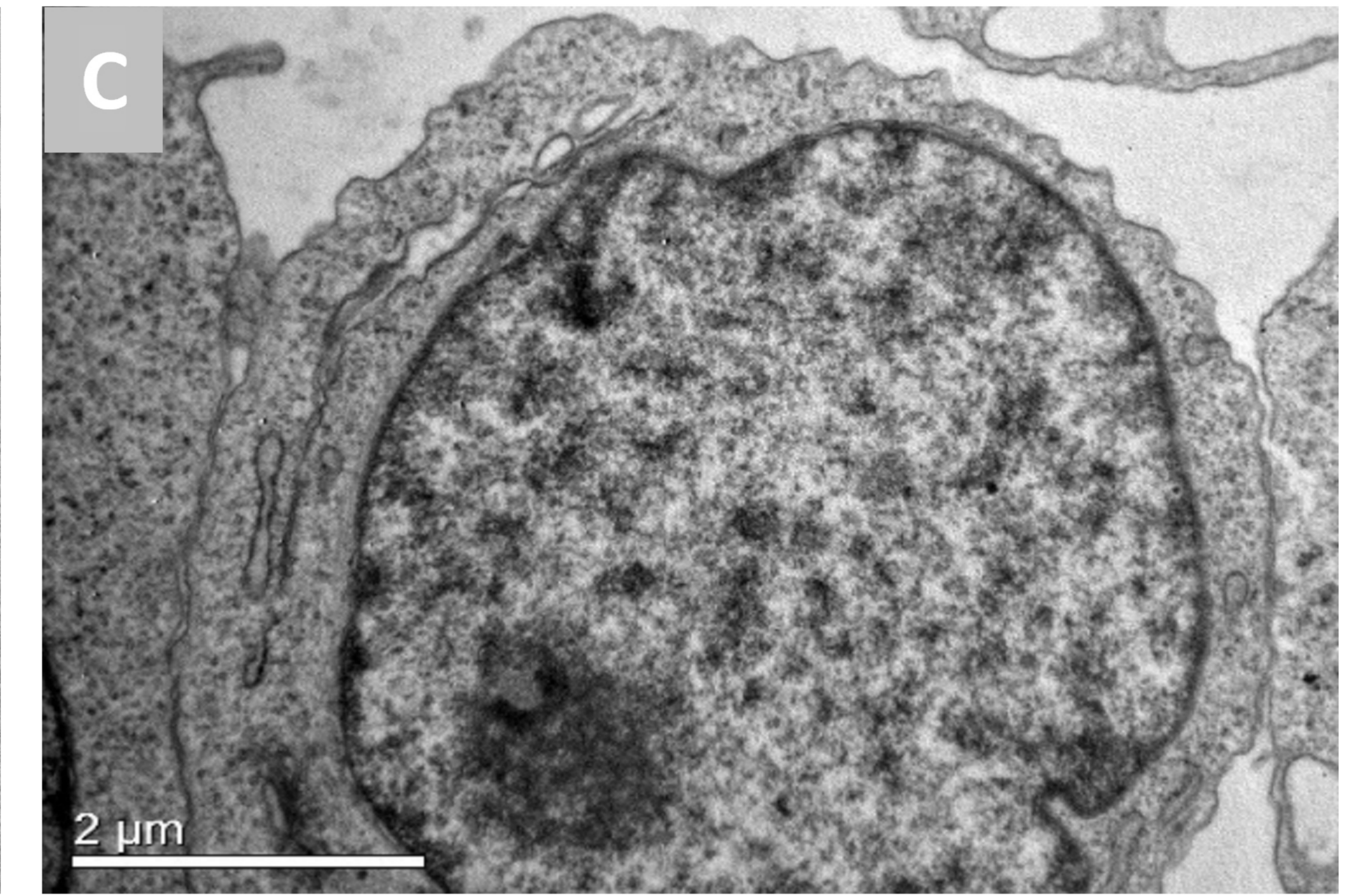

***apoptosis***

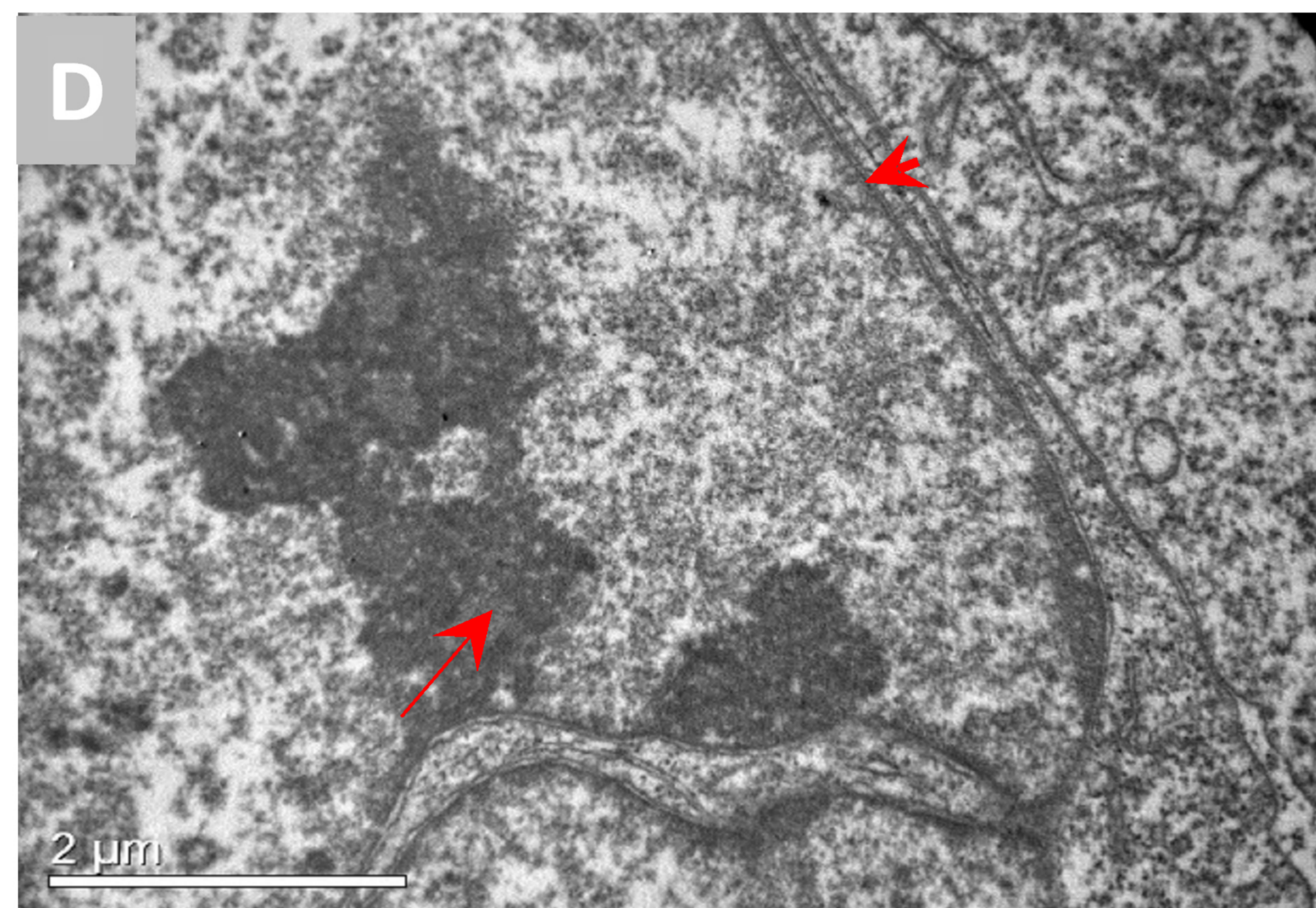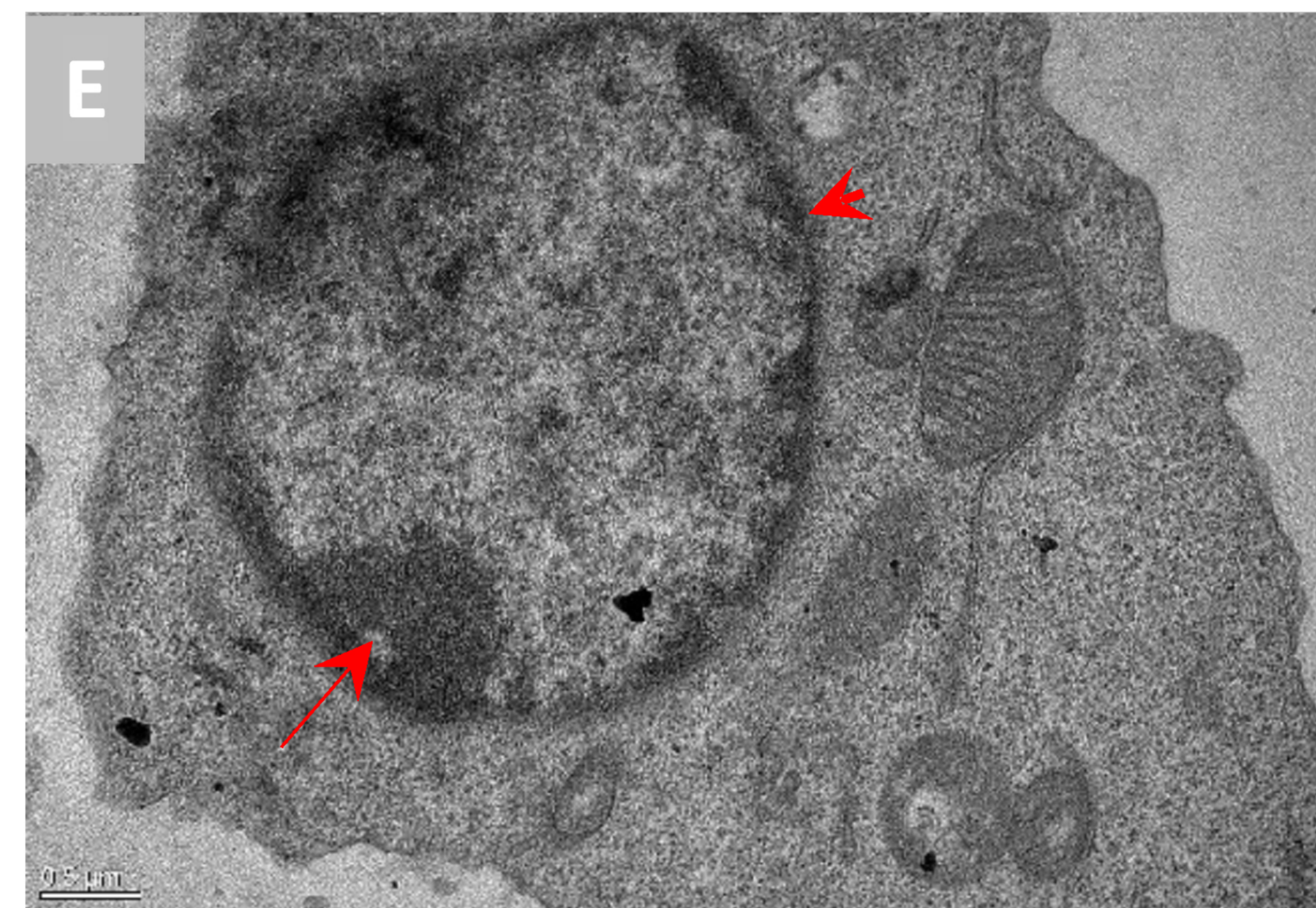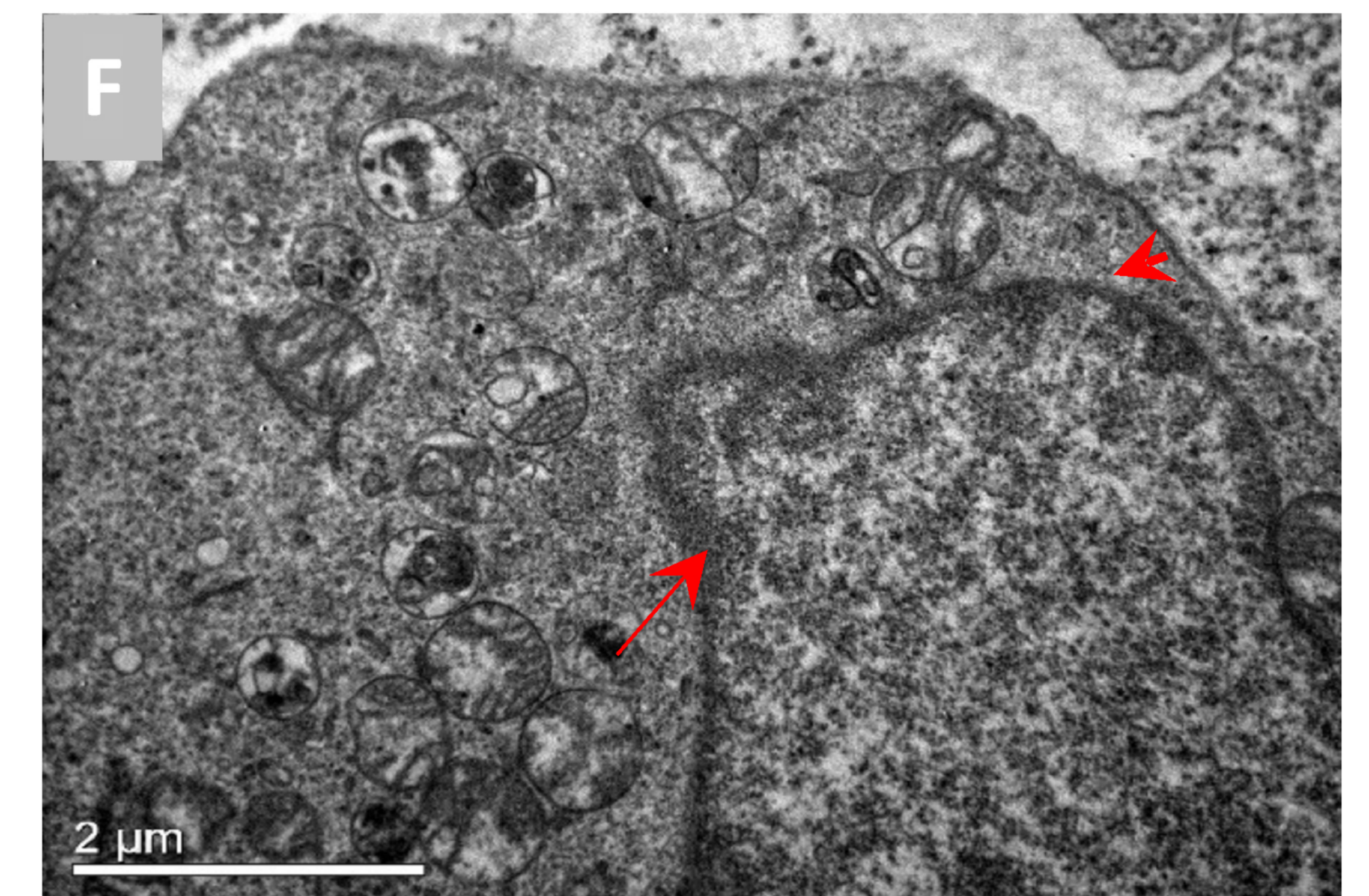

**+N3a**

***autophagy***

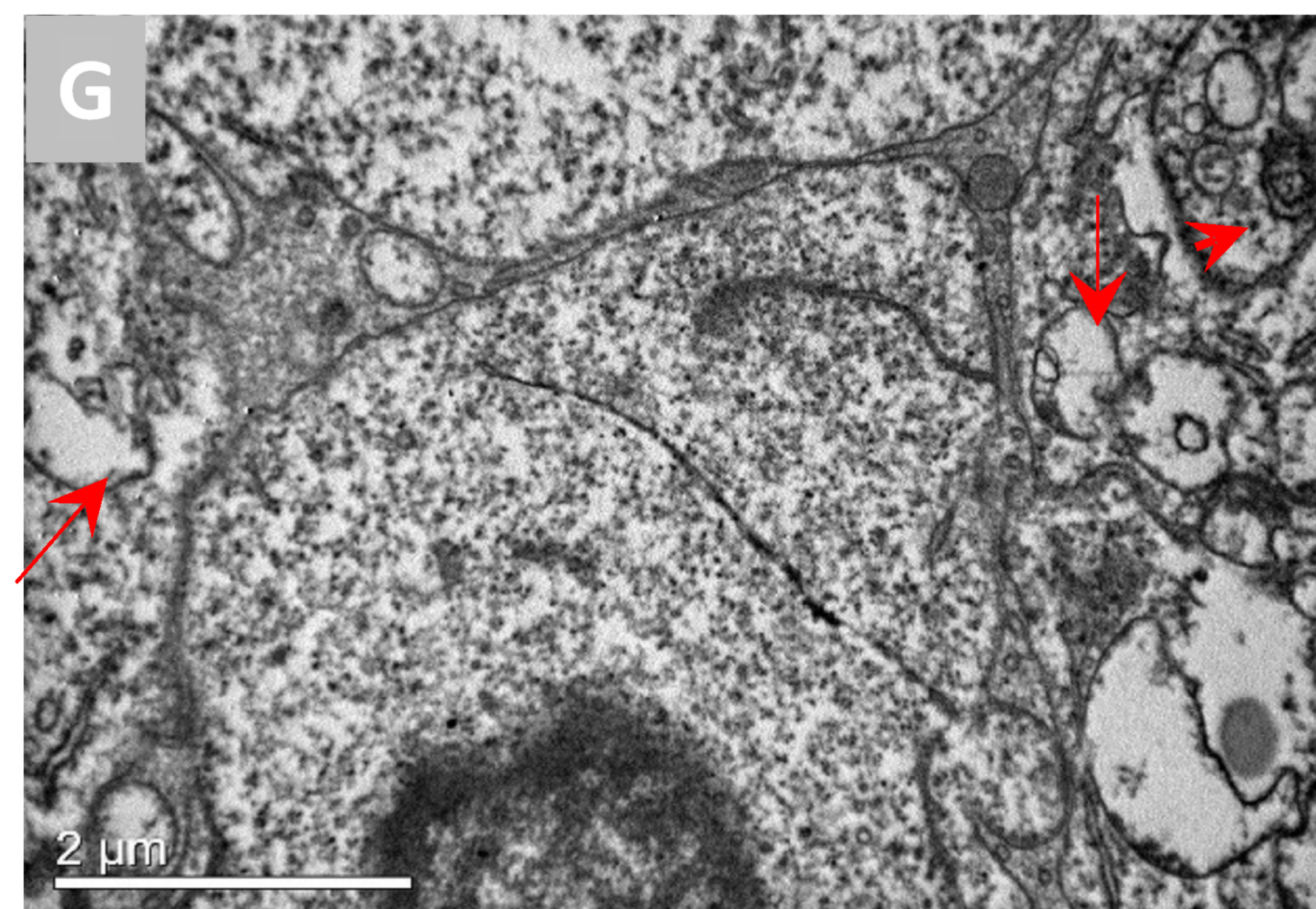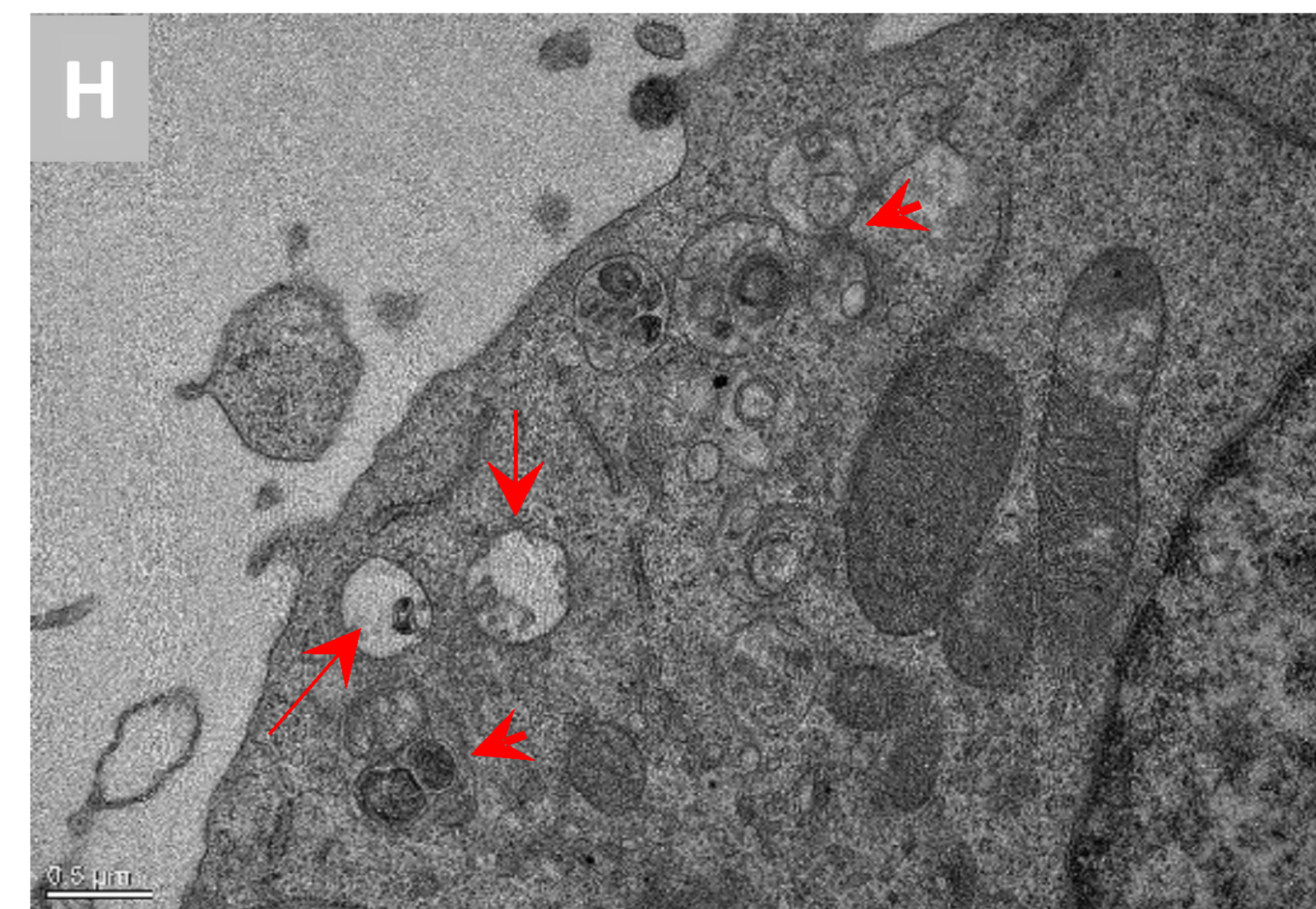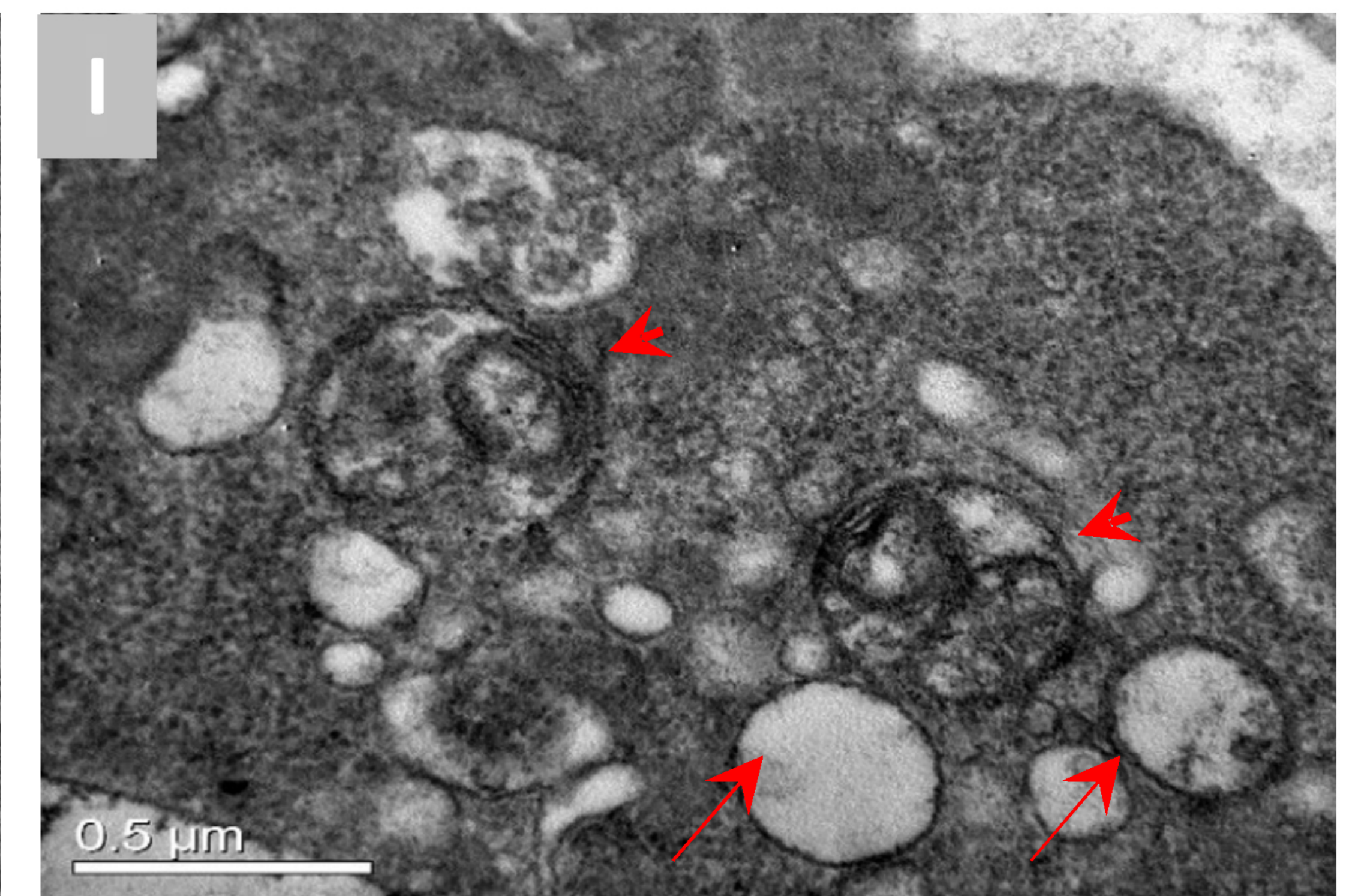

Supplement: Supplementary file 1 [file cancers-15-03903-s001.zip › Figure S12_TEM.pdf]

# Mitotracker Intensity of live cells

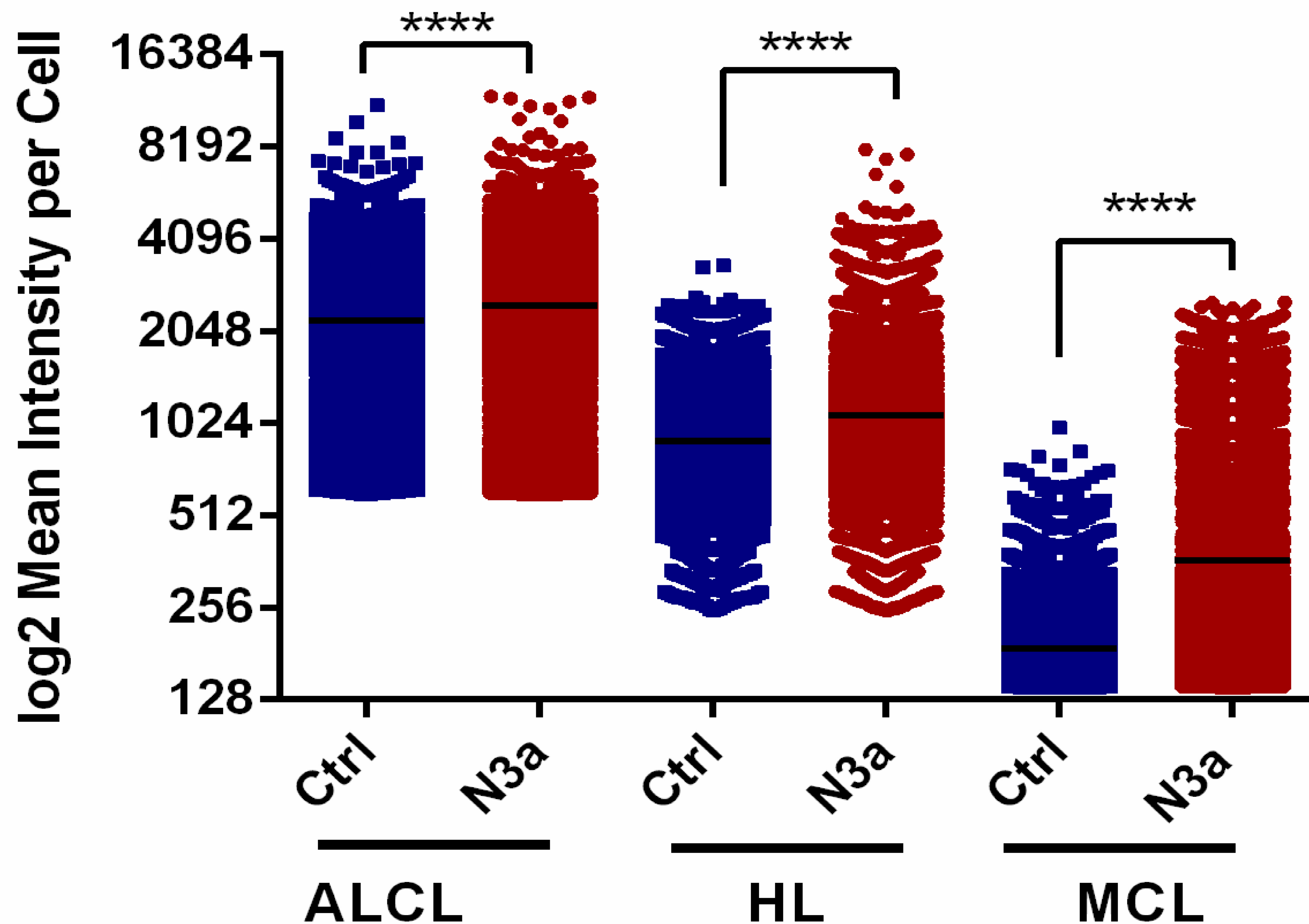

Supplement: Supplementary file 1 [file cancers-15-03903-s001.zip › Figure S13_HCI_Mitotracker.pdf]

FigS1: Overview of the deregulated omics profiling of N3a-affected lymphoma cells.. Psatha et al. 2023

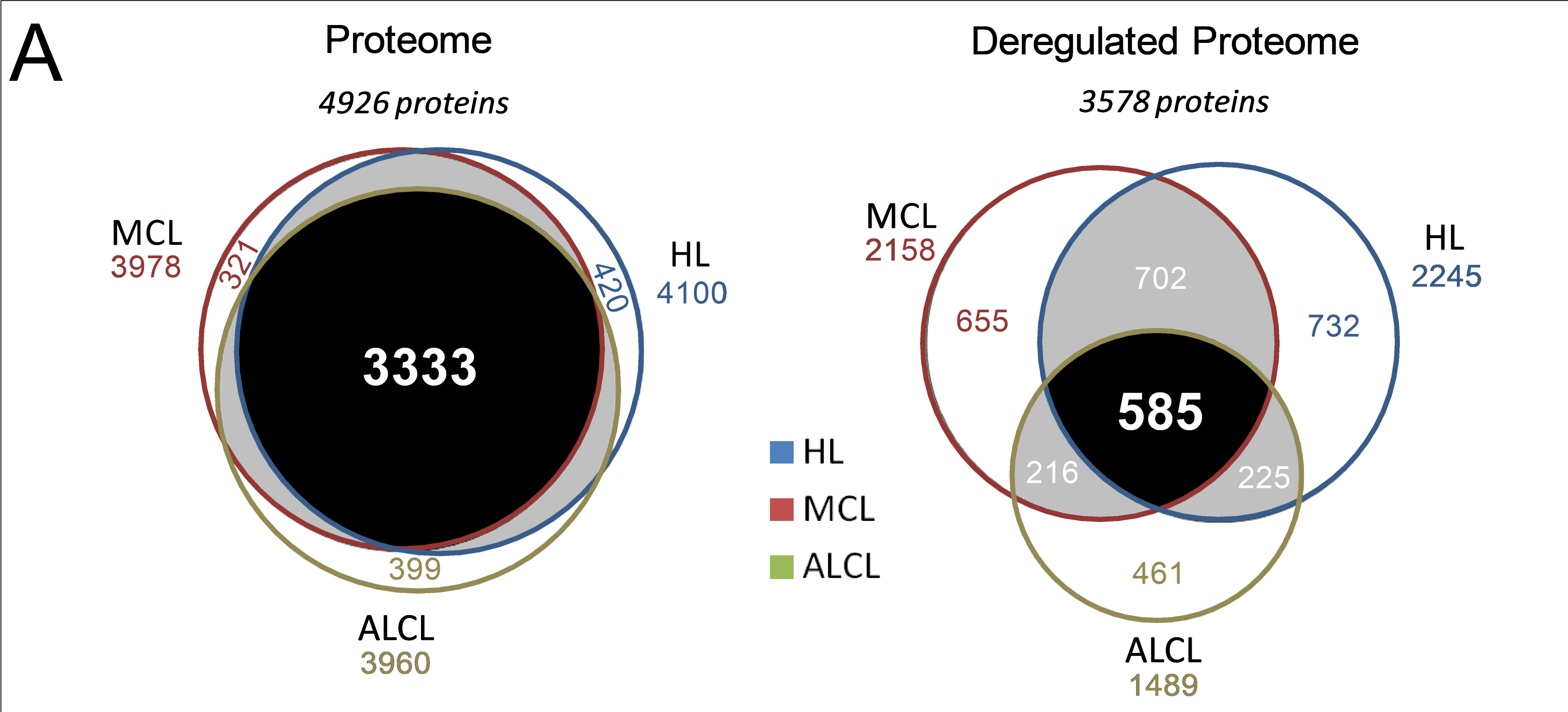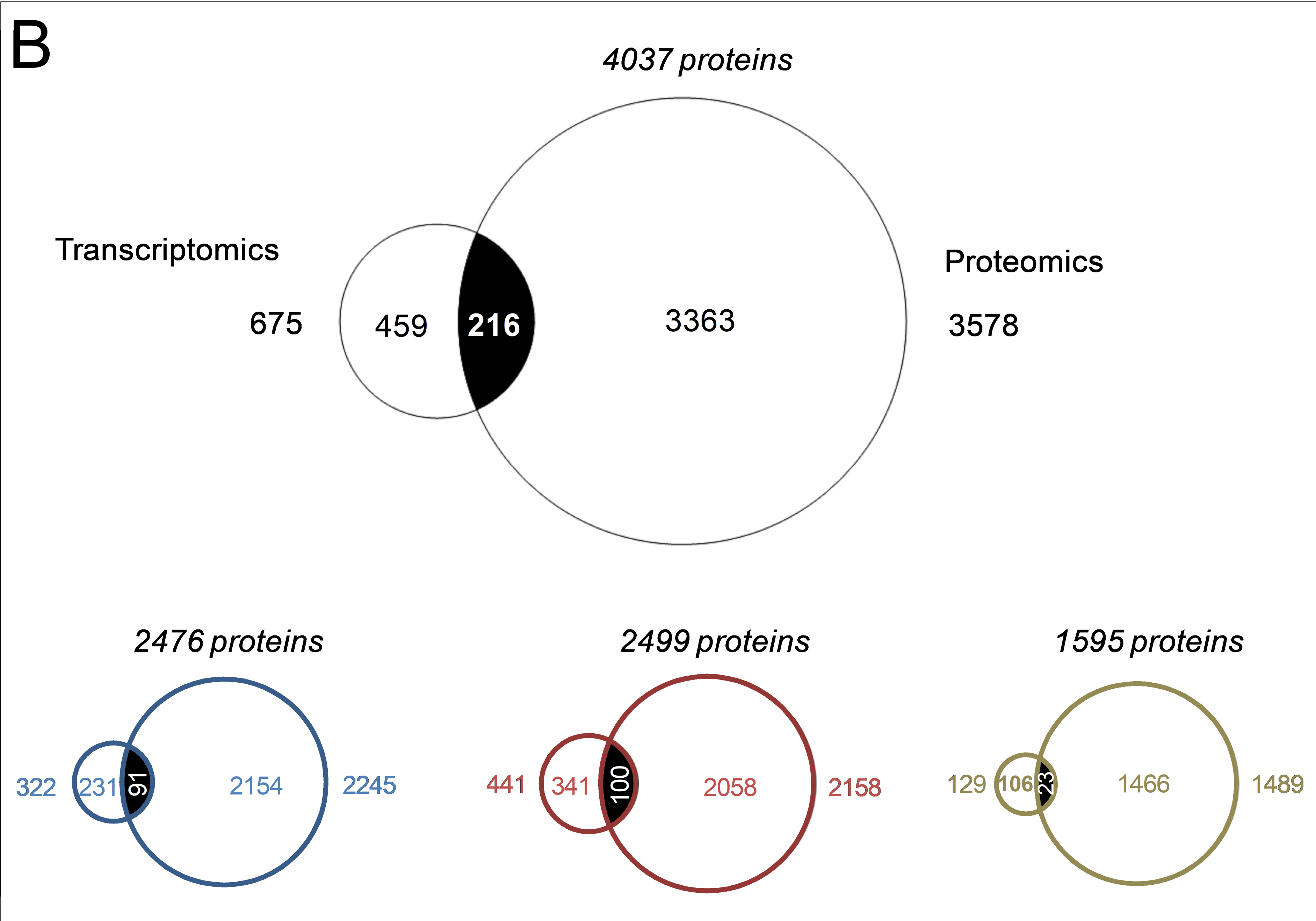

Supplement: Supplementary file 1 [file cancers-15-03903-s001.zip › Figure S1_Overview of the omics data.pdf]

**Fig S02 Common deregulated mRNAs in three lymphoma types. Psatha et. al 2023**

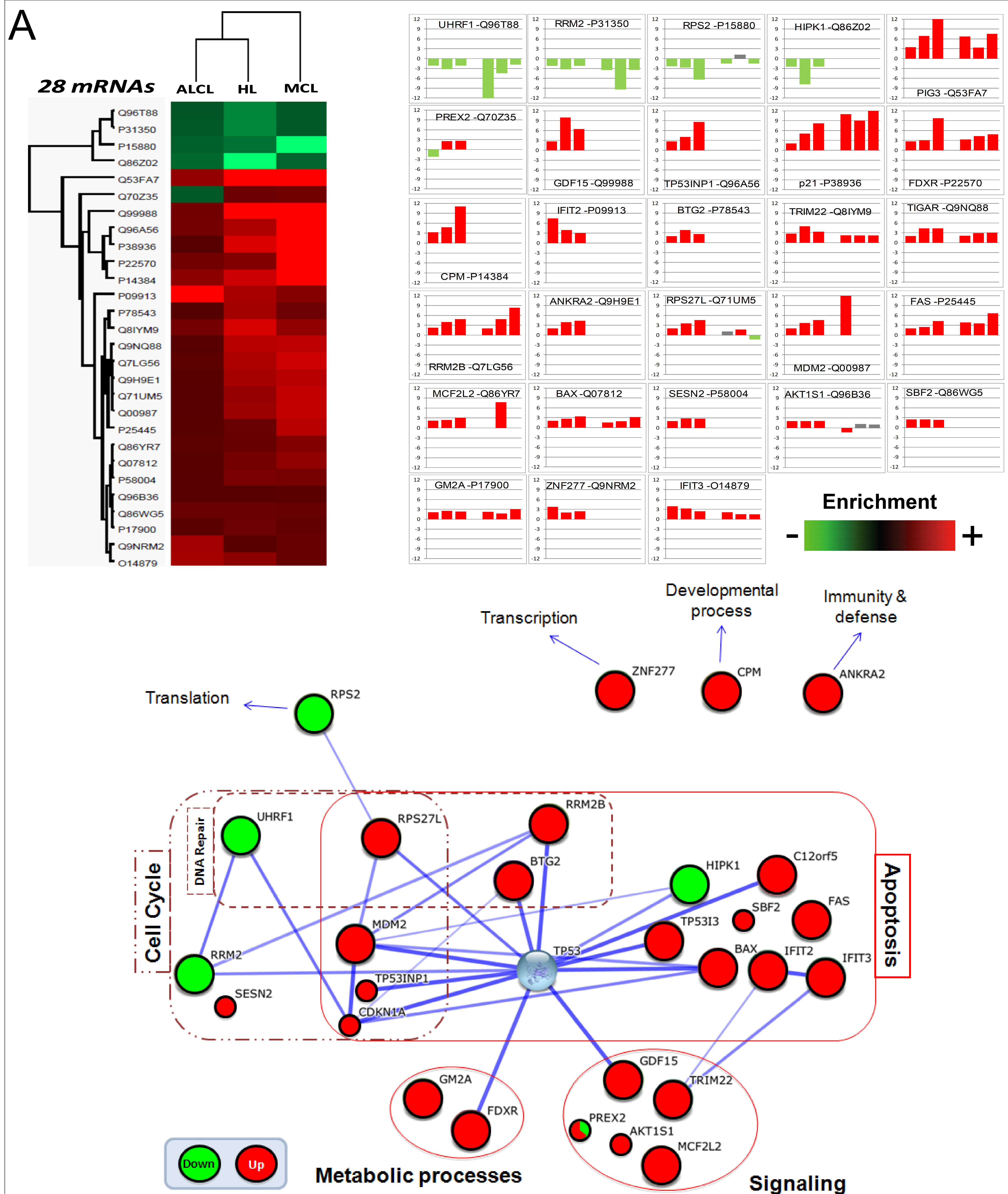

Supplement: Supplementary file 1 [file cancers-15-03903-s001.zip › Figure S2_mRNA_commo_der_genes.pdf]

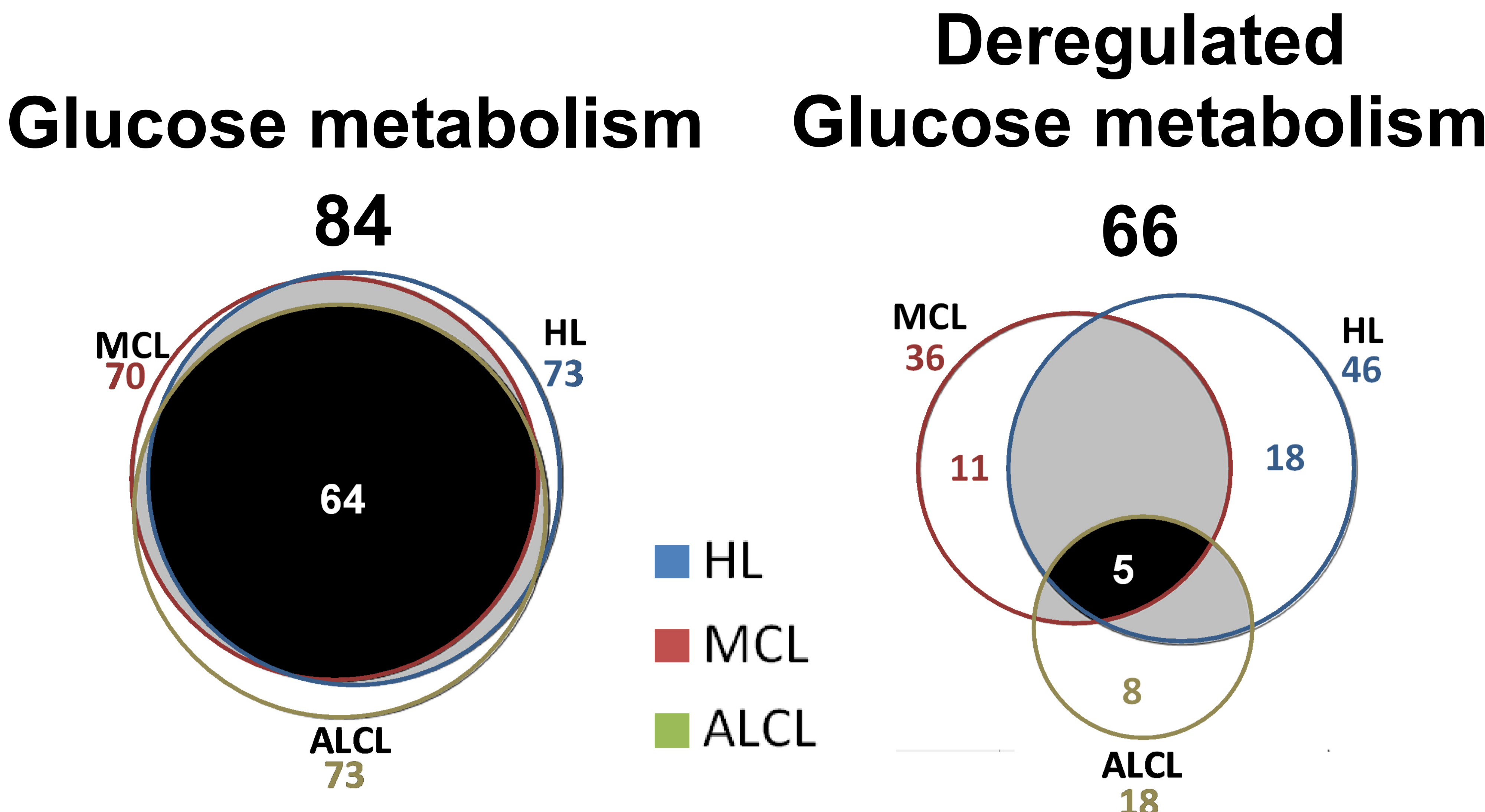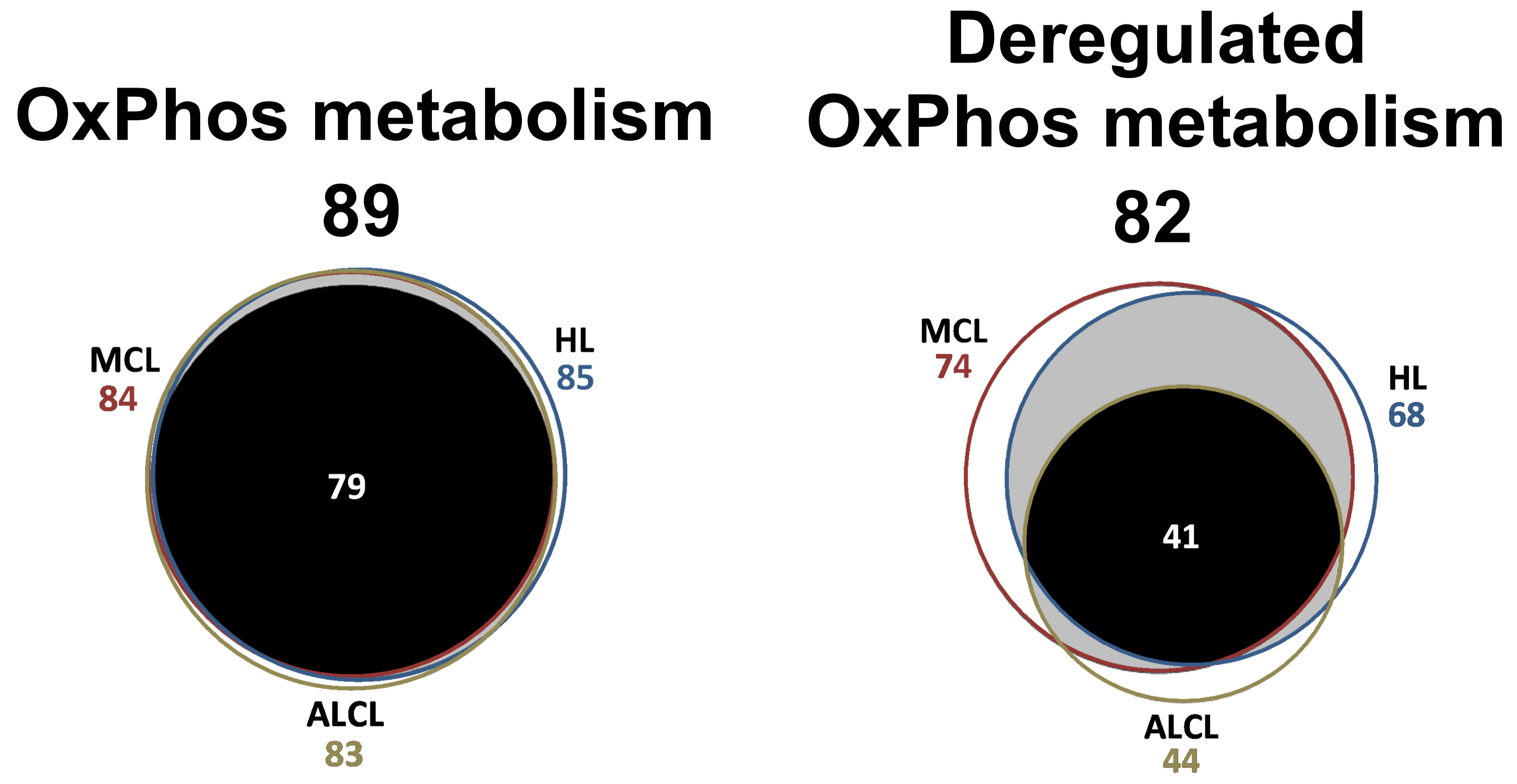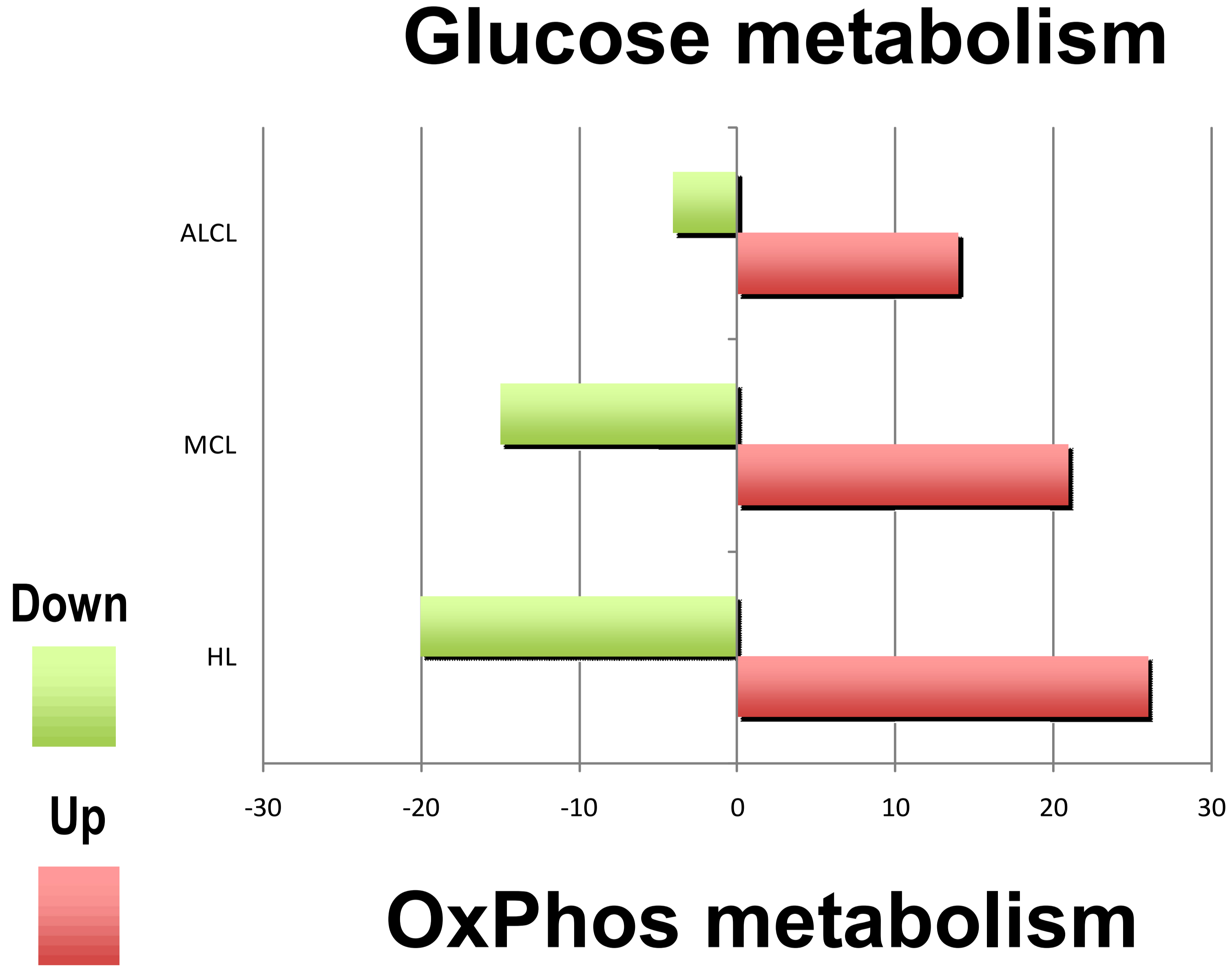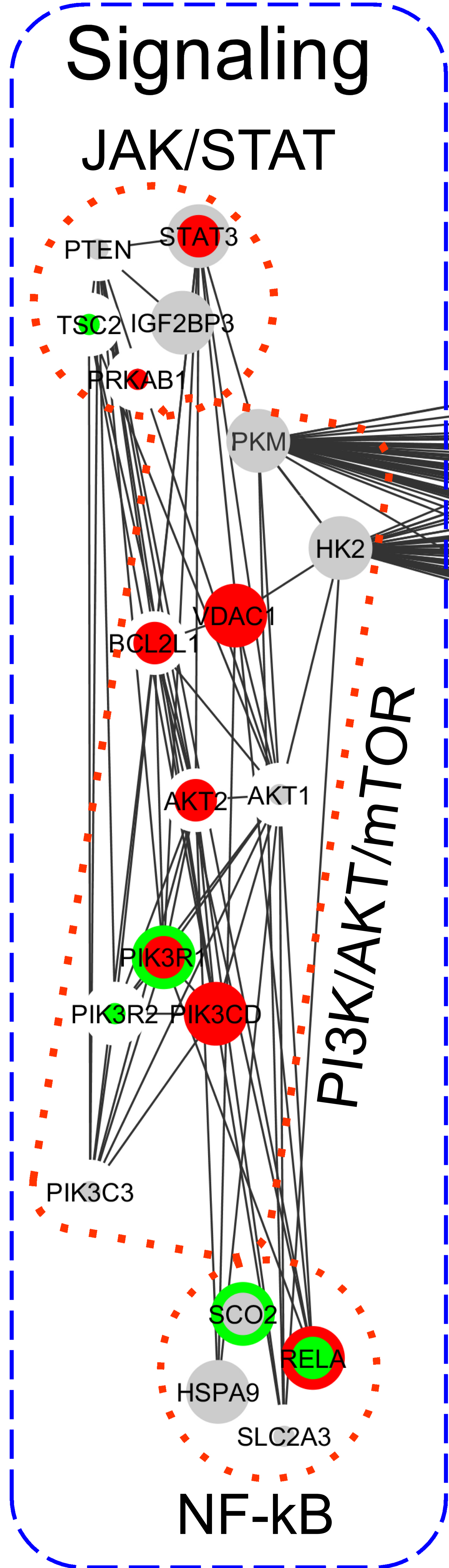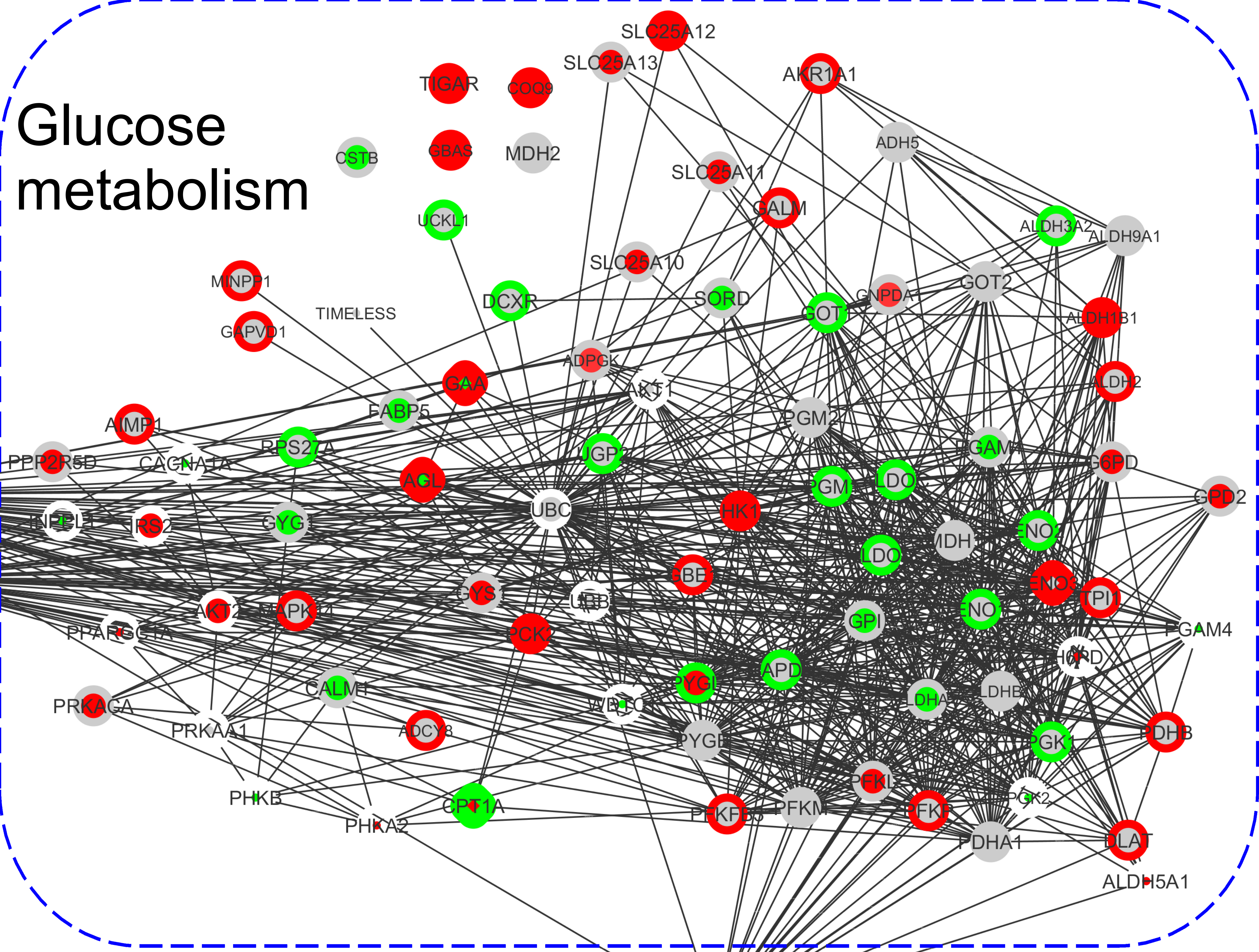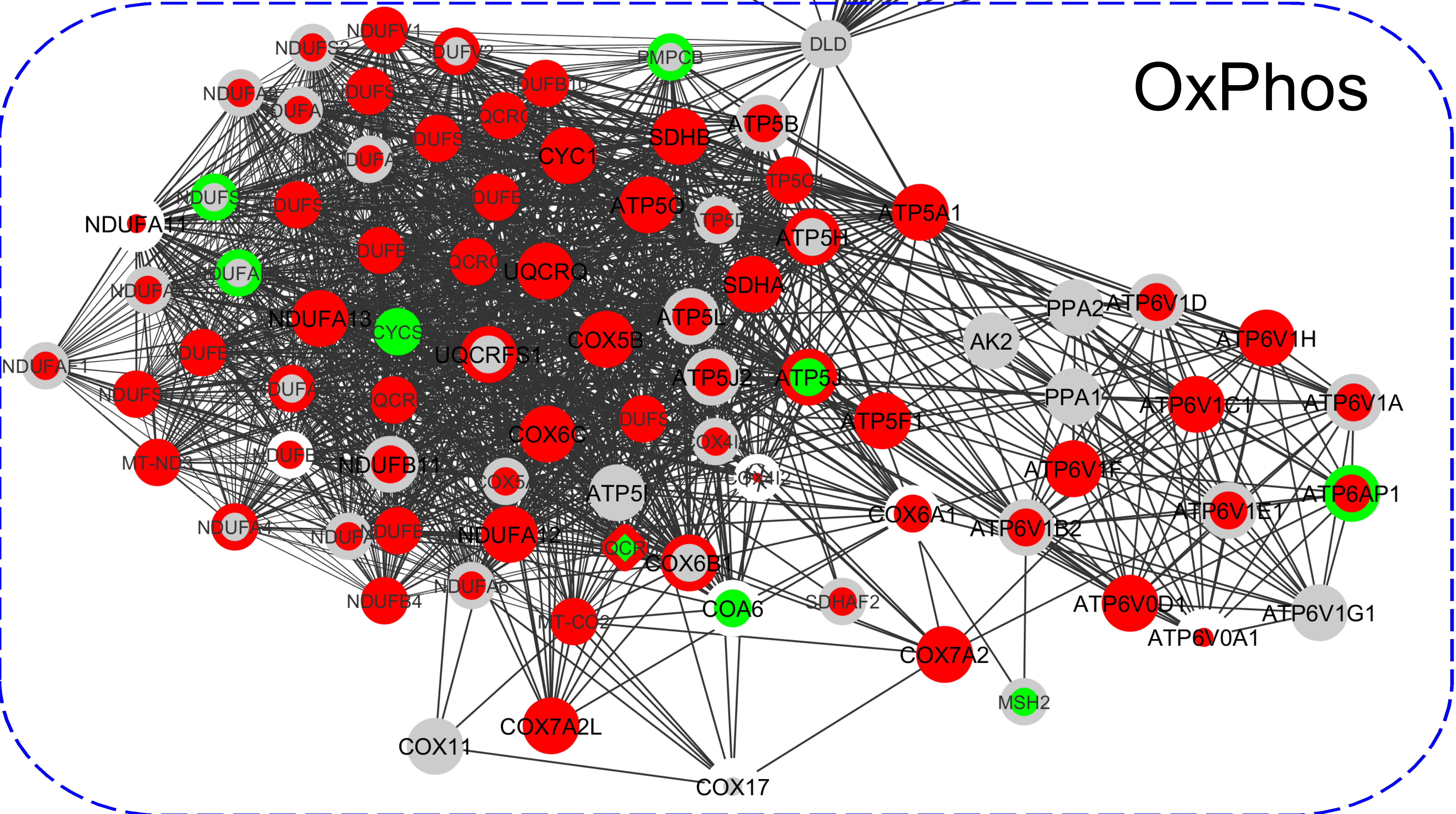

Supplement: Supplementary file 1 [file cancers-15-03903-s001.zip › Figure S6_Energy_metabolism.pdf]

# PI3K/AKT signaling pathway

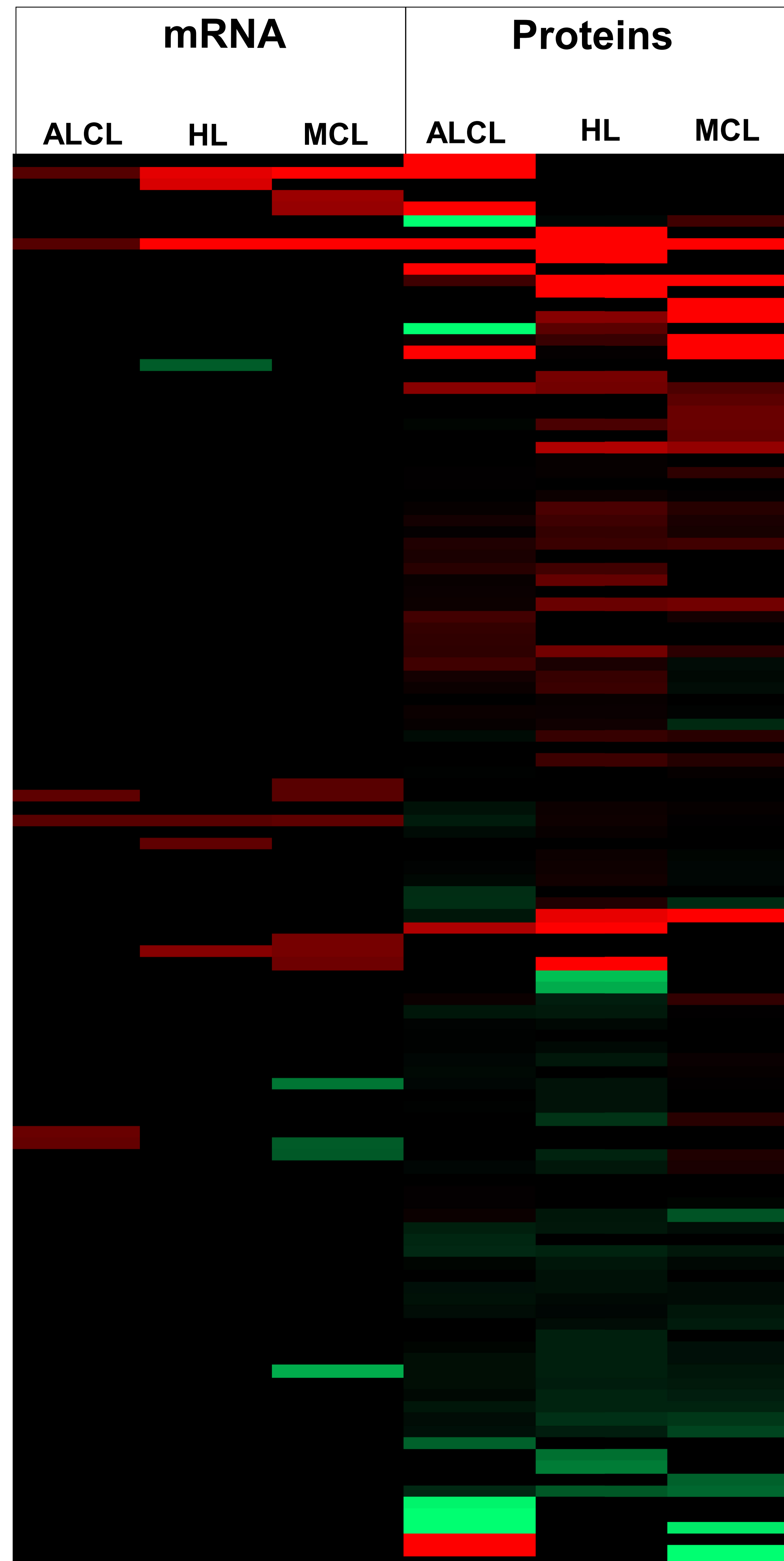[illegible]

Supplement: Supplementary file 1 [file cancers-15-03903-s001.zip › Figure S7_mTOR_HSR.pdf]

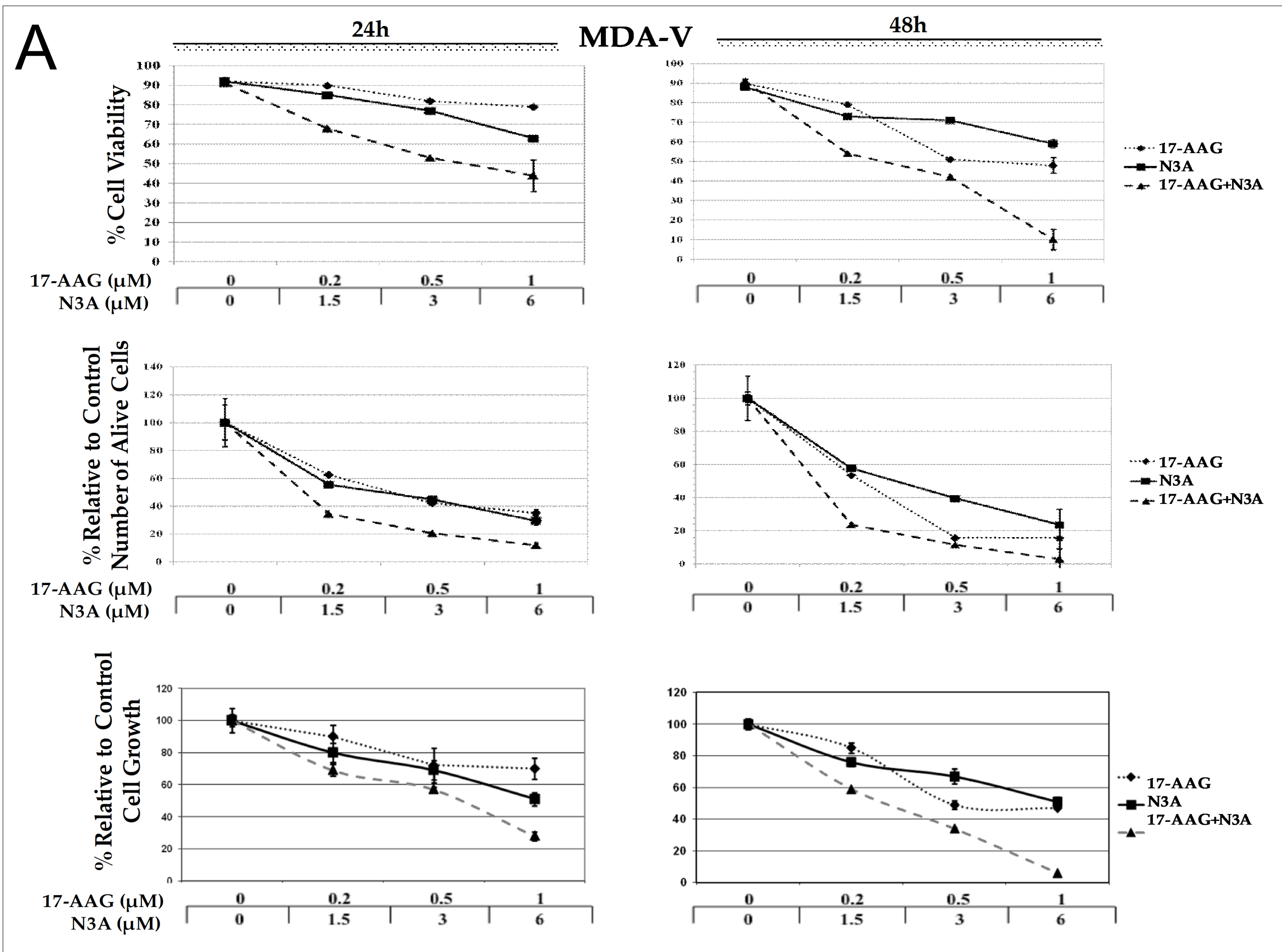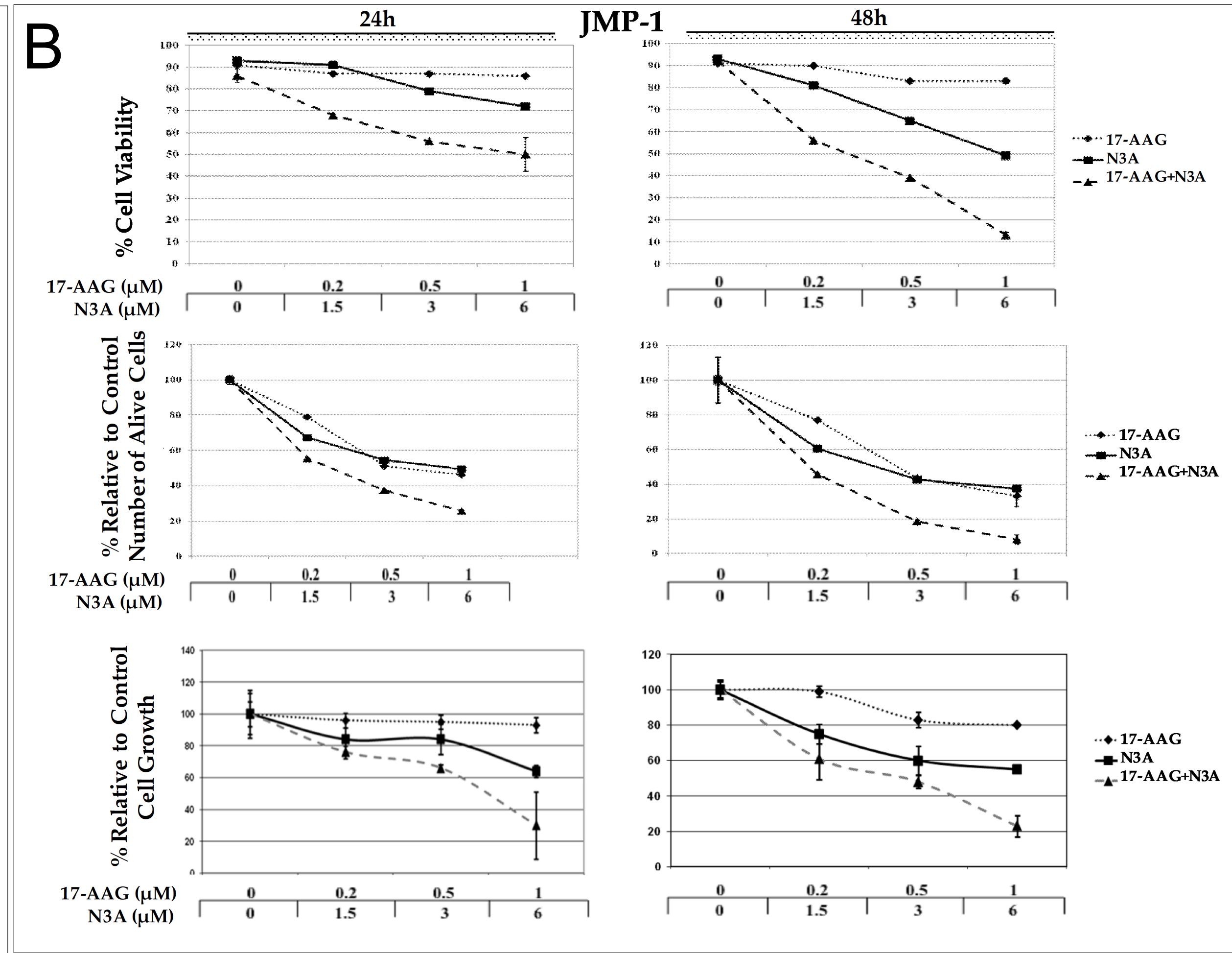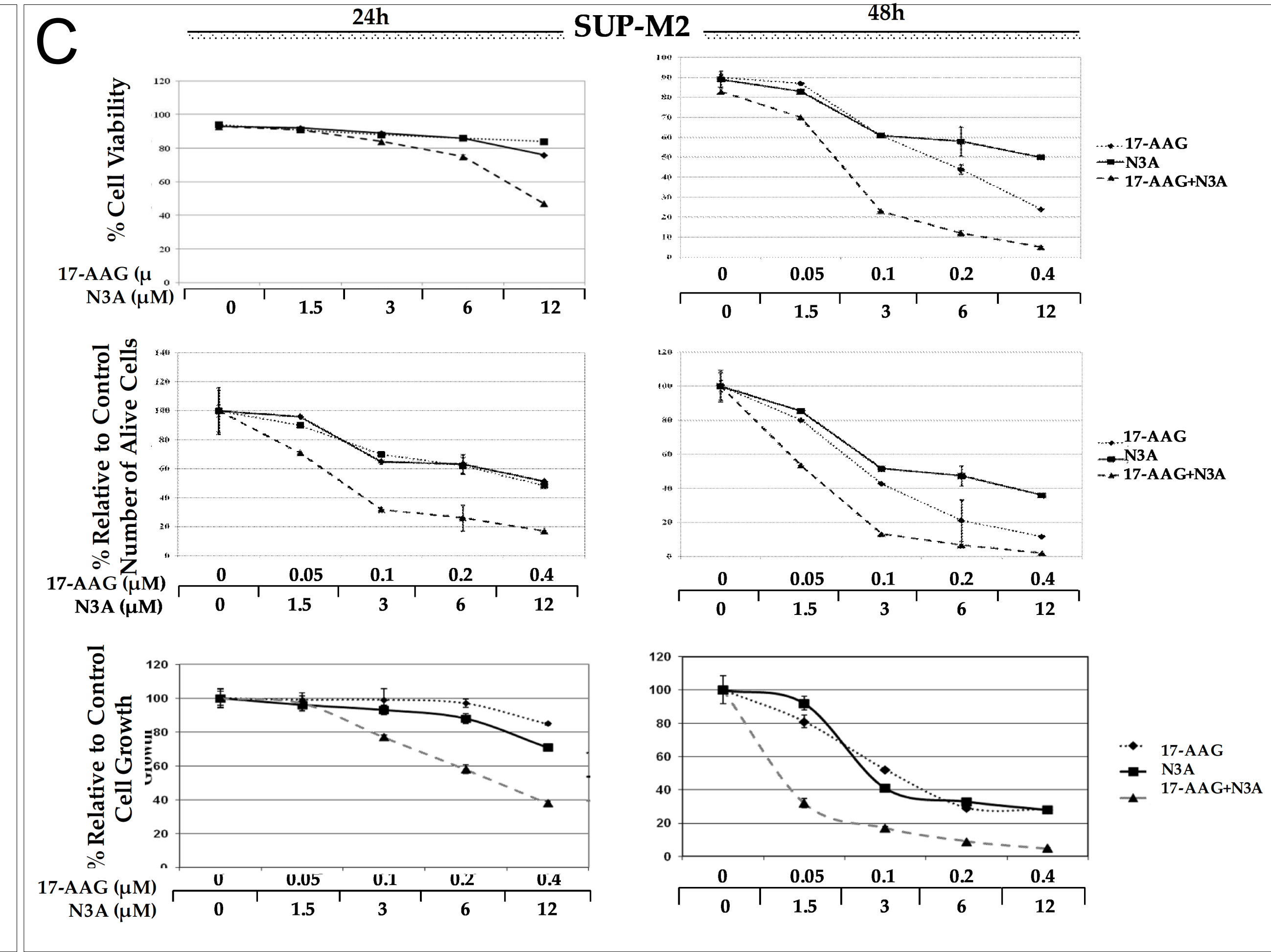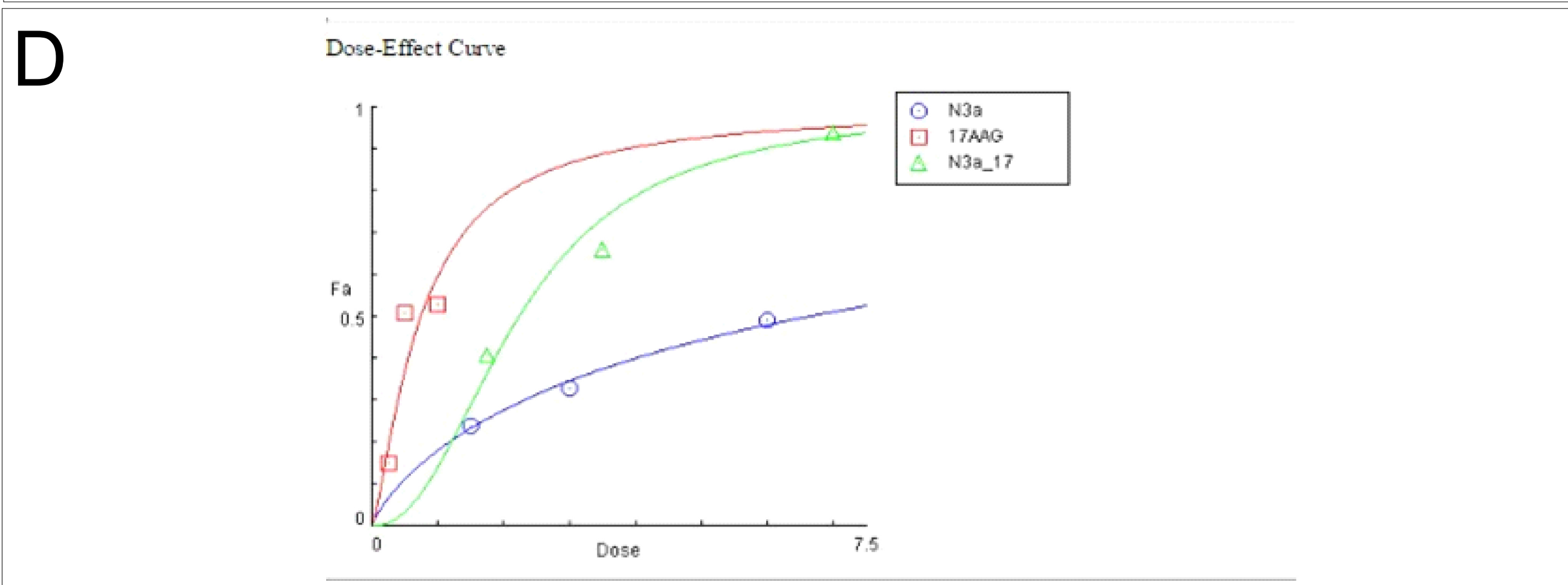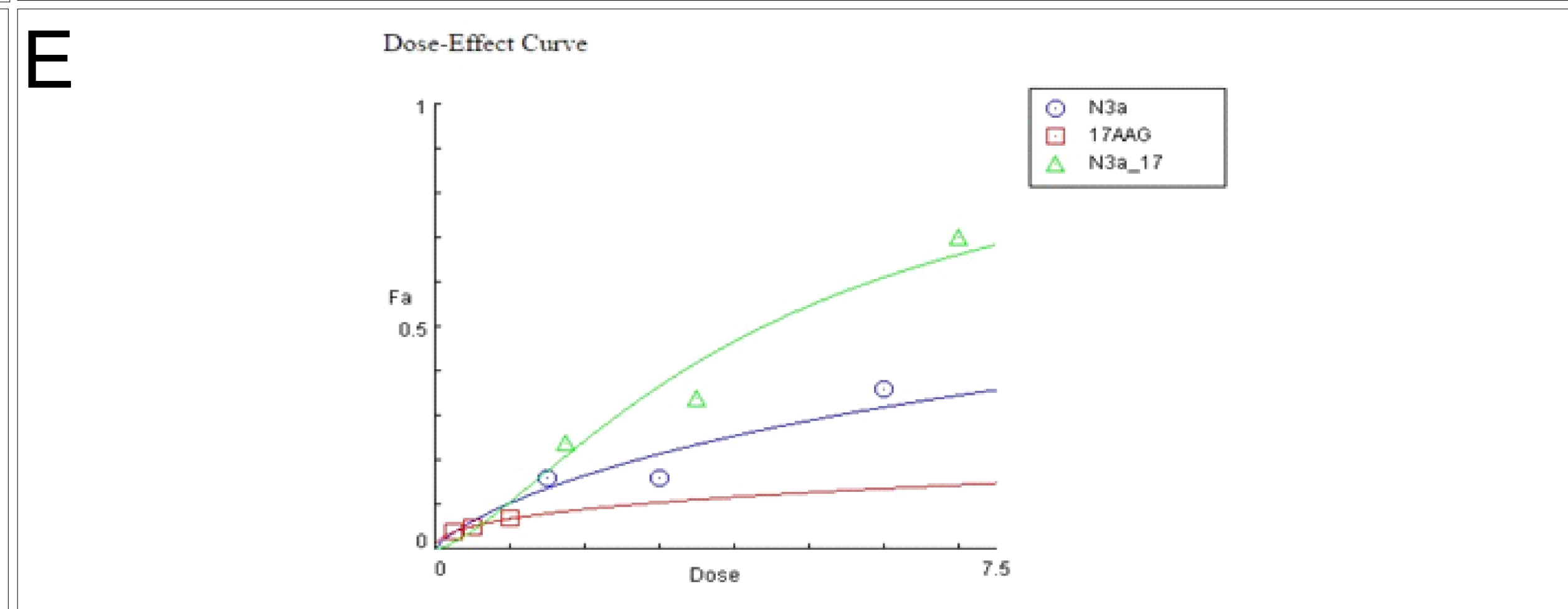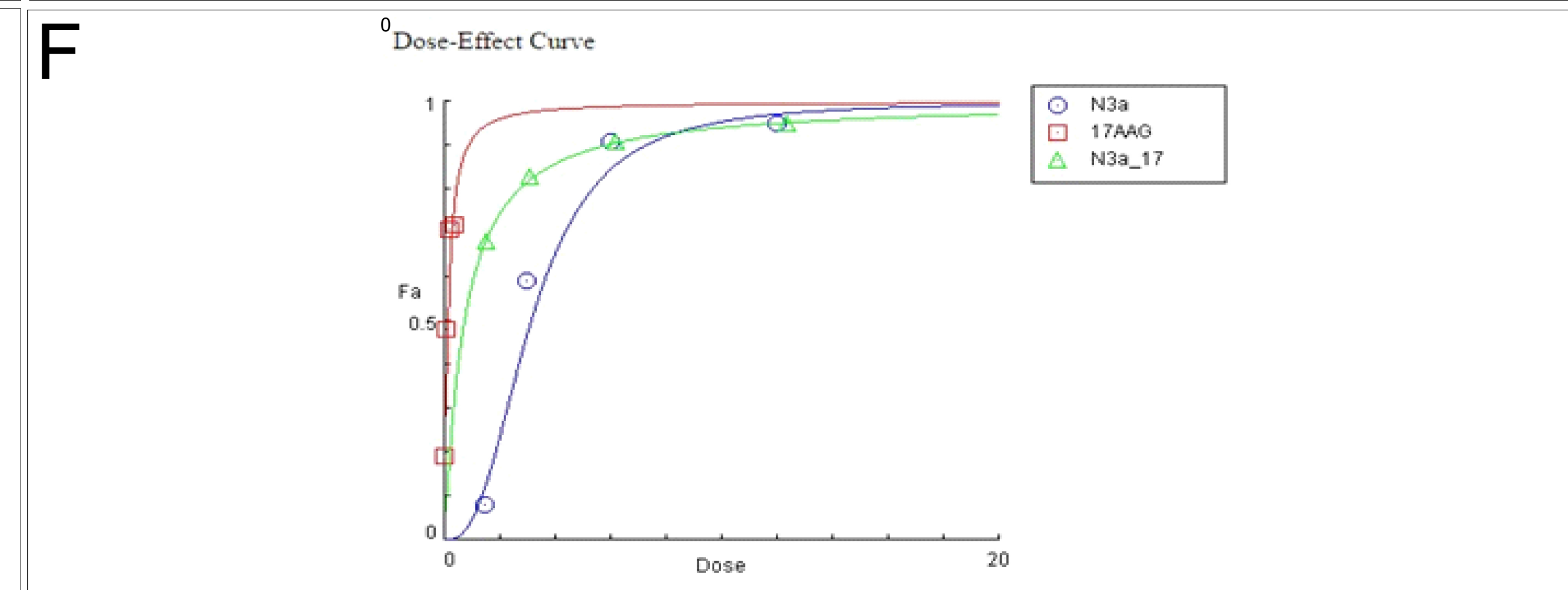

Supplement: Supplementary file 1 [file cancers-15-03903-s001.zip › Figure S8_Hsp90 inhibition.pdf]

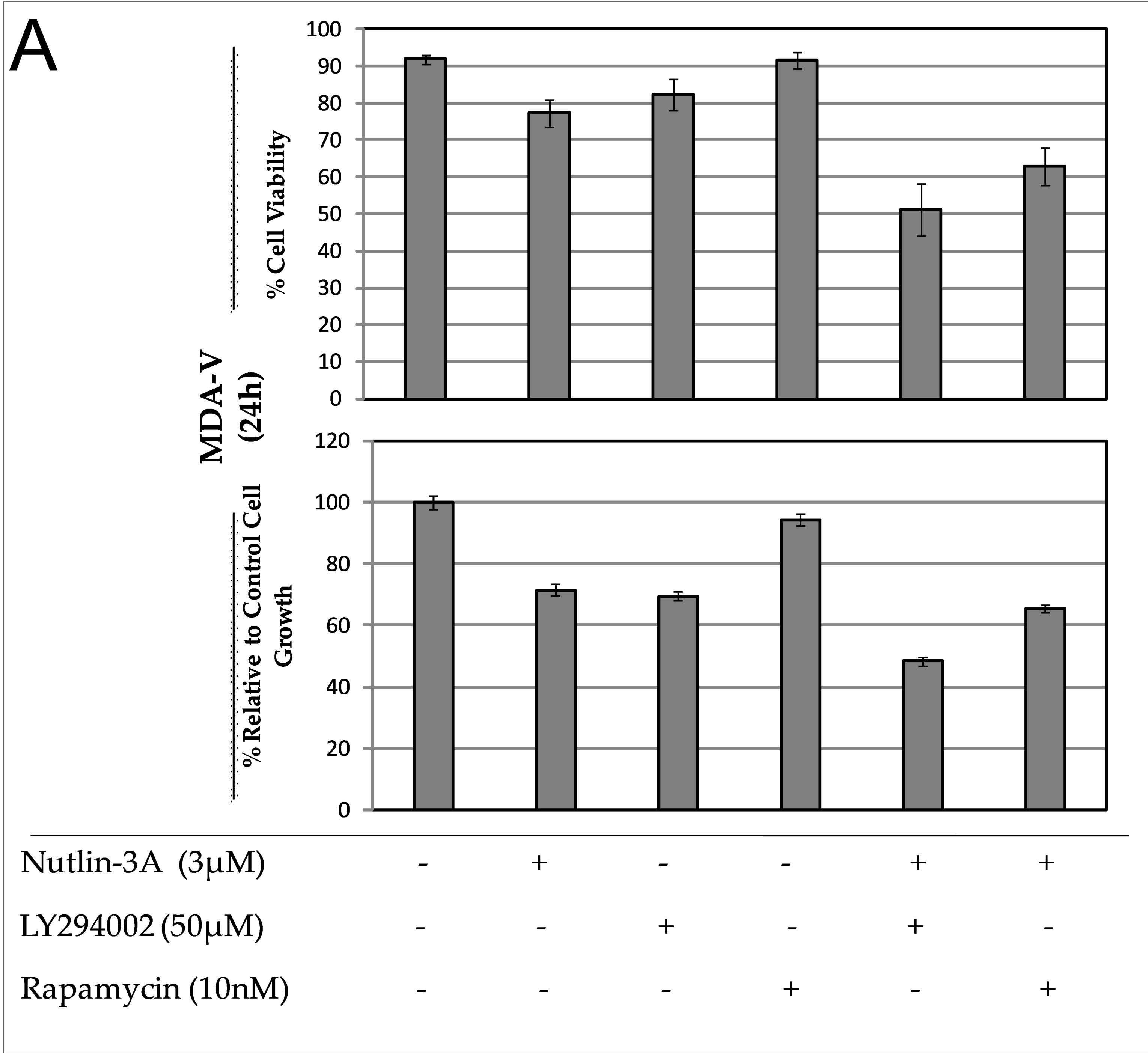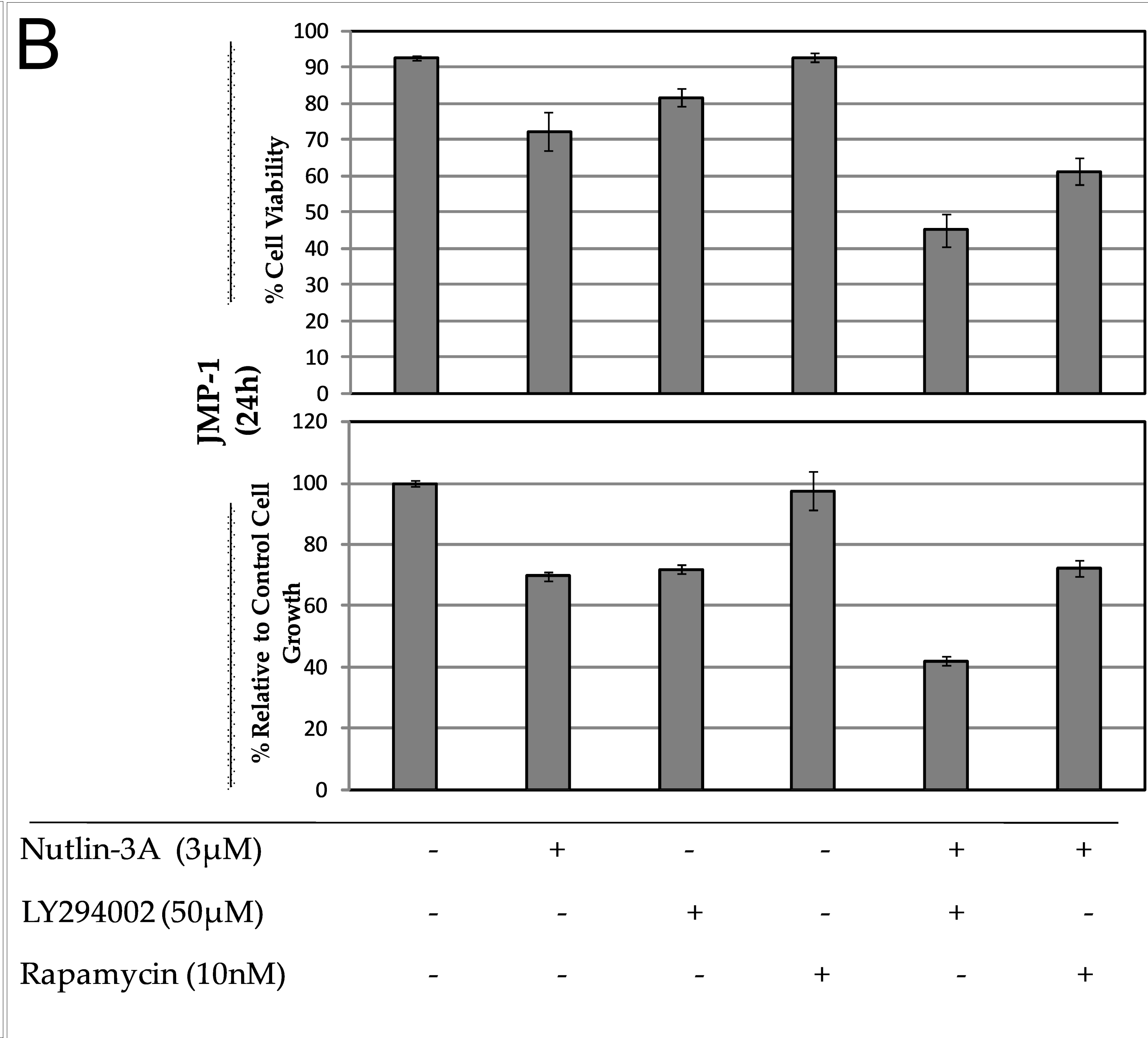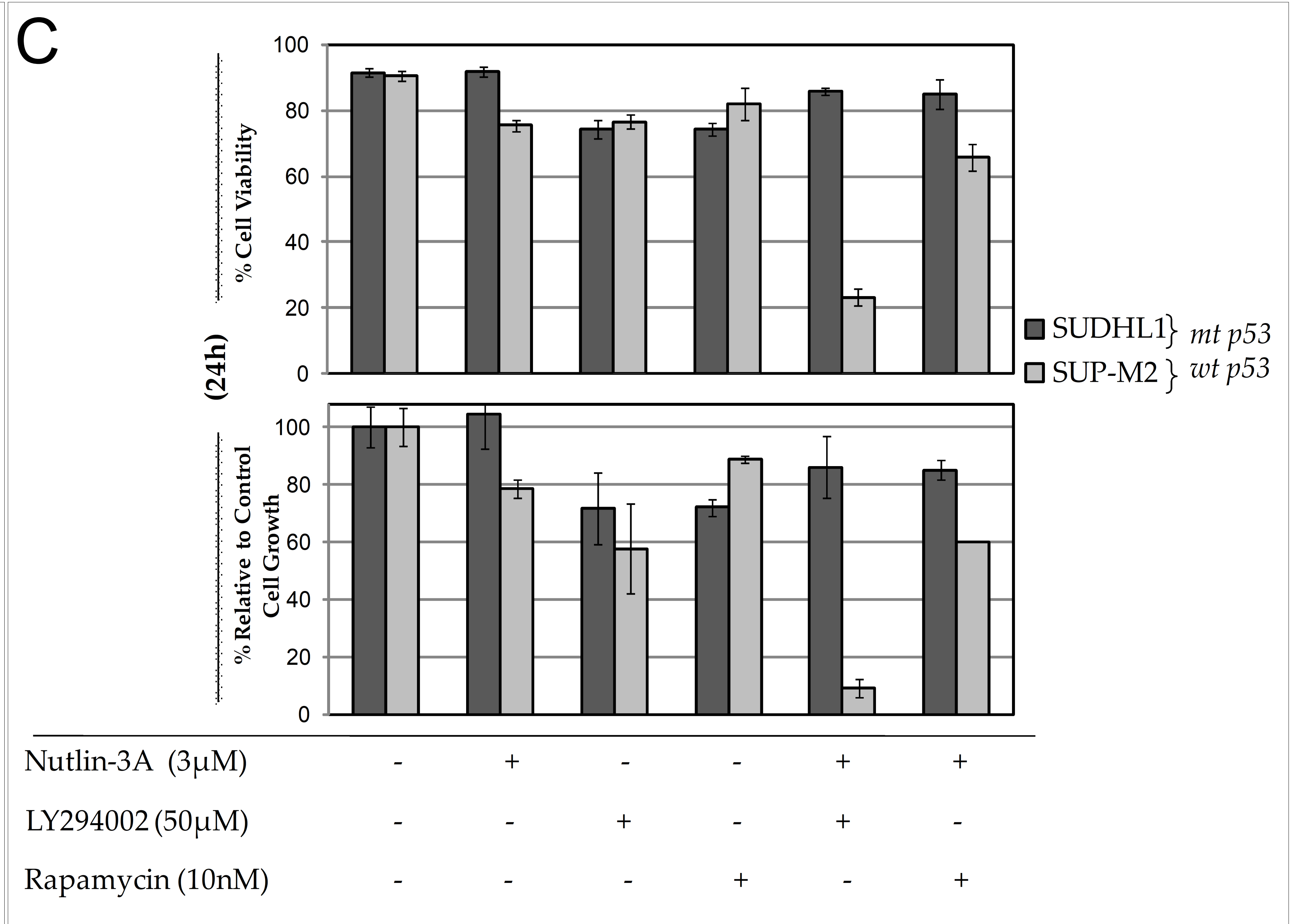

Supplement: Supplementary file 1 [file cancers-15-03903-s001.zip › Figure S9_mTOR inhibition.pdf]
